# Criteria for evaluating transferability of health interventions: a systematic review and thematic synthesis

**DOI:** 10.1186/s13012-018-0751-8

**Published:** 2018-06-26

**Authors:** Tamara Schloemer, Peter Schröder-Bäck

**Affiliations:** Department of International Health, Faculty of Health, Medicine and Life Sciences, CAPHRI–Care and Public Health Research Institute, Maastricht University, Postbus 616, 6200 MD Maastricht, The Netherlands

**Keywords:** Transferability, Evidence-based, Public health, Health care, Decision-making, Intervention, Assessment, Implementation, Evaluation

## Abstract

**Background:**

Improving the public’s health in different countries requires the consideration of diverse health care systems and settings. For evidence-based public health, decision-makers need to consider the transferability of effective health interventions from a primary context to their specific target context. The aim of this systematic review was to develop a model for the assessment of transferability of health interventions through identification and systematization of influencing criteria, including facilitators and barriers.

**Methods:**

A systematic literature search was performed in the databases PubMed, Embase, CINAHL, and PsycINFO. Articles were eligible if they were published in English or German and provided a description of transferability criteria. Included articles were ranked based on their thematic relevance and methodological support of transferability criteria. Using a qualitative approach, a thematic synthesis was conducted.

**Results:**

Thirty-seven articles were included in the review. The thematic synthesis revealed 44 criteria, covered by 4 overarching themes, which influence transferability of health interventions: The population (P), the intervention (I), and the environment (E) represent 30 conditional transferability criteria, and the transfer of the intervention (T) represents 14 process criteria for transferring the intervention to the target context. Transferability (-T) depends on the dynamic interaction of conditional criteria in the primary and target context as well as on the process of transfer. The description of facilitators and barriers deepens the understanding of the criteria. The synthesis resulted in two related models: the conceptual PIET-T model explains the underlying mechanism of transferability of health interventions and the PIET-T process model provides practical guidance for a transferability assessment.

**Conclusions:**

Transferability of health interventions is a complex concept, which needs systematic consideration of the primary and target context. It should be anticipated before and evaluated after an intervention is implemented in the target context. Therefore, decision-makers need systematic and practically relevant knowledge on transferability. The synthesized PIET-T conceptual and process models with systematized criteria, facilitators, and barriers are intended as a theoretical basis to determine transferability of health interventions. Further research is needed to develop a practical tool for the PIET-T models and to evaluate the tool’s usefulness for decision-making processes and intervention transfer.

**Electronic supplementary material:**

The online version of this article (10.1186/s13012-018-0751-8) contains supplementary material, which is available to authorized users.

## Background

Improving the public’s health is a concern in most countries. During the last two decades, advances of evidence-based medicine have led to the development of evidence-based approaches in the fields of public health and health care, with the purpose of improving population health and health services [1–6]. Various benefits are expected to result from the increased use of evidence-based public health (EBPH); these benefits include improved information for decision-makers about best practice, a higher likelihood of implementation of successful policies and programs, and more efficient use of resources [2, 6].

User groups for EBPH are decision-makers at international, national, regional, and local levels, researchers on population health issues, practitioners, and stakeholders who will be affected by an intervention [2]. Decision-makers must assess which evidence-based health interventions will best improve the health of a target population, a subgroup or a person, with respect to a specific health problem. Therefore, decision-makers must predicate intervention design on the best available scientific evidence and engage target populations or persons in their respective, specific contexts [2, 6]. In order to make evidence-based decisions, it is therefore necessary to assess whether a health intervention whose effectiveness was established in another context, is transferable to the decision-maker’s own specific context. More specifically, the question of transferability arises, which means the extent to which the outcomes of a successful health intervention evaluated in a primary context can be achieved in a target context [7–10]. The term ‘health intervention’ is applied independently of professions, thereby addressing different decision-makers. It is an umbrella term covering any measure or act “performed for, with or on behalf of a person or population whose purpose is to assess, improve, maintain, promote or modify health, functioning or health conditions” [11]. This means that health interventions can be implemented on different levels and cover a range of measures, such as policies, programs, and resource distribution approaches [12]; community health interventions [13]; and diagnostic, therapeutic, cognitive, and other health care interventions and services [14].

In contrast to the transferability of a health intervention, generalizability refers to the perspective of the researcher who makes statements about the extent to which the results of a given study are potentially generalizable to a wider or unspecified population, to another setting, or another time [8]. Because health interventions are complex and thus (their successes) are influenced by many factors, the specific context, in which an intervention is to be applied, plays a crucial role for evidence-based decision-making [15]. Thus, in order to assess the transferability of a health intervention to a specific context, a generalization of study results is often not sufficient. Practice has shown that decision-makers have mistakenly assumed that a health intervention, which works in one context, will automatically produce the same results in their context [16, 17].

Examples for failures in transferability are the school-based substance abuse program ‘Reconnecting Youth’ in the USA, which achieved more harm than good when replicated under real-world conditions [16, 18–21] and the implementation of the evidence-based assertive community treatment (ACT) in the UK, which showed no clinical gains for ACT clients and no reductions in the need for in-patient service [22–24]. Such failures underscore the importance of the concept of transferability for EBPH. Indeed, health policy decisions are commonly informed by research conducted in other contexts than the target context [7]. However, the literature rarely addresses the concept of transferability of health interventions from a primary context to a specific target context. Moreover, there is little information available pertaining to the perspective of decision-makers who seek solutions to health problems in target contexts. Decision-makers need information about the criteria that may influence the transferability of health interventions. Several authors have expressed the need for a validated list of attributes, a framework, or tool for the assessment of transferability [7, 8, 10, 25].

There are already initial descriptions of transferability criteria. However, these have several limitations. Existing reviews on transferability criteria either refer exclusively to transferability of interventions in health education [8] or do not differentiate between transferability and other concepts, such as external validity and applicability. Rather, a variety of related concepts is included to extract transferability criteria, such as generalizability, external validity, feasibility, and translation [7]. It is necessary, however, to distinguish these terms from transferability, as already shown above for generalizability. Unlike transferability, external validity focuses on study characteristics which are a basis for generalizability of study results to an unspecified context [8]. Feasibility refers to the applicability of a health intervention in a target context with respect to the implementation process [10]. Translation is a synonym for the term transfer which means ‘to carry across’ and focuses on the process of intervention transfer, e.g., on how an intervention is implemented and applied in practice [26]. In addition, existing reviews mainly provide a descriptive summary by categorizing criteria without using a specific analysis method, which goes beyond the description of primary frameworks and allows theoretical modeling.

Thus, it remains unclear how transferability of health interventions can be conceptually modeled to explain the underlying mechanism. It is also uncertain which criteria influence transferability to form a more comprehensive theoretical conception for the assessment of transferability of health interventions. Furthermore, it is unclear whether criteria must be considered separately according to different work fields or can be generalized. In other words: a sound theoretical basis to explain the concept and practically guide an assessment of transferability of health interventions is still missing. The objectives of this systematic review, therefore, were (I) to develop a theoretical model for the assessment of transferability of health interventions through identification and systemization of criteria for transferability, and (II) to explore the potential consequences of these criteria in the form of facilitators or barriers for transferability of health interventions.

## Methods

### Methodological approach

A qualitative approach was chosen for this systematic review. Dixon-Woods et al. [27] distinguish two main approaches for synthesizing evidence in systematic reviews: integrative syntheses are often used in quantitative reviews. They summarize data of largely proven and well-specified concepts, forming categories of summarized, extracted data. In contrast, interpretive syntheses aim to develop concepts inductively, as well as to form theories that integrate these concepts [27, 28].

As the literature does not provide a well-established body of evidence on the concept and the criteria for transferability of health interventions, it was necessary to use a systematic review method that allows to be conceptual in process and output in order to develop a basic model for the assessment of transferability. Therefore, a thematic synthesis—as a form of an interpretive synthesis—was performed [29]. This inductive method shares similarities with approaches from grounded theory and meta-ethnography, involving coding with the use of reciprocal translation and constant comparison for the development of descriptive and analytical (or higher-order) themes. Rooted in critical realism, the underlying assumptions suggest that the synthetic products are reproducible and that they correspond to a shared reality. The advantage of this method for the fulfillment of the objectives of this review is its characteristic of designing the synthesis products to inform policy and practice [30].

The conduct and reporting of this review were guided by the Enhancing Transparency in Reporting the Synthesis of Qualitative Research (ENTREQ) Statement [31]. This review was not registered on PROSPERO, which focuses on health-related outcomes, not conceptual development.

### Search strategy

As recommended for a thematic synthesis, the search was pre-planned [29]. In order to find the most appropriate search algorithm, many combinations of search terms were piloted, as is proposed by Jackson and Waters [32]. For more details on the search strategy, see Additional file 1.

The following final combination of search terms was entered into the databases PubMed, Embase, CINAHL, and PsycINFO on 6 June 2016: (“transferability”) AND (“health” OR “health care” OR “policy” OR “prevention” OR “service” OR “intervention” OR “program” OR “programme” OR “implementation”). The selection of these databases and search terms sought to optimize comprehensiveness with precision. In addition, all references of the articles included in the thematic synthesis were screened, which is recommended to identify articles that would otherwise be missed through a process of snowballing [33].

De-duplication of the search results was performed with the Systematic Review Assistant-Deduplication Module (SRA-DM), which has shown good sensitivity (84%) and specificity (100%, no false positive results) [34]. All duplicates were compared and checked before deletion. The remaining search results were imported into Endnote (Version X7) for the selection of articles.

### Selection of articles

To be eligible for the thematic synthesis, articles had to

1. Provide a description of transferability by using the exact term or a synonymous description which is in line with the following definition: transferability refers to the extent to which the outcomes of a successful health intervention evaluated in a primary context can be achieved in a target context. Articles with a synonymous description were eligible when the term transferability was not used, but the descriptions had the same meaning as the definition (for examples, see Additional file 1). Following the definition, articles were only eligible if they addressed the transferability of health interventions to target contexts. Descriptions addressing only the generalizability of research outcomes were not sufficient for the inclusion of articles.
2. Describe criteria and/or facilitators and/or barriers for transferability. Criteria are understood as influences on transferability of health interventions, measured by instruments or tools for assessing transferability, empirically investigated by quantitative or qualitative or mixed-methods studies, or described in review papers or in methodological, thematic, or discussion papers. Facilitators and barriers are descriptions of positive or negative consequences for transferability.

Because transferability is a rarely described concept, no restriction was made to the type and date of published articles or to a specific study design. Gray literature, conference abstracts, or other abstracts where no published article was available were excluded.

Published articles were not eligible if

1. The description of transferability was not in line with the definition provided above (i.e., transferability had another meaning);
2. Transferability was not one main topic of the article or transferability as a concept was not (at least synonymously) described, defined, explored, operationalized, or measured by transferability criteria (including facilitators or barriers for transferability);
3. They exclusively addressed specific conditions in one or more developing country(ies), in order to provide a basis for the comparability of the transferability criteria;
4. They exclusively focused on the transferability of statistical calculations of economic evaluations;
5. They were written in a language other than English or German.

The screening of titles and abstracts according to the eligibility criteria was supported by a rating system of relevance for the research objectives. Full texts of all potentially relevant abstracts were read and rated for the final inclusion of articles. The screening process was conducted by one author (TS). All steps for the inclusion of articles were discussed between both authors. In addition, the full texts of all potential articles identified through snowballing, including those which described terms related to transferability, were checked before and after analysis to ensure that no relevant criteria for transferability were overlooked and that saturation of the identified criteria was reached.

### Quality ranking

In order to classify criteria in terms of their relevance to the assessment of transferability of health interventions, the authors developed a quality ranking scale based on the quality assessment strategy for criteria of external validity provided by Dyrvig et al. [35]. The first condition for the quality ranking was the precision and richness of the description of criteria for transferability of health interventions. The second condition was the extent of support for the transferability criteria with regard to the methodology of the article (empirical, literature, consensus support, or no methodological justification).

Based on these conditions for quality, the ranking system was developed and applied by one author (TS), reaching from 1 to 10. The ranking system determined the order of analysis for the thematic synthesis to achieve as much accuracy and credibility as possible. For more details on the quality ranking, see Additional file 1. In order to facilitate the reader’s access to information, three relevance levels were determined from the ranking, which indicate whether the article has high, medium, or low relevance for the analysis. The quality ranking for each article can be found in Additional file 2: Table S1.

### Data analysis

As a basis for the thematic synthesis the following information was extracted from the articles: authors, year of publication, title, the type of transferability of health interventions relating to the main field (e.g., health promotion, prevention, health technology), and the support category. Further, the description of transferability was documented for each article. The thematic synthesis was structured according to the quality ranking. Details for transparency of the method can be found in Additional file 1.

#### Stages of the thematic synthesis

The analysis was conducted in three stages as recommended by Thomas and Harden [29]. Stages 1 and 2 include a free line-by-line coding of text and the organization of the codes into related areas for the construction of descriptive themes [29]. Initial codes were created, which represented criteria for transferability of health interventions. Thereby, rules were established to improve credibility of the analysis (see Additional file 1) [36]. Consistency of interpretation/assignment and the need for new levels of coding of criteria were checked continuously [29].

For some of the criteria, sub-criteria emerged. All resulting criteria were grouped into a hierarchical structure for the development of descriptive themes (stage 2; [29]). For verification of the criteria and descriptive themes by the original articles, all steps were documented in detail together with corresponding references. All initial material resulting from each step was rechecked to ensure consistent allocation against the themes.

For the identification of facilitators and barriers, the same procedure was used. The extraction of facilitators and barriers aimed at providing a deeper understanding of the criteria. In addition, steps of an assessment of transferability emerged from the data. These were extracted and grouped in the same way as described before.

Stage 3 is the step of going beyond the findings of the included articles [29, 36]. It was used to build higher-order themes out of the descriptive themes and to conceptually model their relationships and the mechanism which underlies transferability of health interventions in order to create a meaningful whole out of the findings and to provide a theoretical conception of transferability [36]. Furthermore, a process model was developed based on the thematic steps for determining transferability and was brought together with the identified criteria of transferability of health interventions.

## Results

### Selection of articles

**Fig. 1:**
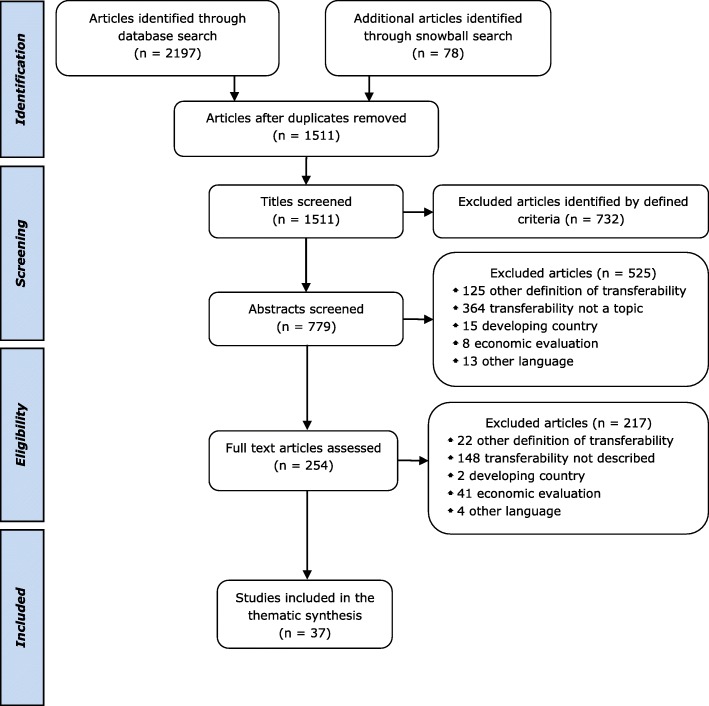
Flowchart of study selection. As shown in Fig. 1, a total of 2275 potential journal articles were identified by searches, of which 78 were found through the screening of references. Seven hundred sixty-four duplicates were removed. Of the 1511 remaining articles, 1474 were excluded due to the defined eligibility criteria. The reasons for exclusion were documented for each investigated abstract and full-text, namely, another definition of transferability, transferability was not a topic of the article, the article addressed a developing country, exclusively focused on transferability of economic evaluations, or was written in a language other than English or German. Finally, 37 articles were included in the thematic synthesis, of which 9 were found through snowball sampling

As shown in Fig. 1, a total of 2275 potential journal articles were identified by searches, of which 78 were found through the screening of references. Seven hundred sixty-four duplicates were removed. Of the 1511 remaining articles, 1474 were excluded due to the defined eligibility criteria described in the “Methods” section. The reasons for exclusion were documented for each investigated abstract and full-text (see Fig. 1). Finally, 37 articles were included in the thematic synthesis, of which 9 were included following snowball sampling.

### Characteristics of included articles

The articles were published between 1999 and 2016. Table 1 summarizes the number and types of articles identified with regard to the main work fields. The articles were inductively grouped in reference to their specification of transferability (transferability type). Nine articles addressed the transferability of health care services. These articles were concerned with the improvement of services in health care, such as mental health care or clinical services. Nine further articles were specifically related to the transferability of health promotion and prevention interventions, for example, for healthy nutrition or cancer prevention. Some articles (8) referred to transferability of evidence in general. That means, these articles had a focus on transferability of research results, but did not specify interventions in a work field. Seven articles addressed transferability of public health interventions or programs in general without specifying these, and 4 articles specifically referred to health technology, mainly in the form of tools for assessing transferability of health technology in general. There was much variation across articles concerning the article types and study designs. Four studies used a qualitative design and 5 a mixed methods design. Two methodological papers were based on empirical studies. Further, 5 reviews, 1 assessment tool, 1 study protocol, 8 literature-based methodological papers, and 11 thematic or discussion papers were included. Additional file 2: Table S1 provides an overview of the characteristics of each article with regard to author and year, title, transferability type, and the quality assessment.

**Table 1:** Number of articles per transferability type and article type/study design

Transferability type	Number	Article type/study design
Health care services	9	1 qualitative research 2 methodological papers based on empirical research 1 study protocol 5 thematic or discussion papers
Health promotion and prevention	9	2 mixed-methods studies (1 tool) 2 qualitative researches 1 review 4 methodological papers
Findings/evidence in general	8	1 qualitative research 2 reviews 1 methodological paper 4 thematic or discussion papers
Public health interventions/programs, not specified	7	1 review 1 assessment tool 3 methodological papers 2 thematic or discussion papers
Health technology/ intervention	4	3 mixed-methods studies (tools) 1 systematic review and workshops

### Quality assessment

All articles were assessed and ranked according to their description of transferability and their methodological support for the extraction of transferability criteria (see Additional file 2: Table S1). Of the 37 included articles, 11 (30%) provided empirical support, 4 articles additionally showed literature support, and 2 additionally showed consensus support. Twenty-three articles (62%) were based on literature support, of which 3 additionally had consensus support. Three articles (8%) provided no defined background. Regarding the relevance for the analysis, 15 articles (41%) were ranked between 1 and 4, and thus had high relevance. Thirteen articles (35%) were ranked with medium relevance (ranking of 5–7) and 9 (24%) with low relevance (ranking of 7–10).

### Results of the thematic synthesis

The presentation of the synthesis results is structured according to four higher-order themes, which were derived from the three stages of the analysis: the population (P), the intervention (I), and the environment (E) represent conditional criteria for the transferability of health interventions, and the transfer of the intervention (T) represents process criteria for transferring the intervention to the target context. Criteria underlying these four higher-order themes influence the transferability of health interventions.

In sum, 44 criteria and 62 sub-criteria were derived from 867 free line-by-line codes of text through constant comparison and translation. All resulting criteria were thematically grouped to build 14 descriptive themes. A descriptive theme represents the topic of a group of criteria in a hierarchical structure (e.g., the criterion *conception of the intervention in the primary and target context* underlies the descriptive theme *intervention content*). Sub-criteria further explain a criterion (e.g., the sub-criterion *tools and materials* used for the intervention further describes the criterion *conception of the intervention in the primary and target context*). The four higher-order themes introduced above are the overarching themes, which were built in stage 3 on the basis of stages 1 and 2 by systematically comparing the meaning of the descriptive themes and mapping their relationships. The higher-order themes represent the descriptive themes and criteria (with sub-criteria) (e.g., the criterion *conception of the intervention in the primary and target context* with the sub-criterion *tools and materials* underlies the descriptive theme *intervention content*, which is grouped under the higher-order theme *intervention*). The final step of modeling in stage 3 was conducted in an analytical, cyclic process by analyzing and interpreting key findings from all three stages of the analysis. A deeper explanation of these stages can be found in Additional file 1.

**Fig. 2:**
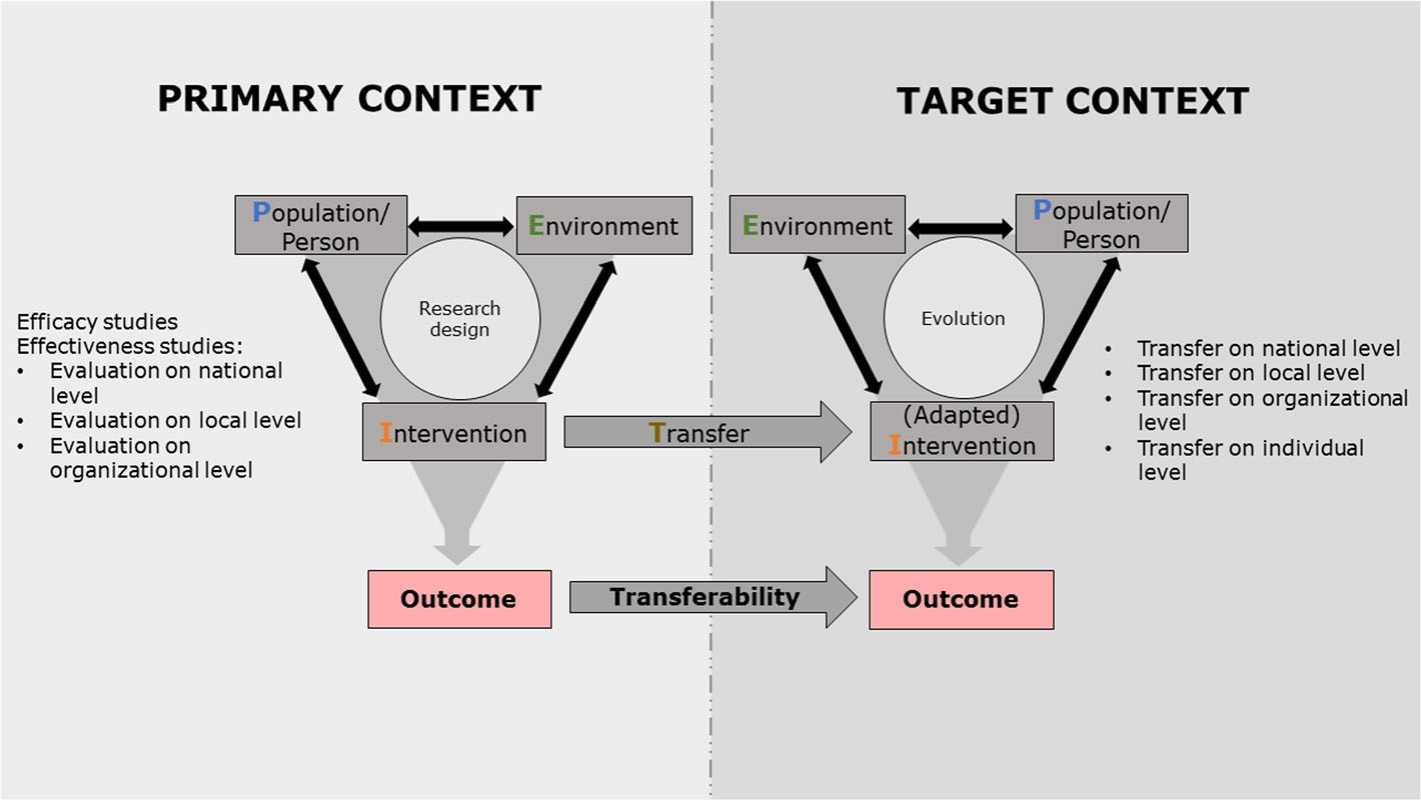
The conceptual Population-Intervention-Environment-Transfer Model of Transferability (PIET-T) focuses on the perspective of the decision-maker, who seeks to improve the health situation of the target population (or person) and aims to transfer an intervention from a primary context to the target context. It is assumed that the population (P), the intervention (I), and the environment (E) in the primary and the target context influence one another. The combination of these three constructs determines the resulting outcome. The decision-maker collects information on the evidence established in a primary context. The primary context symbolizes the form in which evidence was gained and is available. In order to decide on the transfer of the intervention, the decision-maker needs to take into account the conditions of the primary context and his or her own context. The transfer can take place on different levels in the target context, for example, the national level, the local or community level, the organizational level, or the individual level. Therefore, the research design and the three constructs of the primary context should be compared with the level of transfer and the three constructs in the target context. Considering transferability, an adaptation of the intervention to the target context may be necessary. The information gained from the primary context influences how the transfer is designed. At the same time, designing and realizing the transfer requires the consideration of the constructs of the target context, since transferability also depends on the interaction of these three constructs. Therefore, the population, the intervention, and the environment in the primary and target context as well as the transfer itself influence transferability of health interventions

Two models were built from the criteria, descriptive themes, and higher-order themes. Figure 2 shows the conceptual model for the mechanism of transferability derived from the analysis, which forms the theoretical basis for the assessment of transferability of health interventions. In the following section, we will present this conceptual model, which is based on the four higher-order themes. After that, we will explain the second model, a process model for the assessment of transferability, which contains the descriptive themes and criteria. Finally, we provide an overview over all descriptive themes, criteria, and sub-criteria in Table 2.

#### Conceptual model of higher-order themes

The synthesized conceptual Population-Intervention-Environment-Transfer Model of Transferability (PIET-T) presented here focuses on the perspective of the decision-maker, who seeks to improve the health situation of the target population (or person) (P) and aims to transfer an intervention (I) from a primary context to the target context. Decision-makers may be, for example, a group of policy-makers, researchers and experts, or leaders of an institution or professionals. A decision-making process can extend to the needs and views of the target population (person) and the coordination players involved in the target environment (E) in order to decide on, plan, and realize the transfer (T).

The conceptual PIET-T model shows two contexts, the primary context and the target context. It is assumed that the population, the intervention, and the environment in each of these contexts influence one another. The combination of these three constructs determines the resulting outcome.

The decision-maker collects information on the evidence established in a primary context. The primary context symbolizes the form in which evidence was gathered and is available. This evidence can relate to efficacy research, for example, in the form of guidelines or a systematic review or a randomized controlled trial with highly controlled conditions; or it can take the form of effectiveness research, which takes place in one or more national, local, or organizational contexts. Research evidence can be synthesized from different studies with several research contexts or it can be gathered from a single study with one research context. A research context is understood as a system with unique characteristics (criteria) of the population, the intervention, and the environment. On the one hand, the characteristics of the population, the environment, and the nature of the intervention in this context influence the conduction of research and its design. On the other hand, the research design influences how the population is chosen, how the intervention is carried out, and how the environment is controlled. Regarding the primary context, the description of the evidence therefore refers to the reporting of outcomes of one or more studies on an intervention, including the description of the specific study design and of relevant criteria of the study population, the intervention, and the study environment. This description of the evidence largely determines what information the decision-maker receives to decide whether or not the intervention is appropriate for improving the health of the population in the target context.

In order to decide to transfer the intervention, the decision-maker must take the conditions of the primary context described above and his or her own context (i.e., the target context) into account. The transfer can take place on different levels in a target context, for example, the national (or even multinational) level, the local (regional or community) level, the organizational level, or the individual level. Therefore, the research design and criteria of the three constructs population, intervention, and environment in the primary context should be compared with the level of transfer and criteria of the three constructs in the target context. For example, highly controlled contextual conditions of a randomized controlled trial (primary context) are compared with the real-world contextual conditions in a target community. Outcome-relevant differences shown by transferability criteria are taken into account for decision-making and potential intervention transfer.

The conceptual PIET-T model frames the target context as an own system, with unique characteristics of the target population and unique environmental conditions. A decision-maker cannot expect transferability of an intervention only from the outcomes of the primary evidence. The transferred intervention, the population, and the environment of the target context influence one another. Therefore, it may become necessary to adapt the intervention to the target context. When the intervention is transferred, an evolution takes place in the target context, which addresses the target population and the environment. The decision-maker should anticipate changes and reactions in the target population and the environment, which may, in turn, lead to adaptations and further development of the intervention. In other words, the evolution emphasizes the dynamics of the target context as a developing system over time with a mutual influence of the population, the environment, and the transferred intervention.

The outcome is the result of a change process in the target context and therefore also reflects it. The knowledge about this result can, in turn, trigger a change process. Therefore, it is important to take a time component into account when evaluating the transferability of health interventions.

This mechanism, which is shown in the model, influences the transferability of the results. Both the conditions of the primary and the target context determine the transferability of outcomes. The information gained from the primary context influences how the transfer is designed. At the same time, designing and realizing the transfer requires the consideration of the constructs of the target context, since transferability also depends on the interaction of these three constructs. Therefore, criteria of the population, the intervention, and the environment in the primary and target context as well as the transfer itself influence transferability of health interventions. This leads to the hypothesis that the more both contexts resemble each other against those criteria that determine intervention success, the more likely is the transferability of the intervention. This also means that the comparability of the outcome of the target context with the outcome of the primary context depends on the similarity of the constructs of both contexts and on the transfer. However, this does not mean that criteria and processes in the primary and target context must correspond exactly in order to ensure transferability. The change process in the target context is, as shown in the model, a separate process that produces its own results. It is therefore necessary to consider to what extent both contexts should actually be similar and to what extent it is possible to create successful interventions through adaptations with regard to intervention transfer. The model suggests that the reflections on transferability should focus more on whether and by which means it is possible to achieve intervention success in the target context than on “reproducing” the effects of the primary context, because contextual influences in the target context usually differ from influences in the primary context. These potential influences on transferability of health interventions in the form of criteria are described in detail in the following sections.

Because the model focuses on the perspective of the decision-maker, it does not show the interaction between research and practice. However, the dashed lines shown in Fig. 2 indicate that there is an exchange of information between the two contexts in the sense that the decision-maker uses the information of the primary context and, in turn, an evaluation in the target context contributes to knowledge by generating results and information about transferability. With this, the target context may become part of a primary context for other decision-makers. This mechanism is applicable at macro-, meso-, and microlevels.

#### Process model for the assessment of transferability

**Fig. 3:**
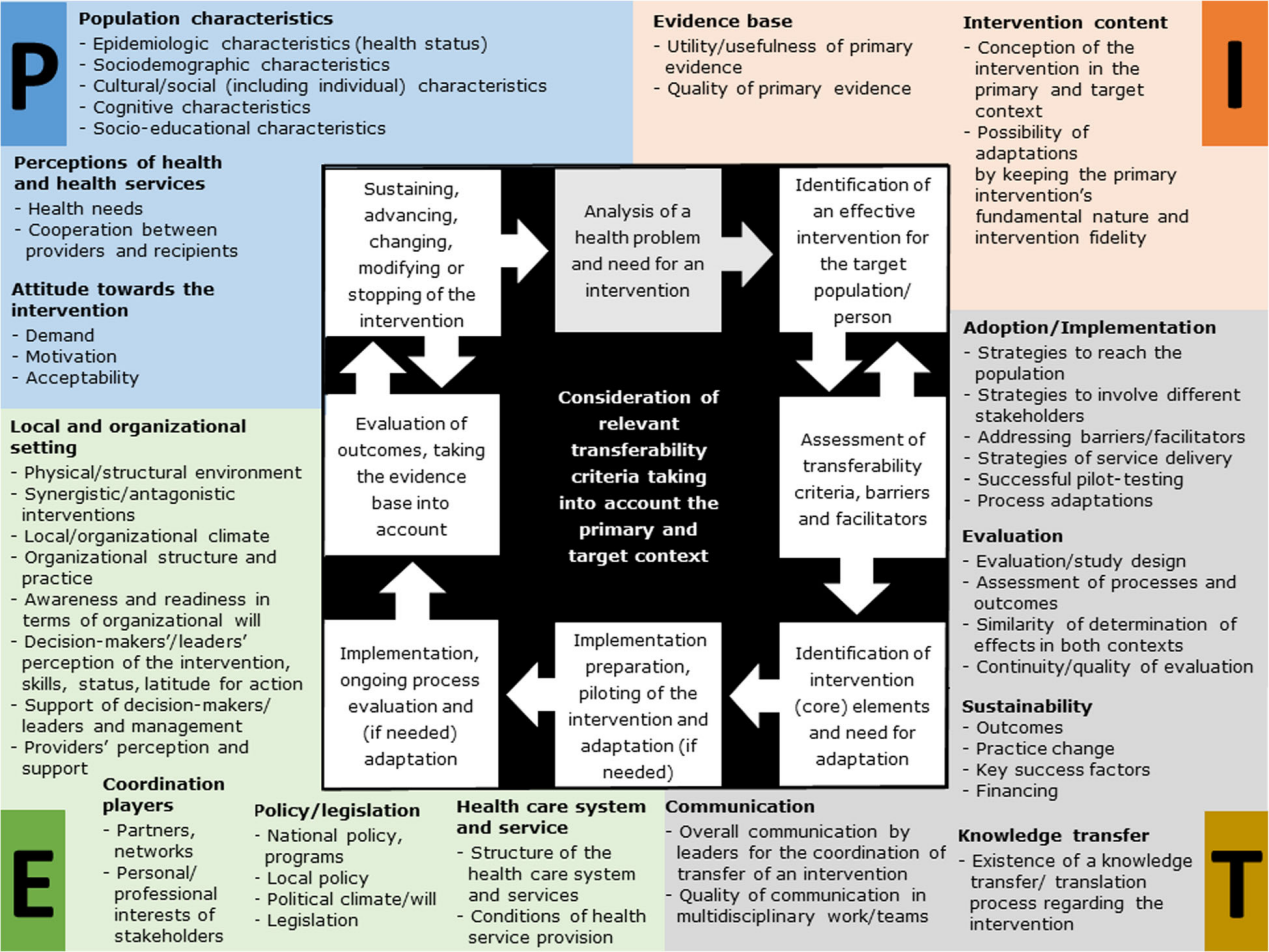
Process model for the assessment of transferability (PIET-T process model). Figure 3 shows the process of the assessment of transferability of health interventions derived from the analysis and includes descriptive themes and criteria of the population (P), intervention (I), environment (E), and transfer (T). The PIET-T process model is intended to accompany the steps for determining transferability: The analysis of the health problem is based on the (baseline) characteristics of the population in the target context in order to search for an effective intervention. Because transferability is dependent on the conditions in the primary and target context, a comparison of both contexts should be attempted. This requires both information from the primary context and from the target context. The themes and criteria, which are mapped around the process, are intended to help determine which information is relevant for the target context and for a comparison with existing information on the primary context. By assessing the criteria, facilitators and barriers can also be identified. However, transferability cannot be measured in this phase, but can only be anticipated using existing information. An identification of transferable (core) elements of the intervention and the need for adaptation may be relevant, depending on the complexity and character of the intervention and its conception, the population characteristics, and the environmental conditions. The steps of implementation and evaluation are well known steps of process models. Finally, transferability can only be assessed after evaluation by measuring the effectiveness of the intervention. The evaluation may lead to sustaining or advancing of the intervention, to changing of its (core) elements or modifying of specific aspects, or to stopping of the intervention

Figure 3 shows the process of the assessment of transferability of health interventions derived from the analysis (stage 3) and includes descriptive themes and criteria of the population (P), intervention (I), environment (E), and transfer (T). The PIET-T process model is intended to accompany the steps for determining transferability:

The analysis of the health problem is based on the (baseline) characteristics of the population in the target context in order to search for an effective intervention. Because transferability is dependent on the conditions of the primary and target context, it is beneficial to compare both contexts. This requires both information from the primary context and from the target context. The identification of similarities and differences between the two contexts is important (1) to decide whether and how (under what conditions) the intervention is suitable for improving the health of the target population and (2) to systematically plan the transfer process. The themes and criteria, which are mapped around the process, are intended to help determine which information is relevant for the target context and for a comparison with existing information about the primary context. Additional file 3 provides a guide on how to use the criteria for decision-making and planning of the intervention transfer.

The review of criteria may result in the need to search again for an effective intervention, for example, if information is not useful (information about relevant criteria may be missing from both contexts), or the quality of primary evidence is poor, or the intervention is not appropriate for the target population. By assessing the criteria, it becomes possible to identify facilitators and barriers, as each criterion may potentially hinder or promote transferability, depending on the conditions in the respective primary and target context. With this, barriers and facilitators for the process of intervention transfer can be identified as well. However, transferability cannot be measured in this phase, but can only be anticipated using existing information. Therefore, this initial assessment highly depends on the assessing persons and on the usefulness and quality of the available information from the primary and target context, which may require further data gathering and resources, or limit an assessment of transferability.

An identification of transferable (core) elements of the intervention and the need for adaptation may be relevant, depending on the complexity and character of the intervention and its conception, the population characteristics, and the environmental conditions.

The steps of implementation preparation, implementation, and evaluation are well known steps of process models. In order to determine transferability, it is useful to consider the extent to which reproduction of the intervention is meaningful and to what extent evolution takes place, as shown in the conceptual model. The criteria of transfer are intended as support for this, for example, the similarity of determination of effects in both contexts. The extent of evolution is also dependent on the ongoing process and the evaluation. Finally, transferability can only be assessed through evaluation by measuring the effectiveness of the intervention in the target context. The evaluation may lead to sustaining or advancing of the intervention, to changing of its (core) elements or modifying of specific aspects, or to stopping of the intervention.

#### Descriptive themes

The descriptive themes categorize the criteria, facilitators, and barriers for transferability of health interventions, which underlie the higher-order themes population, intervention, environment, and transfer. During the analysis of stages 1 and 2, it became clear that sorting criteria by transferability type was not relevant, as criteria were repeated by authors independent of the main work field. A generalization of the criteria was thus appropriate. The 14 descriptive themes, 44 criteria, and 62 sub-criteria are shown in Table 2. Additional file 4 provides the essential description of these results for an understanding of the criteria and important facilitators and barriers. Additional files 5 and 6 provide detailed tables (Tables S4 and S5) for the criteria with examples, and all identified facilitators and barriers.

The descriptive themes and criteria shown in the PIET-T process model and in Table 2 are intended to accompany the process of an assessment of transferability. Different criteria may be relevant at different times. Relevant criteria may be used before the transfer of an intervention for the comparison between primary and target context in order to make an initial assessment of transferability and to plan the next steps of the process (see Additional file 3). However, the criteria can also be used in the course of time in order to operationalize factors for the process evaluation or as a basis for a qualitative exploration on why transferability is given or not, that is, they may also be used retrospectively.

**Table 2:** Overview of descriptive themes, criteria, and sub-criteria

*Higher-order theme:* 1. Criteria of the population in the primary and target context	
*Descriptive theme:* 1.1 The population characteristics in the primary and target context in terms of the following: * Criteria:* …the epidemiologic characteristics [7–10, 17, 25, 39, 41, 46, 52, 53, 63, 67–72] …sociodemographic characteristics [7–10, 25, 37–39, 46, 52, 53, 55, 56, 67–71] …the cultural/social (including individual) characteristics [8–10, 25, 37–39, 41, 46, 52, 53, 55, 67, 69, 71] …cognitive characteristics [8–10, 25, 52] …socio-educational characteristics [8–10, 25, 37, 52, 55]	
* Descriptive theme:* 1.2 The population’s perceptions of health and health services in the primary and target context in terms of the following: * Criteria:* …the health needs (regarding the health problem) [7–9, 41, 46, 52, 53, 55, 67] …the cooperation between providers and recipients [9, 25, 37, 46, 47, 50, 52, 72]	
* Descriptive theme:* 1.3 The population’s attitude towards the intervention in the primary and target context in terms of the following: * Criteria:* …the population demand for the intervention [9, 17, 37, 55, 69] …the acceptability of the intervention [7–10, 25, 40, 47, 52, 55, 69] …the motivation [8, 9, 17, 25, 46]	
*Higher-order theme:* 2. Criteria of the intervention in the primary and target context	
* Descriptive theme:* 2.1 Characteristics of the evidence base for comparison of primary and target context in terms of the following: * Criterion:* …utility/usefulness of primary evidence particularly with regard to the following: * Sub-criteria:* • Level of transfer [7, 8, 16, 17, 26, 38, 39, 41, 45, 46, 56, 64] • Clearness and relevance of the research question/problem for decision-making [7, 40, 73] • Detailed description and relevance of the population/sample for decision-making [25, 37, 45, 48, 72, 73] • Relevance of the outcome measurement for the target population and environment [7, 37, 49, 73] • Up-to-dateness of the intervention and relevance of the results for decision-making [7, 8, 10, 17, 25, 37, 39, 40, 53, 64, 72, 73] • (Anticipated) Applicability of the intervention to the target population/groups and setting [7, 8, 10, 37, 39, 40, 74] • Sufficient description of environmental conditions, processes, results, and the intervention [7, 9, 10, 17, 18, 25, 26, 37–40, 45, 47, 48, 55, 56, 68, 73] • Availability of documents and tools [8, 9, 17, 46, 47] * Criterion:* …quality of primary evidence particularly with regard to the following: * Sub-criteria:* • Number of studies on the intervention and consistency of the results [7, 16, 37, 39, 48, 64, 69, 73, 75] • Study design/study type and appropriateness for the research question [7, 8, 37–39, 41, 48, 53, 69, 72, 73] • Appropriateness of sampling according to the study design [7, 8, 16, 25, 37, 48, 63, 72, 73] • Ethical considerations [8, 16, 48, 73] • Appropriateness and rigor of measurement/data collection, assessed in accordance with the study design [7, 16, 37, 48, 53, 73, 75] • Appropriateness and rigor of evaluation/data analysis, assessed in accordance with the study design [8, 16, 25, 37–39, 48, 53, 73] • Bias and/or confounding under consideration of the study design [16, 37, 41, 69, 72, 73] • Appropriateness of interpretation of the results, e.g., of statistical tests/quantitative analyses, and presentation of the results [7, 8, 10, 16, 25, 37, 45, 72, 73, 75] • Generalizability/external validity [7, 8, 10, 16, 17, 25, 37, 39, 45, 46, 48, 72–74] • Level of evidence and/or grade of recommendation for adoption [16, 37, 69, 72, 73]	
* Descriptive theme:* 2.2 Characteristics of the intervention content in the primary and target context in terms of the following: * Criterion:* …the conception of the intervention in the primary and target context particularly with regard to the following: * Sub-criteria:* • The complexity/character of the intervention [7–10, 16, 18, 26, 37–39, 45, 52, 53, 56, 60] • Theoretical foundations or model and/or principles/methods and components [8, 9, 37–40, 47, 48, 53] • The action plan for the transfer process [7–10, 37, 38, 47, 52] • Tools and materials [9, 40, 47] • Scale/reach and duration of the intervention [8, 9, 38, 45, 52, 67] • Costs of the intervention [7, 8, 25, 38, 45, 52, 55, 67, 69, 73, 75] * Criterion:* …the possibility of adaptations [7–9, 26, 38, 40, 45–47, 52, 53, 55, 56, 64, 68, 70] by keeping the primary intervention’s fundamental nature and intervention fidelity [7–9, 16, 17, 25, 38, 40, 45–47, 53, 56, 68, 70] particularly with regard to the following: * Sub-criteria:* • Identification of transferable core elements/key functions [17, 46, 47, 52–56] • Identification of elements which are not transferable or need modification [46, 56] • Adaptation/modification of the specific form of the intervention [7, 8, 38, 40, 46, 47, 53, 55, 56, 64, 68, 70]	
*Higher-order theme:* 3. Criteria of the environment in the primary and target context	
* Descriptive theme:* 3.1 Characteristics of policy and legislation in the primary and target context in terms of the following: * Criteria:* …national policy and political programs [7, 8, 18, 38, 50, 56, 71] …political climate and will [7, 10, 37, 38, 60, 64, 69] …local policy [7, 8, 18, 37, 39, 56] …legislation relevant to transferability of the intervention [18, 75]	
* Descriptive theme:* 3.2 Characteristics of coordination players in the primary and target context in terms of the following: * Criteria:* …types of partners, networks, and their (formal or informal) involvement [9, 16, 18, 37, 38, 40, 45–47, 49, 50, 52, 56] …different personal and professional interests of stakeholders [8, 16, 18, 25, 37, 40, 56, 60]	
* Descriptive theme:* 3.3 Characteristics of the health care system and service provision in the primary and target context in terms of the following: * Criterion:* …the structure of the health care system and inherent services particularly with regard to the following: * Sub-criteria:* • Organization [17, 18, 25, 38, 50, 52, 68, 70, 71, 75] • Financing system [8, 18, 26, 38, 41, 69, 71] • Alternative interventions available [8, 17, 25, 69] * Criterion:* …conditions of health service provision particularly with regard to the following: * Sub-criteria:* • Usual care conditions and treatment as usual [7, 8, 16, 17, 25, 38, 41, 46, 47, 53, 55, 69] • Professional expertise regarding the health problem and the new intervention [8–10, 16, 25, 26, 38, 39, 45, 47, 52, 68–70] • Financial resources and conditions of intervention funding [7, 9, 18, 25, 38, 46, 52, 56, 64, 68, 70, 71, 75] • Resources for intervention delivery (availability and need) [7–10, 16, 25, 37, 39–41, 45, 47, 52, 53, 55, 68–70, 75] • Accessibility of the intervention [8–10, 25, 37, 38, 41, 45, 47, 52, 55, 69]	
* Descriptive theme:* 3.4 Characteristics of the local and organizational setting in the primary and target context in terms of the following: * Criteria:* …physical and structural environmental conditions [18, 39, 47, 52, 64, 71] …current existence of synergistic or antagonistic interventions [7–9, 17] …the social/cultural local and/or organizational climate [8–10, 18, 25, 38–40, 52, 64] …the general organizational structure and practice [9, 10, 16, 18, 25, 38, 39, 45, 52] …awareness of the intervention and readiness with regard to pre-existing and durable organizational (including political) will for intervention transfer [9, 18, 40, 46, 52, 60] …decision-makers’/leaders’ positive perception of the intervention and its importance/priority, their skills, status, and latitude for action [7, 9, 17, 18, 25, 37, 38, 40, 52, 60, 69] * Criterion:* …support of decision-makers/leaders and (institutional and/or centralized) management [9, 38, 40, 47, 52, 60, 69] particularly with regard to the following: * Sub-criteria:* • Adaptation of the intervention to the target group [9, 52, 60] • Implementation of the intervention [9, 40, 47, 52, 60] • Providing expertise, supervision, assistance, and help [38, 40, 60] • Sustaining professionals’ motivation for involvement and action [40, 60] * Criterion:* …providers’ (professionals’) perception and support of the intervention particularly with regard to the following: * Sub-criteria:* • Need, utility, priority/importance, and effectiveness [8, 9, 40, 46, 52, 69] • Acceptance/acceptability [7, 9, 25, 38, 40, 52, 55, 60] • Financial, scientific, and/or professional interest [8, 9, 60] • Motivation and engagement [8, 9, 17, 18, 25, 40, 47, 52, 69]	
*Higher-order theme:* 4. Criteria of transfer from the primary to the target context	
* Descriptive theme:* 4.1 Characteristics of communication in the target context in comparison to the primary context in terms of the following: * Criterion:* …overall communication by leaders for the coordination of an intervention particularly with regard to the following: * Sub-criteria:* • Goals, a clear structure, and expectations [40, 52, 55, 60] • Management of data flow [40, 47, 49, 52] • (Program) Meetings [40, 47, 49, 60] • Providing results to stakeholders [16, 40, 47, 49, 52, 60] * Criterion:* …quality of communication in multidisciplinary work and in teams particularly with regard to the following: * Sub-criteria:* • Relation dynamics of stakeholders involved in the process [18, 40, 47, 52, 60] • Defined and clear roles [40, 47, 60, 75] • Skills for working together [18, 40, 60] • Information exchange [18, 40, 60, 75]	
* Descriptive theme:* 4.2 Characteristics of knowledge transfer in the target context in comparison to the primary context in terms of the following: * Criterion:* * …*existence of a ‘knowledge translation’ process for the intervention particularly with regard to the following: * Sub-criteria:* • Support from (trained) specialists [9, 40, 46, 52, 53] • Training of providers/ professionals [8–10, 38, 40, 46, 47, 52, 53, 68, 70] • Knowledge for maintaining the (essential) core elements of the intervention (fidelity) while enabling adaptation to context (flexibility) [17, 27, 46, 47, 53] • Links for knowledge exchange between researchers and stakeholders of the target context [9, 10, 26, 37, 38, 46, 50, 52, 56, 60]	
* Descriptive theme:* 4.3 Characteristics of adoption and implementation in the target context in comparison to the primary context in terms of the following: * Criteria:* …strategies to reach, mobilize, and engage the target population depending on characteristics of the recipients [8–10, 38, 46, 47, 49, 52, 55] …strategies to reach and involve different stakeholders from the beginning [8–10, 18, 40, 46, 47, 50, 52, 55, 60] …identification and addressing of implementation barriers and facilitators [18, 39, 40, 45–47, 52, 53] …strategies of service delivery/intervention delivery [37, 40, 47, 52, 55] …successful pilot-testing of the intervention [37, 47, 52, 53, 55] …possibility of adaptations throughout the intervention’s process, i.e., of the implementation process and/or intervention form by keeping essential (core) elements [9, 39, 40, 52, 56]	
* Descriptive theme:* 4.4 Characteristics of the evaluation in the target context in comparison to the primary context in terms of the following: * Criteria:* …evaluation/study design [8, 16, 26, 37, 45, 47, 53] …kind of assessment of processes and outcomes for measuring intervention success [8, 16, 18, 26, 40, 47, 49, 53] …similarity of determination of effects of the primary and replicated intervention [8, 53, 68, 70] * Criterion:* …continuity and quality of evaluation throughout the transfer process particularly with regard to the following: * Sub-criteria:* • Kind and validity of information of the target context [10, 38, 39] • Validity and reliability of measures [37, 40, 49] • Continuity of monitoring and measuring success throughout the process [9, 37, 38, 47, 49, 52, 53, 56]	
* Descriptive theme:* 4.5 Characteristics of sustainability in the target context in comparison to the primary context in terms of the following: * Criterion:* …sustainability particularly with regard to the following: * Sub-criteria:* • Intervention outcomes [7, 16, 39, 53] • Change of current practice/stability and sustainability of implementation [7, 37, 39, 52, 53] • Key factors in intervention success [17, 37, 47] • Stability of financing [46, 53, 56]	

Descriptive themes and criteria underlie the higher-order themes population, intervention, environment, and transfer, which are numbered from 1 to 4. The descriptive themes are numbered after each higher-order theme to facilitate the attribution to the higher-order theme. All criteria of transferability of health interventions relate to specific descriptive themes. Sub-criteria characterize a criterion in the form of specific aspects relevant to transferability

## Discussion

To improve the health of populations, the concept of EBPH has prevailed [2, 6]. For this purpose, decision-makers need to consider the transferability of effective health interventions from a primary context to their specific target context [7–10]. The aim of this systematic review was to develop a model that can systematically support this decision-making process. This is the first systematic review that has developed a theoretical conceptual model for the assessment of transferability of health interventions by using a thorough, specific interpretative methodology of synthesis. In addition, a novel transferability model was developed that is intended to support the process of determining transferability over time in conjunction with the use of systematized criteria. Several facilitators and barriers of transferability were derived from the literature in order to support the understanding of the criteria. The thematic synthesis revealed four higher-order themes, which build the overarching structure for both models, criteria and facilitators and barriers: the population, the intervention, the environment in both primary and target context, and the transfer of the intervention. Transferability of health interventions depends on criteria underlying these four themes.

An assessment of transferability of health interventions requires information from both the primary and target context. The conceptual PIET-T model leads to the hypothesis that the more both contexts resemble each other against those criteria that determine intervention success, the more likely is the transferability of the intervention. Several authors recommend comparing the primary and target context under consideration of relevant criteria for transferability [8–10, 37–39]. In addition, it is assumed that outcomes are dependent on the population, the intervention, and the environment in a given context and that the underlying characteristics influence one another. This is a well-known assumption with regard to health interventions, which are seen as complex [8, 16, 26, 37, 39–41]. A context is understood as a system with unique characteristics of the population and the environment into which an intervention is introduced. This assumption is consistent with the view of Hawe et al. [42], who take a system perspective and see a complexity in the interactions between the intervention and contextual conditions. Also, Pfadenhauer et al. [43, 44] consider interactions between context and intervention. Further, these authors emphasize the influence of contextual conditions on implementation efforts. In the PIET-T models, all these aspects are specified for transferability of health interventions in addressing criteria of the population, the environment (contextual conditions), the intervention, and the transfer of the intervention. The thematic allocation of the process criteria to the higher-order theme transfer is based on the theoretical distinction between effects of the intervention and influences of the process of transfer on outcomes, which is confirmed by several authors [17, 37, 39].

Thus, the theoretical foundation of the PIET-T models suggests, that information on all four themes is necessary for an assessment of transferability. However, what criteria are relevant may differ with regard to the specific health problem and the compared contexts. Also, the relevance of data gathering in the target context as well as the relevance of detail and validity of information from both contexts may differ between criteria. Therefore, it is recommended to consider the level of transfer, which is a component of the conceptual PIET-T model. For example, the structure of the health care system and inherent services may be of high importance when transferring an intervention from one country to another [17], but may play a subordinate role when the intervention is transferred from one community to another in the same region. Furthermore, Schoenwald and Hoagwood [38] suggest that not all differences found between primary and target context may be of equal relevance for transferability.

The essential result of the thematic synthesis, with regard to the PIET-T process model, is that an initial assessment and evaluation are crucial in determining the transferability of health interventions. It may be helpful to use the initial assessment as a basis to build hypotheses on the conditions under which the intervention will be effective or ineffective in order to capture relevant criteria in the evaluation, because it is not possible to include all potential factors [38, 39]. Thereby, it may be useful to anticipate the desired effects of the outcome by using or gathering the baseline data from the target context before the intervention transfer and to compare the results obtained after the transfer with the baseline data, instead of focusing on the effect (size) of the primary context as a yardstick for success, which is explained in the conceptual model. With this, the evaluation can also be used to check the extent to which the initial assessment was successful. Due to the mutual influence of the population, the intervention and the environment in the target context decision-makers should anticipate relevant relationships between operationalized criteria. Several authors point to the importance of considering effect modification when planning an evaluation of intervention effects [25, 37, 39, 45]. Further, mediating factors may be relevant. For example, the Multisystemic Therapy Transportability Study considered influencing factors by using a mediation model on supervisory, organizational, and interagency factors, clinicians’ intervention fidelity and outcomes for children [38]. Several authors recommend including qualitative approaches in process evaluation, in order to adapt the intervention to population needs, explain outcomes (what and how it works), and build a basis for informing policy and practice [8, 26, 37, 39, 40, 46–50].

Regarding relevant criteria underlying the four themes, the initial assessment may also assist in the systematization to anticipate to what extent intervention fidelity is possible and adaptation is necessary to achieve success. The initial assessment may also help consider the expected extent of change and evolution in the target context and serve to anticipate consequences for the outcome and comparability of results with the primary context. The possibility of adaptations by keeping the primary intervention’s fundamental nature and intervention fidelity is a strong criterion supported by 20 articles, which may become important for implementation preparation and during the implementation process.

Carroll et al. [51] propose to consider content, coverage, frequency, and duration of the intervention regarding planning and evaluating fidelity. Thereby, the analysis of essential core elements of the intervention thought to be responsible for effects may be useful [17, 46, 47, 51–56]. Core elements can be defined by theory, explored by experience in implementing the intervention, or evaluated by a formal component analysis [46, 51]. The analysis of core elements may enable more evolution through flexibility, adaptation, and innovation in order to tailor the intervention to the target context and enhance effective implementation [57–59]. Thus, for the assessment of transferability, the detail of information on the intervention provided by primary evidence is particularly relevant.

The assessment of transferability can be facilitated by collaboration between decision-makers, such as policy-makers, intervention experts, researchers, and stakeholders from the target context. The relationships or links for such a knowledge exchange are a criterion of the descriptive theme knowledge transfer, which is supported by several authors [9, 10, 26, 37, 38, 46, 50, 52, 56, 60]. Enabling the transferability of an intervention may require careful consideration of different views of coordination players as well as skills and knowledge for the assessment of relevant criteria in order to decide on, plan, and realize the intervention transfer. For example, for the initial assessment, researchers may search for and assess the quality of evidence with the use of appropriate instruments. In some cases, an assessment of quality may not be necessary, for example, when high quality guidelines are available. For decision-makers, it may be particularly relevant to judge the usefulness of the intervention for a given health problem. Thus, it may be helpful to first consider and screen criteria of the utility of primary evidence for decision-making, such as the population addressed by the primary intervention, the up-to-dateness of the intervention, the relevance of the results in terms of intervention success, and the availability of sufficient information for application, which serves as a more pragmatic approach.

In this sense, the question on what is the best available evidence for evidence-based public health, from the perspective of transferability, arises. The transfer of evidence to practice and the usefulness of research on different stages, such as the generalizability of results of randomized controlled trials (RCTs) regarding the complexity of health interventions, is a matter of debate, and has been for decades [8, 26, 37, 38, 41, 45, 46, 50, 56, 61–65]. The underlying assumption is a sequential fashion of intervention testing (efficacy research), effectiveness research under real-world conditions and replication/dissemination at a larger scale [26, 45, 61, 65, 66]. For evidence-based public health, several authors call for the importance of relying on a variety of types of evidence [26, 38, 45, 65]. The results of the present thematic synthesis support these authors in that various factors of the primary and target context must be taken into account in order to anticipate the transferability of (complex) health interventions, and that a focus on the evidence level of the study design is important, but not sufficient for the selection and recommendation of primary evidence [16, 17, 25, 37].

Drawing on the results of the thematic synthesis, the conceptual PIET-T model proposes to consider the level of transfer under consideration of the quality and utility of the primary evidence and the planned level of implementation in the target context and offers a flexible approach of thinking with regard to the differing complexity of health interventions. With this, the conceptual model recognizes the facilitating value of research on different stages and levels for transferability [26, 38, 45], for example, by translational research comprising replication or dissemination research in new settings, such as cluster-RCTs for high-level evidence in different communities [16, 17, 37, 47].

### Strengths and limitations

This review has several methodological strengths: systematic rules were developed as an audit trial for searching, selecting, analyzing, and coding data to improve credibility and dependability of the findings. The process of data analysis and the results were discussed and agreed among the authors as well as with a group of experts during a project meeting of the Models of Child Health Appraised (MOCHA) project (TNO Leiden, 23. August 2016) to ensure confirmability. To facilitate transferability of the models and the criteria, detailed information on each criterion is provided in the additional files. A saturation of descriptive themes and criteria was reached in quality ranking level 6, which means that all criteria are supported by at least one article with a high or medium relevance level. Articles of lower relevance levels turned out to valuably enrich examples and verify the criteria. Further, the descriptive themes were supported by many authors independent of the main work field (at least nine articles per descriptive theme).

However, several limitations should be noted: although a comprehensive search strategy was used, relevant articles may have been overlooked, as the review was limited to published articles in English and German, and gray literature was not searched. A major limitation of the synthesis is the heterogeneity of articles and the limited empirical support of criteria. It was shown by a broad orientating search and the systematic search that transferability of health interventions as a concept is rarely described in the literature. It is therefore potentially possible that not all relevant criteria are covered, but the models are open to new criteria or descriptive themes. Further, decision-makers need to choose or operationalize the criteria; therefore, examples for potential factors are provided. Additional instruments to judge criteria may be needed, such as instruments for quality assessment of the evidence or for transferability of health economic evaluations, which go beyond the scope of this review. The identified facilitators and barriers should not be regarded as own criteria, rather, they are intended to deepen the description and understanding of the criteria. Few barriers and facilitators were identified. However, the high support of the descriptive themes and criteria suggests that the higher-order themes population, intervention, environment, and transfer are suitable to explain the concept of transferability and to build the theoretical models for an assessment.

## Conclusions

The results of this systematic review and thematic synthesis show that transferability of health interventions is a complex concept which needs systematic consideration of the primary and target context. The initial assessment and the evaluation are crucial in determining the transferability of health interventions. These aspects point to some important implications for research, policy, and practice: first, to facilitate transferability assessment, researchers should provide a sufficient description of research in terms of the population, the intervention, environmental conditions, processes, and results to enhance the usefulness of primary evidence. Transferability criteria may assist in considering relevant aspects for reporting. Second, research on various stages and levels is relevant to enhance transferability in order to provide a rich body of evidence and systematically investigate factors that influence the transferability of health interventions. Third, decision-makers need systematic and practically relevant knowledge on transferability. This may be supported through more practical tools, useful information about transferability, and close collaboration between research, policy, and practice. The PIET-T models aim to facilitate the assessment of transferability and may serve as a theoretical aid for decision-making, planning, and realizing transfer of health interventions. However, this is the first theoretical work with a conceptual and process model on transferability based on a systematic synthesis of the literature. To what extent this conception is helpful for decision-makers and useful for research and practice must be evaluated. Further research is needed to develop a more practical tool for the models, to evaluate this tool with different target groups and to investigate its usefulness in practice.

## Additional files

Additional file 1: Methodological details. Methodological details for transparency of the systematic review and thematic synthesis. (PDF 275 kb)

Additional file 2: Study characteristics. **Table S1.** Characteristics of the included articles. (PDF 226 kb)

Additional file 3: Guide for an initial assessment and process planning. **Table S2.** Guide for an initial assessment of transferability and the planning of the transfer process. **Table S3.** Overview of all descriptive themes, criteria and sub-criteria. (PDF 222 kb)

Additional file 4: Description of the results of the descriptive themes. Descriptive text of the qualitative results of the thematic synthesis belonging to Table 2. (PDF 272 kb)

Additional file 5: Detailed table of criteria with examples. **Table S4.** Comprehensive table with descriptive themes and criteria. (PDF 282 kb)

Additional file 6: Detailed table of barriers and facilitators. **Table S5.** Comprehensive information on barriers and facilitators. (PDF 166 kb)

## Abbreviations

ACT: Assertive Community Treatment
EBPH: Evidence-Based Public Health
ENTREQ: Enhancing Transparency in Reporting the Synthesis of Qualitative Research Statement
MESH: Medical Subject Headings
MOCHA: Models of Child Health Appraised
PICOS: Population, Intervention, Comparison, Outcome, Study design
PIET-T: Population-Intervention-Environment-Transfer Model of Transferability
SRA-DM: Systematic Review Assistant-Deduplication Module
