# Supplementary material for: Criteria for evaluating transferability of health interventions: a systematic review and thematic synthesis
Source: Implement Sci. 2018 Jun 26;13:88. doi: 10.1186/s13012-018-0751-8 (PMC6019740; doi:10.1186/s13012-018-0751-8)
Supplement: Supplementary file 1 — Methodological details. Methodological details for transparency of the systematic review and thematic synthesis. (PDF 275 kb) [file 13012_2018_751_MOESM1_ESM.pdf]

Additional file 1: Methodological details

Search strategy

Thomas and Harden [29] propose to use a systematic search strategy for the identification of relevant publications. Therefore, the search was pre-planned. The PICOS-strategy (population, intervention, comparison, outcome, study design) was used for the development of the search strategy and selection of articles. A specification of the population and a comparison was not applicable to the objectives of research/research questions. Key words for health interventions and the outcome were retrieved from the Medical Subject Headings (MESH) database in PubMed and from the literature on transferability, such as health care, health service, public health, health policy, program, intervention and transferability or transferable. In order to find the most appropriate search algorithm, many combinations of search terms were piloted in Google Scholar and the databases PubMed, Embase, CINAHL, PsycINFO and Web of Science, as is recommended by Jackson and Waters [32] for systematic reviews relating to health interventions. Although the development of the search strategy focused on being comprehensive and revealing a wide range of articles, it was not the objective to find every available article as is usually the goal for quantitative systematic reviews. According to Thomas and Harden [29] the range and richness of descriptions of a concept found in the articles is more relevant for a thematic synthesis than the number of articles found on the topic. For the development of the search algorithm, the emphasis was given to the desired content of the publications based on the definition of transferability. Therefore, the inclusion of related terms with different meanings, such as external validity, generalizability and applicability was avoided. Instead, search terms relating to health interventions were chosen very broadly in order to find articles on transferability in different work fields. Furthermore, the search was not restricted to a study design or a special time frame, because the piloting revealed a lack of research which addressed criteria of transferability. The following final combination of search terms was entered in the databases PubMed, Embase, CINAHL and PsycINFO on 6 June 2016: ("transferability") AND ("health" OR "health care" OR "policy" OR "prevention" OR "service" OR "intervention" OR "program" OR "programme" OR "implementation"). These databases and search terms provided comprehensive as well as precise search results. In the databases CINAHL and PsycINFO a further restriction was made to journal articles.

In addition, all references of the articles included in the thematic synthesis were screened, which is recommended to identify articles that would otherwise be missed through a process of snowballing [33]. A backward snowballing approach was used, with the finally included articles of the database search as a basis for the determination of the start set for snowballing. That means, the reference lists of these papers were worked through to identify potential articles for the screening process. The start set included all references that were related to the topic of transferability of health interventions regardless of eligibility criteria. Subsequently, all duplicates were documented and removed. The further screening of titles, abstracts and full-texts was the same as the screening process of the database search by using the defined eligibility criteria. The flowchart of the study selection (Fig. 1) summarizes the results of the screening process of both the database search and the snowball search.

Eligibility criteria

To be eligible for the thematic synthesis, articles had to provide a description of transferability by using the exact term or a synonymous description which is in line with the following definition: transferability refers to the extent to which the outcomes of a successful health intervention evaluated in a primary context can be achieved in a target context. An article with a synonymous description was eligible when another word was used instead of transferability, but the description had the same meaning as the definition.

Examples for descriptions of transferability in line with the given definition:

"This raises the question of the transferability of these interventions, i.e., the extent to which the result of one intervention in a given context can be achieved in another context [9]."

"An intervention's transferability is defined as the extent to which its effects in a given setting can be observed in another setting [60]."

"So, a key question raised by these interventions has to do with their transferability, which has been defined as the extent to which the measured effectiveness of an applicable intervention could be achieved in another setting [8]."

"Wang and colleagues suggest a new definition of these terms where 'applicability' indicates whether the intervention could practically be implemented in the new setting, therefore focussing on the process of implementation, whilst 'transferability' indicates whether the intervention can be expected to have the same effect in the new setting, therefore focussing on the outcome of the intervention [39]."

"…and transferability as the extent to which the measured effectiveness of an applicable intervention could be achieved in another setting [10]."

Examples for synonymous descriptions of transferability in line with the definition:

"Can effective programs be replicated? Policy makers and advocates often assume that a social program that is effective in one setting will automatically produce the same results in other settings. Policy makers should not make this assumption (…) assuming that a program that works in one location or with one population will automatically work in other situations [16]."

"Assuming that an intervention conducted by a service organization produces the same behavioral effects as that same intervention produced for participants in the research trial, the organization is implementing an intervention whose effectiveness has been established. The assumption that service organization programs will achieve the same outcome as those in the trial is most justified when the interventions are also the same [46]."

"Transportability research examines the movement of efficacious interventions to usual-care settings (…) However, if the experimental seed grew to bear corn only in a test tube, efforts to disseminate it to the agriculture industry would be premature. Instead, evidence that the seed will grow in soil and under typical growing conditions would have to be generated (…) The resulting dilemma is that treatments validated in efficacy studies may not be effective when implemented under conditions facing most community practitioners [38]."

Quality ranking

In order to classify criteria in terms of their relevance for the assessment of transferability of health interventions, a quality ranking was developed based on the quality assessment strategy for criteria on external validity provided by Dyrvig et al. [35]. The first condition for the quality ranking was the precision and richness of the description of criteria for transferability of health interventions. The second condition was the extent of support of the transferability criteria based on the methodology of the article. Dyrvig et al. [35] describe four different support categories. The first category builds empirical support. In this review, empirical support was documented when an empirical investigation of transferability was carried out or transferability criteria were developed or supported by an empirical investigation. The second category is literature support. Literature support was determined when criteria were reported on the basis of a literature review, i.e. when criteria were supported by references. Consensus support, the third category, was identified when criteria were supported by reaching consensus in a group of experts. The fourth category was recorded when no defined background, that is, no methodological justification for criteria was found in the article [35].

Based on these conditions for quality, the ranking system was developed and applied by one author (TS), reaching from 1 to 10. An article with the highest ranking of 1 showed an empirical investigation of transferability with a sound description of the concept of transferability and detailed, explicit description of transferability criteria. An article with the lowest ranking of 10 provided a description of transferability by a synonym (as understood in the definition) and explicit or implicit mentioning of criteria, but no defined background:

1. Empirical investigation of transferability with a sound description of the concept of transferability and detailed, explicit description of transferability criteria
2. Empirical investigation of transferability with an explicit description of transferability by using the term and an explicit and implicit detailed description of transferability criteria
3. Empirical investigation with a description of transferability by using the term and an explicit criteria list or questions on transferability with few criteria or no detailed description of criteria, e.g. in a tool
4. Reviewing literature with a description of transferability by using the term and an explicit description of criteria or questions on transferability, e.g. in a special part, tool, list, synthesis
5. Empirical investigation with a description of transferability by using the term and explicit or implicit description of criteria, but not separately for transferability, e.g. for adaptation/implementation and transferability
6. Reviewing or discussing literature with a description of transferability by using the term and explicit description of criteria, but not separately for transferability, e.g. for applicability and transferability
7. Reviewing or discussing literature with a clear description of transferability by using the term and an implicit description of criteria
8. Reviewing or discussing literature by using a synonymous description of transferability (which can be understood as described in the definition of transferability) and explicit or implicit description of criteria
9. Article with description of transferability by using the term and explicit or implicit description of transferability criteria, but without defined background
10. Article with description of transferability by a synonym (as understood in the definition) and explicit or implicit mentioning of criteria, but without defined background

This ranking system determined the order of analysis for the thematic synthesis. The order of the analysis of the articles and the resulting synthesis are therefore based on the quality of the information obtained about transferability of health interventions and are based on methodological and theoretical considerations to achieve accuracy and credibility as much as possible.

In order to facilitate the reader's access to information, three relevance levels were determined from the ranking, which indicate whether the article has high, medium or low relevance for the analysis:
Ranking of 1-4: high relevance
Ranking of 5-7: medium relevance
Ranking of 8-10: low relevance
The quality ranking for each article can be found in Additional file 2: Study characteristics. Table S1.

Data analysis
As a basis for the thematic synthesis the following information was extracted from the studies: Authors, year of publication, title, the type of transferability of health interventions relating to the main field (e.g. health promotion, prevention, health technology), and the support category. Further, the description of transferability was documented for each article. The thematic synthesis was structured according to the quality ranking described above. Because the articles had different methodological foundations and were included as qualitative material in dependence on the background provided for transferability criteria, no limitation of the material was made to a results section. Indeed, such a limitation would have limited the precision and richness of description of the concept of transferability, which is rarely empirically addressed in the literature.

Stages 1 and 2
The analysis was conducted in three stages as recommended by Thomas and Harden [29]. Stage 1 and 2 include a free line-by-line coding of text and the organization of the codes into related areas for the construction of descriptive themes [29]. Starting with the articles of the highest quality, the original texts were entered into an Excel file and the material was worked through line-by-line for coding. Initial codes were created, which represented criteria for transferability of health interventions. Thereby, rules were established to improve credibility of the analysis. The minimum segments of text for extraction of criteria were at the word level. For barriers and facilitators they were at the sentence level, because the word level seemed to narrow and contained the risk of losing meaning of the text [36]. Further, codes were first extracted from articles with the highest quality to take over and remain as close as possible to already existing (empirically investigated) criteria.

The resulting criteria were verified through the same criteria derived from further articles (translation between articles). Where an original text passage, sentence or word could not be assigned unambiguously to transferability, criteria were only confirmed if they were reported by other authors to secure the correct assignment to transferability. Consistency of interpretation/assignment and the need for new levels of coding of criteria was checked continuously [29].

For some of the criteria, sub-criteria emerged. All resulting criteria were grouped into a hierarchical structure for the development of descriptive themes (stage 2; [29]). This structuring was performed according to the codes based on existing structures of high quality articles and on theoretical considerations emerging from the texts and the process of synthesis. For verification of the criteria and descriptive themes by the original articles all steps were documented in detail with references. All initial material resulting from each step was rechecked to ensure consistent allocation against the themes.
For the identification of facilitators and barriers, the same procedure was used. Original texts were also read line-by-line. Sentences were identified as a facilitator or barrier, when negative or positive consequences for transferability were described. With this, the extraction of facilitators and barriers aimed at providing a deeper understanding of the criteria. In addition, steps of an assessment of transferability emerged from the data. These were extracted and grouped in the same way as described before.

Some examples for first line-by-line coding of text:

Original text (here only extract)	Examples for (first) line-by-line coding
"Factors influencing transferability (…). The following factors were highlighted: whether the professionals followed the experimental protocol; (…) and, possibly, the modifications required for the new context [8]."	Whether the professionals followed the experimental protocol (= intervention fidelity) and modifications required for the new context.
A ''Key functions/implementation/context'' model was developed and tested to describe and analyze the intervention, its implementation, and the context within which it is implemented, to enhance its potential transferability. (…). Key functions (…) are defined as essential processes underlying concrete implemented activities, i.e., the functions those activities are meant to have in order to achieve the intervention's objectives. (…) the key functions and the form of an intervention as an analytical lens (…) focusing on its potentially transferable elements, while taking implementation context into account [56]."	Identification of transferable key elements of the primary intervention under consideration of<br>- the intervention's key functions (essential processes underlying activities to reach the objective of the intervention)<br>- the specific form of the intervention<br>- the implementation context.
"Intervention characteristics focused on details about what the intervention comprised (…) and the extent to which the intervention was delivered as intended in the study (…). The adaptability of the intervention covered criteria that examine the extent to which the intervention was adapted or modified over time or after the evaluation, or whether the degree of modification differed between settings. It also includes criteria that consider the inherent flexibility of the intervention, or its potential for adaptation [7]."	The extent to which the intervention was delivered as intended; adaptability, extent to which the intervention was adapted or modified over time or after the evaluation, degree of modification, flexibility of the intervention, or its potential for adaptation.
"Balancing standardization and flexibility (…). Establishing standards through use of an Implementation Guide was important to ensure the integrity of the intervention, and program monitoring provided learning about the balance of consistency and flexibility needed to integrate CHAP into diverse communities with varying resources [47]."	Balancing standardization and flexibility to ensure integrity of the intervention and contextual integration.

"The REP intervention package conveys core elements – the critical features of the design and intent of the intervention that are thought to be responsible for the intervention's effectiveness – are essential for maintaining fidelity. Still, while core elements are standardized, the mechanisms by which they can be operationalized may vary across organizations [53]."	Determination and description of the intervention's core elements (thought to be responsible for effectiveness) for fidelity and varying modification of mechanisms.

The further coding process through constant comparison and translation between all included articles resulted in the following criterion:
Possibility of adaptations/modifications by keeping the primary intervention's fundamental nature and intervention fidelity through considering the sub-criteria:
• *identification of transferable core elements/key functions*
• *identification of elements which are not transferable or need modification*
• *modification/adaptation of the specific form of the intervention*
This criterion was grouped together with other thematically similar criteria and sub-criteria (e.g. *conception of the intervention*) for the building of the descriptive theme *characteristics of the intervention content in the primary and target context*.

Stage 3
Stage three is the step of going beyond the findings of the included articles, in which analytical or higher-order themes and their relationships, additional understandings and hypotheses are developed. Higher-order themes are emerging overarching themes which describe the subject of inquiry by using general insights from the synthesis [29, 36]. Stage three was used to build higher-order themes out of the descriptive themes and to conceptually model their relationships and the mechanism which underlies transferability of health interventions in order to create a meaningful whole out of the findings and to provide a theoretical conception of transferability [36]. Furthermore, a process model was developed based on the thematic steps for determining transferability and was brought together with the identified criteria of transferability of health interventions.

Example for the building of higher-order themes and conceptual modelling:

The descriptive themes that emerged from the inductive analysis were compared and grouped to develop higher-order themes. In addition, key information was gained from the original material as a basis for interpretation of the mechanism of transferability, which described relationships between themes and important aspects on the concept of transferability. The modelling was conducted in an analytical, cyclic process by analyzing and interpreting key findings from all 3 stages of the analysis.

Example of a higher-order theme (intervention):
- *Characteristics of the evidence base for comparison of primary and target context*
- *Characteristics of the intervention content in the primary and target context*

These descriptive themes with their underlying criteria refer to conditions of the *intervention*.

Examples of key information:

"To assess the transferability of evidence about an intervention information is needed on (a) the intervention itself (b) the (…) context, and (c) interactions between the intervention and its context. (…) Important contextual characteristics for a public health intervention might include factors in the (…) environment and (…) of the population [37]."

"For each treatment, the magnitude of similarity or difference between the conditions that characterized the validation studies and those that characterize real-world service settings and systems can be estimated. Data on similarities and differences are not yet available for many dimensions, precisely because we have not conducted research in ways that assess them [38]."

"Public health intervention effectiveness is influenced by characteristics of the intervention, its implementation, the target setting or population, and prevailing social, economic and policy contexts. The interactions must be understood to determine whether evidence from one setting is relevant or valuable for policy and practice decisions elsewhere [26]."

"Transferability depends on implementation conditions and on the interaction between the intervention and the context into which it is inserted [60]."

"If an intervention's key functions are transferred to a new setting, local actors will decide on the form of the replicated intervention according to the specificities of and the knowledge they have about the features of their context. In this way, the interaction between the intervention and its context can have a better chance of producing results [56]."

"Interventions cannot be understood independently of the social and cultural contexts in which they are developed and applied [41]."

"information is needed on: the context (…); the design and components of the intervention; and any potential interactions between the intervention and its context. (…) clearly distinguish between the successful transfer of intervention processes and the replication of intervention outcomes [39]."

"Programs almost never proceed as planned. Instead, they, and the assumptions that guide them and shape their actions, usually change over time as the programs progress. (…) Ideally, the evolution of the program and the lessons learned are captured (…) the need to both describe the program and understand how it works remains just as pressing, particularly when apparently successful programs are rolled out in new contexts. (…) Recognizing the importance of understanding how and why interventions work has come more slowly, but without a clear account of what an interventional program comprises, how its activities are linked to its outcomes, and how context and program interact, its operation remains a black box [40]."
