# Supplementary material for: Criteria for evaluating transferability of health interventions: a systematic review and thematic synthesis
Source: Implement Sci. 2018 Jun 26;13:88. doi: 10.1186/s13012-018-0751-8 (PMC6019740; doi:10.1186/s13012-018-0751-8)
Supplement: Supplementary file 2 — Study characteristics. Table S1. Characteristics of the included articles. (PDF 226 kb) [file 13012_2018_751_MOESM2_ESM.pdf]

Additional file 2: Study characteristics

Table S1 Characteristics of included articles

**Author and year**	**Title**	**Transferability type**	**Extracted criteria based on article type (support category)**	**Quality ranking for analysis**	**Level of relevance**
Ashton (2015)	Implementing integrated models of care: the importance of the macro-level context	Health care	Literature support (discussing literature)	**7**	**medium relevance**
Buffet et al. (2007)	Can I use this evidence in my program decision? Assessing applicability and transferability of evidence	Public health programs (tool for programs)/interventions	No defined background (assessment tool)	**9**	**low relevance**
Burchett et al. (2011)	How do we know when research from one setting can be useful in another? A review of external validity, applicability and transferability frameworks	Findings/evidence in general	Literature support (reviewing literature/criteria)	**6**	**medium relevance**
Cambon et al. (2012)	Transferability of interventions in health education: a review	Health promotion interventions, programs	Literature support (reviewing literature/criteria)	**4**	**high relevance**
Cambon et al. (2013)	A tool to analyze the transferability of health promotion interventions	Health promotion interventions, programs	Empirical support (concept mapping and expert surveys)	**1**	**high relevance**

Carter et al. (2009)	Implementing a standardized community-based cardiovascular risk assessment program in 20 Ontario communities	Health prevention program/ Cardiovascular risk assessment program CHAP	Empirical support (community-level, clustered, randomized, controlled trial; in addition, semi-structured telephone interviews were conducted with local coordinators or coordinator teams in 20 communities)	**2**	**high relevance**
Chase et al. (2009)	Development of a toolkit and glossary to aid in the adaptation of health technology assessment (HTA) reports for use in different contexts	Health technology	Empirical support (and literature support and consensus support) (literature searching, survey, Delphi survey, meetings, drawing on expertise/ experience of partnership, review, quality assurance testing)	**3**	**high relevance**
Cuijpers et al. (2005)	Adapting and disseminating effective public health interventions in another country: towards a systematic approach	Public Health interventions, programs	No defined background	**10**	**low relevance**
Dixon-Woods et al. (2011)	Explaining Michigan: developing an Ex Post Theory of a Quality Improvement Program	Health programs; Michigan Intensive Care Unit (ICU) project, health care	Empirical support (eighteen-month prospective cohort study of the Michigan Keystone Project on reduction of central venous catheter bloodstream infections (CVC-BSIs) in more than one hundred participating intensive care units (ICUs) in the state of Michigan)	**5**	**medium relevance**
Feldstein and Glasgow (2008)	A Practical, Robust Implementation and Sustainability Model (PRISM) for integrating research findings into practice	Findings/evidence in general; with a focus on health care	Literature support (reviewing literature/methodological paper, development of a model)	**8**	**low relevance**

Glasgow et al. (2003)	Why don't we see more translation of health promotion research to practice? Rethinking the efficacy-to-effectiveness transition	Health promotion interventions, programs	Literature support (discussing literature/ methodological paper, description of the RE-AIM evaluation framework)	**8**	**low relevance**
Glasgow et al. (1999)	Evaluating the public health impact of health promotion interventions: The RE-AIM framework	Health promotion interventions, programs	Literature support (discussing literature/ methodological paper, description of the RE-AIM evaluation framework)	**8**	**low relevance**
Granstrøm Ekeland and Grøttland (2015)	Assessment of MAST in European patient-centered telemedicine pilots	Health technology (telemedicine application)	Empirical support (mixed methods approach with questionnaires on the Model for ASsessment of Telemedicine Applications (MAST))	**3**	**high relevance**
Guegan et al. (2011) (EUnetHTA)	EUnetHTA HTA Adaptation Toolkit & Glossary: Adapting existing HTAs from one country into other settings	Health technology	Empirical support (and literature support and consensus support) (literature searching, survey, Delphi survey, meetings, individual members' commentary work, review process and applicability testing)	**3**	**high relevance**
Heller et al. (2008)	Critical appraisal for public health: a new checklist	Public health interventions and other study types in public health, not specified	Literature support (and consensus support) (reviewing criteria and piloting with professionals, research staff or students)	**6**	**medium relevance**
Kelly et al., (2000)	Transfer of research-based HIV prevention interventions to community service providers: fidelity and adaptation	Public health intervention, specified on HIV prevention	Literature support (reviewing literature/methodological paper particularly based on evaluation studies of the Replicating Effective Programs (REP) project)	**8**	**low relevance**

Kidholm et al. (2012)	A Model for Assessment of Telemedicine Applications: MAST	Health technology (telemedicine application)	Literature support (and consensus support) (systematic literature review and workshops)	**4**	**high relevance**
Kilbourne et al. (2007)	Implementing evidence-based interventions in health care: application of the replicating effective programs framework	Health care interventions, programs	Empirical support (and literature support) (Replicating Effective Programs framework (REP) builds on a systematic literature review and community input and is evaluated through a randomized controlled trial)	**2**	**high relevance**
Muhlhausen (2012)	Evaluating Federal Social Programs: finding out what works and what does not	Programs/ interventions, including public health	Literature support (discussing literature)	**8**	**low relevance**
Pawson (2003)	Nothing as practical as a good theory	Findings/evidence in general; examples of programs	Literature support (discussing literature)	**7**	**medium relevance**
Pearson et al. (2011)	Generalizing applied qualitative research on harm reduction: the example of a public injecting typology	Findings/evidence of qualitative research	Empirical support (and literature support/methodological paper) (ethnographic approach with semi-structured interviews, participant and direct observation)	**3**	**high relevance**
Perleth (2009)	Assessment of the generalizability of clinical trial results in the Federal Joint Committee	Findings/evidence in general	Literature support (discussing literature)	**6**	**medium relevance**
Rychetnik et al. (2012)	Translating research for evidence-based public health: key concepts and future directions	Public health interventions, not specified	Literature support (reviewing literature, methodological paper)	**4**	**high relevance**

Rychetnik et al. (2002)	Criteria for evaluating evidence on public health interventions	Public health interventions, programs	Literature support (reviewing and discussing literature, methodological paper)	**4**	**high relevance**
Saurman et al. (2014)	A transferable telepsychiatry model for improving access to emergency mental health care	Health care service: Mental Health Emergency Care-Rural Access Program (MHEC)	Empirical support (qualitative ethnographic observational study)	**5**	**medium relevance**
Schoenwald and Hoagwood (2001)	Effectiveness, transportability, and dissemination of interventions: what matters when?	Evidence-based interventions in health care (mental health)	Literature support (discussing literature)	**8**	**low relevance**
Schreyögg (2004)	Seeking best practices: a conceptual framework for planning and improving evidence-based practices	Findings/evidence in general, (best) practice/evidence of programs	Literature support (discussing literature)	**7**	**medium relevance**
Spencer et al. (2013)	Justice in health care systems from an economic perspective	Health care elements	Literature support (and consensus support) (review of the literature and expert input)	**4**	**high relevance**
Tham et al. (2011)	Study protocol: evaluating the impact of a rural Australian primary health care service on rural health	Health service/primary health care	Literature support (literature-based study protocol with an evaluation framework of a planned comprehensive longitudinal evaluation of a successful primary health care service located in a small rural Australian community)	**7**	**medium relevance**

Trompette et al. (2014)	Stakeholders' perceptions of transferability criteria for health promotion interventions: a case study	Health promotion/ prevention interventions	Empirical support (case study with stakeholder interviews)	**1**	**high relevance**
Van Royen et al. (2014)	Patient-centred interprofessional collaboration in primary care: challenges for clinical, educational and health services research. An EGPRN keynote paper	Findings/evidence in general, focus on primary health care	Literature support (discussing literature)	**7**	**medium relevance**
Villeval et al. (2016)	Enabling the transferability of complex interventions: exploring the combination of an intervention's key functions and implementation	Health promotion	Empirical support (qualitative research with observation and interviews; knowledge exchange process)	**2**	**high relevance**
Wang et al. (2005)	Applicability and transferability of interventions in evidence-based public health	Public health interventions, programs	Literature support (review of the literature and methodological paper; discussion based on a systematic search on the terms applicability and transferability)	**4**	**high relevance**
Watts et al. (2011)	The influence of environmental factors on the generalizability of public health research evidence: physical activity as a worked example	Public health interventions, programs; (physical activity); health promotion	Literature support (brief review, and methodological paper, discussing literature)	**7**	**medium relevance**
Wegscheider (2009)	Transferability of study results to health care practice: contribution of different qualitative and quantitative research approaches	Findings/evidence in general, mainly for medical treatment	No defined background (opinion paper)	**9**	**low relevance**

Weinmann et al. (2012)	Team-based community psychiatry: importance of context factors and transfer-ability of evidence from studies	Health care service: transferability of re-sults of trials evalu-ating community-based mental health services	Literature support (discussing lit-erature)	**7**	**medium relevance**
Whitley et al. (2011)	Evidence-based medicine: challenges and opportunities in a diverse society	Evidence-based in-terventions in health care (medicine)	Literature support (discussing lit-erature)	**6**	**medium relevance**

Legend

Quality ranking of relevance for the analysis:
1. Empirical investigation of transferability with a sound description of the concept of transferability and detailed, explicit description of transferability criteria
2. Empirical investigation of transferability with an explicit description of transferability by using the term and an explicit and implicit detailed descrip-tion of transferability criteria
3. Empirical investigation with a description of transferability by using the term and an explicit criteria list or questions on transferability with few criteria or no detailed description of criteria, e.g. in a tool
4. Reviewing literature with a description of transferability by using the term and an explicit description of criteria or questions on transferability, e.g. in a special part, tool, list, synthesis
5. Empirical investigation with a description of transferability by using the term and explicit or implicit description of criteria, but not separately for transferability, e.g. for adaptation/implementation and transferability
6. Reviewing or discussing literature with a description of transferability by using the term and explicit description of criteria, but not separately for transferability, e.g. for applicability and transferability
7. Reviewing or discussing literature with a clear description of transferability by using the term and an implicit description of criteria
8. Reviewing or discussing literature by using a synonymous description of transferability (which can be understood as described in the definition of transferability) and explicit or implicit description of criteria
9. Article with description of transferability by using the term and explicit or implicit description of transferability criteria, but without defined back-ground
10. Article with description of transferability by a synonym (as understood in the definition) and explicit or implicit mentioning of criteria, but without defined background

Relevance categories according to ranking of the articles:
- 1-4: high relevance
- 5-7: medium relevance
- 8-10: low relevance

References of included articles

Ashton, T. (2015). Implementing integrated models of care: the importance of the macro-level context. *Int J Integr Care, 15*, e019.

Buffet, C., Ciliska, D., & Thomas, H. (2007). *Can I Use This Evidence in my Program Decision? Assessing Applicability and Transferability of Evidence*. Hamilton, ON L8S 1G5: National Collaborating Centre for Methods and Tools.

Burchett, H., Umoquit, M., & Dobrow, M. (2011). How do we know when research from one setting can be useful in another? A review of external validity, applicability and transferability frameworks. *J Health Serv Res Policy, 16*(4), 238-244.

Cambon, L., Minary, L., Ridde, V., & Alla, F. (2012). Transferability of interventions in health education: a review. *BMC Public Health, 12*, 497.

Cambon, L., Minary, L., Ridde, V., & Alla, F. (2013). A tool to analyze the transferability of health promotion interventions. *BMC Public Health, 13*, 1184.

Carter, M., Karwalajtys, T., Chambers, L., Kaczorowski, J., Dolovich, L., Gierman, T., . . . Laryea, S. (2009). Implementing a standardized community-based cardiovascular risk assessment program in 20 Ontario communities. *Health Promot Int, 24*(4), 325-333.

Chase, D., Rosten, C., Turner, S., Hicks, N., & Milne, R. (2009). Development of a toolkit and glossary to aid in the adaptation of health technology assessment (HTA) reports for use in different contexts. *Health Technol Assess, 13*(37), 1-142.

Cuijpers, P., Graaf, I., & Bohlmeijer, E. (2005). Adapting and disseminating effective public health interventions in another country: towards a systematic approach. *Eur J Public Health, 15*(2), 166–169.

Dixon-Woods, M., Bosk, C. L., Aveling, E. L., Goeschel, C. A., & Pronovost, P. J. (2011). Explaining Michigan: Developing an ex post theory of a quality improvement program. *Milbank Q, 89*(2), 167–205.

Feldstein, A. C., & Glasgow, R. E. (2008). A Practical, Robust Implementation and Sustainability Model (PRISM) for Integrating Research Findings into Practice. *Jt Comm J Qual Patient Saf, 34*(4), 228–243.

Glasgow, R. E., Lichtenstein, E., & Marcus, A. C. (2003). Why don't we see more translation of health promotion research to practice? Rethinking the efficacy-to-effectiveness transition. *Am J Public Health, 93*(8), 1261–1267.

Glasgow, R., Vogt, T., & Boles, S. (1999). Evaluating the public health impact of health promotion interventions: the RE-AIM framework. *Am J Public Health, 89*(9), 1322–1327.

Granstrøm Ekeland, A. G., & Grottland, A. (2015). Assessment of MAST in European patient-centered telemedicine pilots *Int J Technol Assess Health Care, 31*(5), 304-311.

Guegan, E., Milne, R., Pordage, A., Chase, D., Hicks, N., Bunce, H., . . . Payne, L. (2011). EUnetHTA HTA Adaptation toolkit Workpackage 5. Retrieved from http://www.eunethta.eu/outputs/eunethta-hta-adaptation-toolkit

Heller, R. F., Verma, A., Gemmell, I., Harrison, R., Hart, J., & Edwards, R. (2008). Critical appraisal for public health: a new checklist. *Public Health, 122*(1), 92-98.

Kelly, J. A., Heckman, T. G., Stevenson, L. Y., Williams, P. N., Ertl, T., Hays, R. B., . . . Neumann, M. S. (2000). Transfer of research-based HIV prevention interventions to community service providers: fidelity and adaptation. *AIDS Educ Prev, 12*(5 Suppl), 87–98.

Kidholm, K., Ekeland, A. G., Jensen, L. K., Rasmussen, J., Pedersen, C. D., Bowes, A., . . . Bech, M. (2012). A model for assessment of telemedicine applications: mast. *Int J Technol Assess Health Care, 28*(1), 44–51.

Kilbourne, A. M., Neumann, M. S., Pincus, H. A., Bauer, M. S., & Stall, R. (2007). Implementing evidence-based interventions in health care: application of the replicating effective programs framework. *Implement Sci, 2*, 42.

Muhlhausen, D. B. (2012). Evaluating Federal Social Programs: finding out what works and what does not. *Res Soc Work Pract, 22*(1), 100-107.

Pawson, R. (2003). Nothing as practical as a good theory. *Evaluation (Lond), 9*(4), 471–490.

Pearson, M., Parkin, S., & Coomber, R. (2011). Generalizing applied qualitative research on harm reduction: the example of a public injecting typology. *Contemp Drug Probl, 38*(1), 61–91.

Perleth, M. (2009). Assessment of the generalisability of clinical trial results in the Federal Joint Committee. [German]. *Z Evid Fortbild Qual Gesundhwes, 103*(6), 412-414.

Rychetnik, L., Bauman, A., Laws, R., King, L., Rissel, C., Nutbeam, D., . . . Caterson, I. (2012). Translating research for evidence-based public health: key concepts and future directions. *J Epidemiol Community Health, 66*(12), 1187–1192.

Rychetnik, L., Frommer, M., Hawe, P., & Shiell, A. (2002). Criteria for evaluating evidence on public health interventions. *J Epidemiol Community Health, 56*(2), 119-127.

Saurman, E., Johnston, J., Hindman, J., Kirby, S., & Lyle, D. (2014). A transferable telepsychiatry model for improving access to emergency mental health care. *J Telemed Telecare, 20*(7), 391–399.

Schoenwald, S. K., & Hoagwood, K. (2001). Effectiveness, transportability, and dissemination of interventions: what matters when? *Psychiatr Serv, 52*(9), 1190–1197.

Schreyogg, J. (2004). Justice in health care systems from an economic perspective. *Gesundheitswesen, 66*(1), 7-14.

Spencer, L. M., Schooley, M. W., Anderson, L. A., Kochtitzky, C. S., DeGroff, A. S., Devlin, H. M., & Mercer, S. L. (2013). Seeking best practices: a conceptual framework for planning and improving evidence-based practices. *Prev Chronic Dis, 10*, E207.

Tham, R., Humphreys, J. S., Kinsman, L., Buykx, P., Asaid, A., & Tuohey, K. (2011). Study protocol: evaluating the impact of a rural Australian primary health care service on rural health. *BMC Health Serv Res, 11*, 52.

Trompette, J., Kivits, J., Minary, L., Cambon, L., & Alla, F. (2014). Stakeholders' perceptions of transferability criteria for health promotion interventions: a case study. *BMC Public Health, 14*, 1134.

Van Royen, P., Rees, C. E., & Groenewegen, P. (2014). Patient-centred interprofessional collaboration in primary care: challenges for clinical, educational and health services research. An EGPRN keynote paper. *Eur J Gen Pract, 20*(4), 327-332.

Villeval, M., Bidault, E., Shoveller, J., Alias, F., Basson, J.-C., Frasse, C., . . . Lang, T. (2016). Enabling the transferability of complex interventions: exploring the combination of an intervention’s key functions and implementation. *Int J Public Health*.

Wang, S., Moss, J. R., & Hiller, J. E. (2005). Applicability and transferability of interventions in evidence-based public health. *Health Promot Int, 21*(1), 76–83.

Watts, P., Phillips, G., Petticrew, M., Harden, A., & Renton, A. (2011). The influence of environmental factors on the generalisability of public health research evidence: physical activity as a worked example. *Int J Behav Nutr Phys Act, 8* (128).

Wegscheider, K. (2009). Transferability of study results to health care practice: contribution of different qualitative and quantitative research approaches [German]. *Z Evid Fortbild Qual Gesundhwes, 103*(6), 381-387.

Weinmann, S., Gühne, U., Kösters, M., Gaebel, W., & Becker, T. (2012). Team-based community psychiatry: importance of context factors and transferability of evidence from studies [German]. *Nervenarzt, 83*(7), 825–831.

Whitley, R., Rousseau, C., Carpenter-Song, E., & Kirmayer, L. J. (2011). Evidence-based medicine: Opportunities and challenges in a diverse society. *Can J Psychiatry, 56*(9), 514–522.
