# Supplementary material for: Criteria for evaluating transferability of health interventions: a systematic review and thematic synthesis
Source: Implement Sci. 2018 Jun 26;13:88. doi: 10.1186/s13012-018-0751-8 (PMC6019740; doi:10.1186/s13012-018-0751-8)
Supplement: Supplementary file 3 — Guide for an initial assessment and process planning. Table S2. Guide for an initial assessment of transferability and the planning of the transfer process. Table S3. Overview of all descriptive themes, criteria and sub-criteria. (PDF 222 kb) [file 13012_2018_751_MOESM3_ESM.pdf]

Additional file 3: Guide for an initial assessment and process planning

Table S2 Guide for an initial assessment of transferability and the planning of the transfer process

**Explanation and order of criteria use**	**Guiding questions for criteria use**
The assessment of the evidence base (utility and quality of primary evidence) provides a basis for further decisions on whether the intervention is appropriate to influence the health problem of the target population *(criteria of the intervention).*	Does the primary evidence address the health problem of the target population?<br>How useful is the primary evidence for the target context?<br>How good is the quality of the primary evidence?
Criteria about the intervention content provide information on whether the conception of the intervention is suitable for the target population and the environment *(criteria of the intervention).*	How is the intervention conceptualized?<br>Does the conception of the intervention fit the target context?
The comparison of relevant criteria of the population and the environment between primary and target context helps to decide whether an adaptation of the intervention conception to the target context is necessary. Thereby it should be noted why these criteria are important to consider for decision-making. It is also possible to identify what information about criteria is missing and whether it is important for the assessment. The aim is to differentiate and select influencing criteria for the further process. If the assessment of the intervention content, of the population and of the environment identifies criteria that make an intervention transfer impossible (e.g. high costs of the intervention), it becomes clear that the intervention is inappropriate *(criteria of the intervention, the population and the environment)*.	What are the characteristics of the population and the environment in the primary and target context? Which criteria are particularly relevant to consider?<br>Is there enough information about the primary and target context (population, environment) for a sound comparison of the criteria? If not, is missing information relevant for decision-making?<br>Are differences between primary and target context hindering transferability? Are there any criteria of the intervention content, of the population and of the environment which make a successful intervention transfer impossible? Why? Then another intervention may be more suitable.
If there are differences in relevant criteria of the population and the environment between primary and target context, it should be further analyzed whether and how the intervention could be successfully transferred.	Are there differences in outcome-relevant criteria of the population and the environment between primary and target context?<br>Which measures are possible to overcome barriers to transferability?

For example: it may be possible to overcome identified barriers with regard to the population and environment through suitable measures (e.g. providing resources).<br>Further, it should be considered how important intervention fidelity is, whether intervention core elements are known, and which adaptations are suitable for the target context (*criteria of the intervention content*). Thereby it is possible to determine outcome-relevant criteria of the population, the environment and the intervention, which should be empirically measured in the further process *(criteria of the intervention, the population and the environment)*.	How important is a "replication" of the primary intervention (intervention fidelity) with regard to the conception of the intervention?<br>Which adaptations of the intervention to the target context are needed with regard to the population characteristics and the environmental characteristics? Is it necessary to adapt the intervention to the population in the target context? Is it necessary to adapt the intervention to the environment in the target context?<br>Which outcome-relevant criteria of the population, the environment and the intervention should be measured empirically during the evaluation? How can they be operationalized?<br>Can moderating or mediating effects be expected?<br>What are the expected outcomes?
The comparison of relevant criteria between the primary and target context also serves to consider how comparable the outcomes of both contexts will be. Differences between the two contexts become transparent. The more different the two contexts are with regard to outcome-relevant criteria, the less comparable the outcomes will be. The decision-maker can decide whether the comparability of the outcomes in both contexts is relevant for the evaluation of success in the target context. The knowledge about important differences can be used to determine and describe the conditions under which the intervention should be successful in the target context *(criteria of the intervention, the population and the environment)*.	How important is the comparability of the outcomes in the primary and target context for the evaluation of intervention success (i.e. does the focus lie more on successful implementation of the intervention regardless of comparability of the outcomes between primary and target context or more on replication of the primary intervention with the aim of comparing the success of the primary and replicated intervention)?<br>Under what conditions should the intervention be effective in the target context?
The transfer criteria are intended to assist in the subsequent planning of the intervention transfer. They are related to the criteria of the population, environment and intervention. For example: the presence of an action plan in the intervention conception can include strategies that determine the transfer process. In general, the transfer process must be planned for the target context. It should be noted, however, which criteria of the transfer process tend to facilitate or hinder the comparability of outcomes between primary and target context.<br>For example: the evaluation design and the comparability of the outcome measures influence the comparability of the outcomes.	How can the intervention transfer be designed? Is there information about criteria of transfer from the primary context?<br>Which criteria are used and specified for the design of the transfer process for the target context?<br>Which criteria of the transfer process tend to facilitate or hinder the comparability of outcomes between primary and target context?<br>Which changes and adaptations are possible throughout the process?<br>Which criteria are useful for the documentation and/or evaluation of the process and outcome (quantitatively or qualitatively)?

However, comparing the outcomes of the primary and target context may not be the goal for determining transferability. Transferability of a health intervention can also be reached if success is achieved through systematic adaptations, e.g. of the intervention content, of environmental conditions (e.g. providing necessary resources) or other changes throughout the process. These changes should be documented for the evaluation. They can provide valuable insights into the mechanisms responsible for success *(criteria of the transfer)*.	

Overview of descriptive themes and criteria

Table S3 Overview of all descriptive themes, criteria and sub-criteria

**Higher-order theme:**<br>1. Criteria of the population in the primary and target context
**Descriptive theme:**<br>1.1 The population characteristics in the primary and target context in terms of…<br><br>**Criteria:**<br>*…the epidemiologic characteristics*<br>*…sociodemographic characteristics*<br>*…the cultural/social (including individual) characteristics*<br>*…cognitive characteristics*<br>*…socio-educational characteristics*
**Descriptive theme:**<br>1.2 The population's perceptions of health and health services in the primary and target context in terms of…<br><br>**Criteria:**<br>*…the health needs (regarding the health problem)*<br>*…the cooperation between providers and recipients*
**Descriptive theme:**<br>1.3 The population's attitude towards the intervention in the primary and target context in terms of…<br><br>**Criteria:**<br>*…the population demand for the intervention*<br>*…the acceptability of the intervention*<br>*…the motivation*

Higher-order theme:
2. Criteria of the intervention in the primary and target context

Descriptive theme:
2.1 Characteristics of the evidence base for comparison of primary and target context in terms of…

Criterion:
…utility/usefulness of primary evidence particularly with regard to the following
Sub-criteria:
- *level of transfer*
- *clearness and relevance of the research question/problem for decision-making*
- *detailed description and relevance of the population/sample for decision-making*
- *relevance of the outcome measurement for the target population and environment*
- *up-to-dateness of the intervention and relevance of the results for decision-making*
- *(anticipated) applicability of the intervention to the target population/groups and setting*
- *sufficient description of environmental conditions, processes, results and the intervention*
- *availability of documents and tools*

Criterion:
…quality of primary evidence particularly with regard to the following
Sub-criteria:
- *number of studies on the intervention and consistency of the results*
- *study design/study type and appropriateness for the research question*
- *appropriateness of sampling according to the study design*
- *ethical considerations*
- *appropriateness and rigor of measurement/data collection, assessed in accordance with the study design*
- *appropriateness and rigor of evaluation/data analysis, assessed in accordance with the study design*
- *bias and/or confounding under consideration of the study design*
- *appropriateness of interpretation of the results, e.g. of statistical tests/quantitative analyses, and presentation of the results*
- *generalizability/external validity*
- *level of evidence and/or grade of recommendation for adoption*

Descriptive theme:
2.2 Characteristics of the intervention content in the primary and target context in terms of

Criterion:
…the conception of the intervention in the primary and target context particularly with regard to the following

Sub-criteria:

- *the complexity/character of the intervention*
- *theoretical foundations or model and/or principles/methods and components*
- *the action plan for the transfer process*
- *tools and materials*
- *scale/reach and duration of the intervention*
- *costs of the intervention*

Criterion:
…the possibility of adaptations by keeping the primary intervention's fundamental nature and intervention fidelity particularly with regard to the following
Sub-criteria:

- *identification of transferable core elements/key functions*
- *identification of elements which are not transferable or need modification*
- *adaptation/modification of the specific form of the intervention*

Higher-order theme:
3. Criteria of the environment in the primary and target context

Descriptive theme:
3.1 Characteristics of policy and legislation in the primary and target context in terms of…

Criteria:
…national policy and political programs
…political climate and will
…local policy
…legislation relevant to transferability of the intervention

Descriptive theme:
3.2 Characteristics of coordination players in the primary and target context in terms of…

Criteria:
…types of partners, networks and their (formal or informal) involvement
…different personal and professional interests of stakeholders

**Descriptive theme:**<br>3.3 Characteristics of the health care system and service provision in the primary and target context in terms of…<br><br>**Criterion:**<br>*…the structure of the health care system and inherent services* particularly with regard to the following<br>**Sub-criteria:**<br>• *organization*<br>• *financing system*<br>• *alternative interventions available*<br><br>**Criterion:**<br>*…conditions of health service provision* particularly with regard to the following<br>**Sub-criteria:**<br>• *usual care conditions and treatment as usual*<br>• *professional expertise regarding the health problem and the new intervention*<br>• *financial resources and conditions of intervention funding*<br>• *resources for intervention delivery (availability and need)*<br>• *accessibility of the intervention*
**Descriptive theme:**<br>3.4 Characteristics of the local and organizational setting in the primary and target context in terms of…<br><br>**Criteria:**<br>*…physical and structural environmental conditions*<br>*…current existence of synergistic or antagonistic interventions*<br>*…the social/cultural local and/or organizational climate*<br>*…the general organizational structure and practice*<br>*…awareness of the intervention and readiness with regard to pre-existing and durable organizational (including political) will for intervention transfer*<br>*…decision-makers'/leaders' positive perception of the intervention and its importance/priority, their skills, status, and latitude for action*<br><br>**Criterion:**<br>*…support of decision-makers/leaders and (institutional and/or centralized) management* particularly with regard to the following

Sub-criteria:
- *adaptation of the intervention to the target group*
- *implementation of the intervention*
- *providing expertise, supervision, assistance and help*
- *sustaining professionals' motivation for involvement and action*

Criterion:
…providers' (professionals') perception and support of the intervention particularly with regard to the following
Sub-criteria:
- *need, utility, priority/importance and effectiveness*
- *acceptance/acceptability*
- *motivation and engagement*
- *financial, scientific and/or professional interest*

Higher-order theme:
4. Criteria of transfer from the primary to the target context

Descriptive theme:
4.1 Characteristics of communication in the target context in comparison to the primary context in terms of…

Criterion:
…overall communication by leaders for the coordination of an intervention particularly with regard to the following
Sub-criteria:
- *goals, a clear structure and expectations*
- *management of data flow*
- *(program) meetings*
- *providing results to stakeholders*

Criterion:
…quality of communication in multidisciplinary work and in teams particularly with regard to the following
Sub-criteria:
- *relation dynamics of stakeholders involved in the process*
- *defined and clear roles*
- *skills for working together*
- *information exchange*

**Descriptive theme:**<br>4.2 Characteristics of knowledge transfer in the target context in comparison to the primary context in terms of…<br><br>**Criterion:**<br>*…existence of a 'knowledge translation' process for the intervention* particularly with regard to the following<br>**Sub-criteria:**<br>• *support from (trained) specialists*<br>• *training of providers/ professionals*<br>• *knowledge for maintaining the (essential) core elements of the intervention (fidelity) while enabling adaptation to context (flexibility)*<br>• *links for knowledge exchange between researchers and stakeholders of the target context*
**Descriptive theme:**<br>4.3 Characteristics of adoption and implementation in the target context in comparison to the primary context in terms of…<br><br>**Criteria:**<br>*…strategies to reach, mobilize and engage the target population depending on characteristics of the recipients*<br>*…strategies to reach and involve different stakeholders from the beginning*<br>*…identification and addressing of implementation barriers and facilitators*<br>*…strategies of service delivery/intervention delivery*<br>*…successful pilot-testing of the intervention*<br>*…possibility of adaptations throughout the intervention's process, i.e. of the implementation process and/or intervention form by keeping essential (core) elements*
**Descriptive theme:**<br>4.4 Characteristics of the evaluation in the target context in comparison to the primary context in terms of…<br><br>**Criteria:**<br>*…evaluation/study design*<br>*…kind of assessment of processes and outcomes for measuring intervention success*<br>*…similarity of determination of effects of the primary and replicated intervention*<br><br>**Criterion:**<br>*…continuity and quality of evaluation throughout the transfer process* particularly with regard to the following<br>**Sub-criteria:**<br>• *kind and validity of information of the target context*<br>• *validity and reliability of measures*<br>• *continuity of monitoring and measuring success throughout the process*

**Descriptive theme:**<br>4.5 Characteristics of sustainability in the target context in comparison to the primary context in terms of…<br><br>**Criterion:**<br>*…sustainability* particularly with regard to the following<br>**Sub-criteria:**<br>• *intervention outcomes*<br>• *change of current practice/stability and sustainability of implementation*<br>• *key factors in intervention success*<br>• *stability of financing*
**Legend:** Descriptive themes and criteria underlie the higher-order themes population, intervention, environment and transfer, which are numbered from 1-4. The descriptive themes are numbered after each higher-order theme to facilitate the attribution to the higher-order theme. All criteria of transferability of health interventions relate to specific descriptive themes. Sub-criteria characterize a criterion in the form of specific aspects relevant to transferability. Criteria and sub-criteria are highlighted in italics.
