# Supplementary material for: Criteria for evaluating transferability of health interventions: a systematic review and thematic synthesis
Source: Implement Sci. 2018 Jun 26;13:88. doi: 10.1186/s13012-018-0751-8 (PMC6019740; doi:10.1186/s13012-018-0751-8)
Supplement: Supplementary file 4 — Description of the results of the descriptive themes. Descriptive text of the qualitative results of the thematic synthesis belonging to Table 2. (PDF 272 kb) [file 13012_2018_751_MOESM4_ESM.pdf]

Additional file 4: Description of the results of the descriptive themes

Descriptive themes

The descriptive themes categorize the criteria, facilitators and barriers of transferability of health interventions, which underlie the higher-order themes population, intervention, environment and transfer. They were constructed from the literature and each criterion was documented with the respective authors. Facilitators and barriers were added to enrich the understanding of the criteria. During the analysis of stage 1 and 2 it turned out that a sorting of criteria per transferability type was not relevant, as criteria were repeated by authors independent of the main work field. A generalization of the criteria was thus appropriate. In sum 14 descriptive themes, 44 criteria and 62 sub-criteria were constructed. Table 2 gives an overview of all criteria, Additional file 5 and 6 provide detailed tables (table S4 and S5) for the criteria with examples, facilitators and barriers.

1. Criteria of the population

Three themes for the population were constructed, which focus on the population's characteristics, their perceptions of health and health services and their attitudes towards the intervention. The term population here refers to a population, groups or persons who are to be addressed with the intervention. Thus, the population means the recipients of the intervention, but it can also include related persons who are involved in the intervention.

1.1 The population characteristics in the primary and target context

The descriptive theme population characteristics was supported by 26 articles (70%) and includes 5 criteria. The first criterion of the population characteristics addresses the *epidemiologic characteristics* of the population in the primary and target context regarding the health problem, which should be compared. It was supported by 18 articles (49%). Several potential factors were found in the literature, such as the health status/morbidity, baseline prevalence, incidence or risk, existing comorbidities, the medical history, complication rates and mortality [7-10, 17, 25, 39, 41, 46, 52, 53, 63, 67-72].

The second criterion, *sociodemographic characteristics*, was also supported by 18 articles (49%). Sociodemographic characteristics include, for example, sex, age, socioeconomic characteristics such as income, work and education, and sociodemographic diversity [7-10, 25, 37-39, 46, 52, 53, 55, 56, 67-71].

The *cultural/social, including individual characteristics,* build the third criterion of the population characteristics. It was supported by 15 articles (41%) and includes several

potential factors, namely people's history, migration, ethnicity and ethno-cultural diversity, religion, and people's worldview, values, lifestyle and configurations of identity [8-10, 25, 37-39, 41, 46, 52, 53, 55, 67, 69, 71].

The fourth criterion describes *cognitive characteristics* of the population and was supported by 5 articles (14%). Potential factors for transferability are cognition of the population depending on age, knowledge, language and educational achievement [8-10, 25, 52].

Socio-educational characteristics build the fifth criterion. It was supported by 7 articles (19%) and particularly refers to health education and literacy relevant for transferability, such as being informed, having awareness and understanding of the intervention and the conditions for access [8-10, 25, 37, 52, 55].

1.2 The population's perceptions of health and health services in the primary and target context

This descriptive theme regarding the population was supported by 13 articles (35%). It includes 2 criteria. The first criterion refers to the *health needs of the population* regarding the health problem and was supported by 9 articles (24%). It can also help to identify the need for the health intervention from the perspective of the population by potential factors such as risk perception, fatalism, help seeking, health care use, and response to treatment [7-9, 41, 46, 52, 53, 55, 67].

Eight articles (22%) supported the second criterion, *the cooperation between providers and recipients*, which includes the climate of trust and the involvement of recipients, for example being involved through patient-centeredness or collaboration as partners, such as receiving information and making decisions [9, 25, 37, 46, 47, 50, 52, 72]. One important facilitator for transferability, for example, may be to enable individual treatment decisions between provider and recipients when an intervention is transferred [25, 72]. According to Wegscheider [72], collective benefits can be assumed on the basis of studies of good quality when individual benefits are evident.

1.3 The population's attitude towards the intervention in the primary and target context

Thirteen articles (35%) supported the theme of the population's attitude towards the particular intervention of interest as an influence on transferability. The first criterion is the *population demand for the intervention*, that is, the extent to which the intervention is asked for or used by the population. This criterion was supported by 5 articles (14%) [9, 17, 37, 55, 69].

The second criterion, *the acceptability of the intervention*, was supported by 10 articles (27%). Potential factors are, for example, the social, cultural and ethical acceptability and the believe in the utility of the intervention [7-10, 25, 40, 47, 52, 55, 69].

The *motivation of the population* as the third criterion was also supported by 5 articles (14%) and includes potential factors such as willingness for participation, interests, responding to financial incentives, compliance and treatment continuity [8, 9, 17, 25, 46].

2. Criteria of the intervention

Two descriptive themes were constructed to categorize the criteria of the intervention, which address the evidence base of the intervention and the intervention content.

2.1 Characteristics of the evidence base for comparison of primary and target context

As described in the conceptual model, the characteristics of the primary context have a great influence on transferability of health interventions. The theme of the characteristics of the evidence base was supported by 29 articles (78%). Here, a particular focus is given to the utility and quality of the intervention's evidence as a prerequisite for transferability.

The first criterion is *utility/usefulness of the primary evidence*, which focuses on how useful the information from the primary context is for the target context. It contains 8 sub-criteria and was supported by 27 articles (73%):

The *level of transfer* has been already described in the model and means for example a transfer from experimental setting to real life setting or from a local to a national level [7, 8, 16, 17, 26, 38, 39, 41, 45, 46, 56, 64]. Further, the *clearness and relevance of the research problem for decision-making* [7, 40, 73], the *detail of description and relevance of the population for decision-making* [25, 37, 45, 48, 72, 73], and *the relevance of the outcome measurement for the target population and environment* are sub-criteria [7, 37, 49, 73]. The *up-to-dateness of the intervention and relevance of the results for decision-making* should also be considered (i.e. the relevance of the intervention to influence the problem in terms of the magnitude of effects, sustainability/long term effects and up-to-date-ness) [7, 8, 10, 17, 25, 37, 39, 40, 53, 64, 72, 73]. A further sub-criterion is the *(anticipated) applicability of the intervention to the target population/groups and setting* [7, 8, 10, 37, 39, 40, 74]. This criterion may be assessed with the help of criteria of the intervention content, the population and the environment to anticipate feasibility. To understand criteria and processes in the primary context, *a sufficient description* of the research is needed, that is, *the environmental conditions* (e.g. setting), *processes* (e.g. implementation), *results* (e.g. intended and unintended effects) [7, 9, 10, 17, 25, 37, 39, 40, 45, 47, 48, 55, 68, 73], *and the intervention for in depth understanding and application*

[17, 18, 26, 37-40, 45, 48, 55, 56, 68, 73]. Further, the *availability of documents and tools* is an influencing factor [8, 9, 17, 46, 47].

Several barriers and facilitators regarding the usefulness of primary evidence were identified. For example, a lot of evidence may be available, but a lack of time, resources or academic knowledge of decision-makers for searching and appraising evidence may lead to reduced assessment of potential studies for use in order to understand their transferability [7, 48]. On the other hand, a lack of evidence for addressing a health problem/an intervention may hinder or reduce transferability assessment [10, 72]. In general, conclusions from reviewing evidence for transferability may be limited due to poor description of the intervention [26], a lack of adequate process and contextual information [10, 37], limited data, and unknown suitability of an intervention for different settings or different populations, e.g. for disadvantaged groups [26, 37].

Various suggestions were made by authors to facilitate the usefulness of evidence, such as guidelines for transparent reporting of interventions [26], linked sources to process and contextual information in published papers, when information is too lengthy for publication [10], and reviews which include research-tested and practice-based studies with a range of study designs, assess internal as well as external validity and attempt to explain why interventions sometimes work and sometimes do not in different contexts in order to refine elements of intervention success or theory, and to take into account issues of relevance and transferability to improve recommendations for practice (e.g. by realist synthesis) [17, 37, 39, 64, 72]. Other facilitators are an increased reporting of external validity and generalizability in studies to improve the quality of the evidence base and the usefulness for decision-makers [7, 8, 46], and more research on different levels [26, 38, 45], depending on the character and objective of the intervention [8, 37], as well as more translational research comprising replication or dissemination research in new settings [16, 17, 37, 47], for example, large-scale, multisite high quality studies to determine if a program that works in one location or with one population works in other contexts before labelling it as "evidence-based" [16]. A further facilitator is the inclusion of qualitative approaches, which help to adapt the intervention to population needs, explain indicators, determinants of health and intervention outcomes (what and how it works), and build a basis for informing policy and practice in terms of transferability by exploring possible interactions among population, environment and intervention, which could be tested in further research [8, 26, 37, 39, 40, 46-50]. More facilitators as well as barriers referring to the evidence base can be found in Additional file 6 (table S5).

The second criterion *quality of primary evidence* was supported by 20 articles (54%). It is important to note that this criterion represents 10 overarching sub-criteria relevant to

transferability of health interventions independent of the study design and not an instrument for appraising evidence (see also Additional file 5, table S4):

The sub-criteria are *the number of studies on the intervention and consistency of the results* (e.g. effectiveness in different settings) [7, 16, 37, 39, 48, 64, 69, 73, 75], the *study design/study type and the appropriateness for the research question [7, 8, 37-39, 41, 48, 53, 69, 72, 73]*, *the appropriateness of sampling according to the study design* [7, 8, 16, 25, 37, 48, 63, 72, 73], *ethical considerations,* e.g. denying the intervention to a control group [8, 16, 48, 73], *appropriateness and rigor of measurement/data collection, assessed in accordance with the study design* [7, 16, 37, 48, 53, 73, 75], *appropriateness and rigor of evaluation/data analysis, assessed in accordance with the study design [8, 16, 25, 37-39, 48, 53, 73]*, *bias and/or confounding under consideration of the study design* [16, 37, 41, 69, 72, 73], *appropriateness of interpretation of the results* (e.g. strength, weakness and limitation of the study and appropriateness of the conclusion) [7, 8, 10, 16, 25, 37, 45, 72, 73, 75], *generalizability/external validity* (generalization to wider populations and settings) [7, 8, 10, 16, 17, 25, 37, 39, 45, 46, 48, 72-74] and the *level of evidence and/or grade of recommendation for adoption* [16, 37, 69, 72, 73]. For examples see Additional file 5 (table S4).

2.2 Characteristics of the intervention content in the primary and target context

This theme, which addresses the conception of the intervention and its potential for adaptation, was supported by 30 articles (81%).

The first criterion *conception of the intervention in the primary and target context* was supported by 24 articles (65%) and includes 6 sub-criteria: The *complexity and character of the intervention* has an influence on transferability, for example, the distinction between intervention elements that are highly context dependent (e.g. an education campaign for an immunization) or less dependent on context (e.g. efficacy of the vaccine among infants), and the extent to which change of current practice is needed [7-10, 16, 18, 26, 37-39, 45, 52, 53, 56, 60]. The *theoretical foundations or model [8, 37, 38, 40, 48, 53] and/or principles/methods and components* are a sub-criterion (e.g. focus and specification of the treatment such as changes to the environment, medication) [8, 9, 37-39, 47, 53], as well as *the action plan for the transfer process* (e.g. planning of implementation) [7-10, 37, 38, 47, 52], and *tools and materials* used [9, 40, 47]. Further sub-criteria are *the scale/reach and duration of the intervention* (e.g. dose and duration of the study intervention in comparison to usual care setting) [8, 9, 38, 45, 52, 67] and the *costs of the intervention* (e.g. expected coverage of the target population) [7, 8, 25, 38, 45, 52, 55, 67, 69, 73, 75].

Twenty articles (54%) supported the second criterion. It addresses *the possibility of adaptations [7-9, 26, 38, 40, 45-47, 52, 53, 55, 56, 64, 68, 70] by keeping the primary intervention's fundamental nature and intervention fidelity* [7-9, 16, 17, 25, 38, 40, 45-47, 53, 56, 68, 70]. Three sub-criteria were constructed:
One important sub-criterion for transferability is the *identification of transferable core elements/key functions*, which means essential processes to reach the objective, theory or elements in the intent and design of an intervention thought to be responsible for the effects [17, 46, 47, 52-56]. Some authors also mentioned the *identification of elements which are not transferable or need modification*, e.g. in considering a balance between the benefits of intervention fidelity and the need for adaptation [46, 56], as well as the *adaptation/modification of the specific form of the intervention* (e.g. potential, need for and form of adaptation in different contexts/settings) [7, 8, 38, 40, 46, 47, 53, 55, 56, 64, 68, 70].

Some authors also reported barriers and facilitators for the criteria of the intervention content (see Additional file 6, table S5). For example, reduced fidelity to the intervention and/or implementation [16, 17, 53] as well as a lack of guidance in customizing the intervention to the population may act as barriers to effectiveness of the intervention [53]. On the other hand, low intervention flexibility may lead to a professional view of the intervention being unacceptable or not suited to local needs and constraints [60], whereas decisions on forms or adaptations of the intervention by local actors may facilitate and positively influence interaction between the intervention and its context [56].

3. Criteria of the environment

Criteria of the environment are structured by 4 descriptive themes, which focus on policy and legislation, coordination players, characteristics of the health care system and services as well as on local and organizational settings.

3.1 Characteristics of policy and legislation in the primary and target context

This theme was supported by 14 articles (38%). It includes 4 criteria. *National policy and political programs* as the first criterion was supported by 7 articles (19%) with, for example, government's health policy and health initiatives as potential factors [7, 8, 18, 38, 50, 56, 71].

The second criterion, *political climate and will*, such as political priority and support of the intervention, was also supported by 7 articles (19%) [7, 10, 37, 38, 60, 64, 69].

Local policy, the third criterion, includes, for example, health programs, or transport policies and was supported by 6 articles (16%) [7, 8, 18, 37, 39, 56].
Two articles (5%) also mentioned the *legislation relevant to transferability of the intervention,* such as privacy law, laws on trade or professional practice [18, 75].

3.2. Characteristics of coordination players in the primary and target context

Seventeen articles (46%) supported this descriptive theme. The first of 2 criteria describes the *types of partners, networks and their (formal or informal) involvement*, which was supported by 13 articles (35%) and may include decision-makers, funding organizations, lobbyists, consumer associations, communities or professional networks relevant for the coordination of the intervention, for example, the existence of a steering committee or mechanisms for collaboration, governance, funding and information sharing [9, 16, 18, 37, 38, 40, 45-47, 49, 50, 52, 56].

The second criterion, supported by 8 articles (22%), *addresses different personal and professional interests of stakeholders,* which may influence transferability, for example, the degree of collaboration or competition or opposing interests and contested plans for action [8, 16, 18, 25, 37, 40, 56, 60]. One barrier may be, for example, protagonistic and antagonistic views in regarding the health issue as a social problem, which may lead to resistance by antagonists and reduce transferability [40].

3.3 Characteristics of the health care system and service provision in the primary and target context

This environmental theme was supported by 28 articles (76%) and contains two criteria. *The structure of the health care system and inherent services* is the first criterion, which was supported by 15 articles (41%) and includes 3 sub-criteria: *The organization of the health care system* has an influence on transferability of health interventions, such as the stability, institutions, sectors and interfaces, the structure of supply or quality mechanisms [17, 18, 25, 38, 50, 52, 68, 70, 71, 75]. Second, the *financing system* is an influencing sub-criterion, that is, the financial allocation and distribution of aspects of health care due to the understanding of justice in a society [8, 18, 26, 38, 41, 69, 71]. Regarding the transferability of an intervention under this aspect, the sub-criterion of *alternative interventions available* becomes relevant, e.g. under consideration of a need for intervention transfer [8, 17, 25, 69].

The second criterion, *conditions of health service provision,* was supported by 27 articles (73%). Five sub-criteria belong to this criterion:

The *usual care conditions and treatment as usual* mean general context-specific care conditions, effectiveness and appropriateness of usual treatment regarding the health problem under consideration of the conditions in target context compared to primary context (e.g. existing real-world conditions versus ideal study conditions) [7, 8, 16, 17, 25, 38, 41, 46, 47, 53, 55, 69]. Further, the *professional expertise regarding the health problem and the new intervention* are a sub-criterion [8, 9, 16, 25, 38, 39, 45, 47, 52, 68, 69], including training [45, 52, 68-70], skills and knowledge (e.g. work experience) [8-10, 26, 38, 68, 70]. *Financial resources and conditions of intervention funding* include, for example, conditions for reimbursement such as grant funding in studies and fee-for-service in care settings [7, 9, 18, 25, 38, 46, 52, 56, 64, 68, 70, 71, 75].

Further, *resources for intervention delivery (availability and need)* are a sub-criterion relevant for transferability [39, 41, 45], such as organization [10, 55, 68, 70], staff [7, 9, 47, 53], service infrastructure [8, 25, 37, 40, 47, 52, 53, 55, 75], space [47, 53], material and information [40, 47, 69] and time [16, 69]. Another condition of health service provision is the *accessibility of the intervention* [8-10, 25, 37, 41, 47, 52, 55], namely financial accessibility (i.e. costs of the intervention for the recipient) [9, 45, 52, 55, 69], sociocultural accessibility (e.g. service hours) [9, 38, 45, 52], and geographic accessibility (e.g. locations) [9, 10, 38, 45, 47, 55].

One barrier are differences in conditions of service provision in the primary context and target context, for example, differences in treatment as usual in a control group intervention in the primary context and usual treatment in community-based organizations in the target context [17, 41, 46, 53, 55, 75]. Such differences may lead to reduced comparability for the determination of effects for transferability [17, 41]. Treatment as usual should therefore be regarded as an own treatment, which should be defined and described in detail for the assessment of transferability [17]. Differences in service provision may also lead to a lack of resources for the intervention in the target context [46, 53] and may create substantial differences in the possibilities and costs per patient [75], or require specific local solutions [55] (see also Additional file 6, table S5).

3.4 Characteristics of the local and organizational setting in the primary and target context

This setting-specific theme for the assessment of transferability, which is characterized by 8 criteria, was supported by 23 articles (62%). It is closely connected to and complements the characteristics of the health care system and service provision.

The first criterion *physical and structural environmental conditions,* was supported by 6 articles (16%). A potential factor may be, for example urban/rural variation [18, 39, 47, 52, 64, 71].

The *current existence of synergistic or antagonistic interventions* as the second criterion was supported by 4 articles (11%) [7-9, 17]. For example, interventions or conditions pursuing the same objective may reduce desired effects of the new intervention and thus reduce transferability [17]. More barriers and facilitators regarding the local and organizational setting can be found in Additional file 6, table S5.

The third criterion represents *the social, cultural local and/or organizational climate*, and was supported by 10 articles (27%). Potential factors are, for example, the climate of social support or (prior) experiences of addressing the health problem [8-10, 18, 25, 38-40, 52, 64].

The *general organizational structure and practice*, the fourth criterion, was supported by 9 articles (24%) and addresses the organizational prerequisites in the setting, such as organizational competence, organizational financial and structural health, organizational hierarchy and capacity for change [9, 10, 16, 18, 25, 38, 39, 45, 52].

The fifth criterion is the *awareness of the intervention and readiness in terms of pre-existing and durable organizational (including political) will for transfer*, which was supported by 6 articles (16%) and includes potential factors such as organizational values and alignment of the intervention with an organization's mission and goals [9, 18, 40, 46, 52, 60].

The sixth criterion addresses *decision-makers'/leaders' positive perception of the intervention and its importance or priority [7, 9, 17, 18, 25, 37, 40, 52, 60, 69]*, as well as *their skills, status, and latitude for action*, for example, advocacy with necessary authority and being trusted and respected [9, 18, 38, 40, 60]. It was supported by 11 articles (30%).

The *support of decision-makers/leaders and (institutional and/or centralized) management* is the seventh criterion, which was supported by 7 articles (19%) and has 4 sub-criteria [9, 38, 40, 47, 52, 60, 69].
Support may be important in terms of *adaptation of the intervention to the target group* [9, 52, 60], *implementation of the intervention* [9, 40, 47, 52, 60], *providing expertise, supervision, assistance and help [38, 40, 60]* and *sustaining professionals' motivation for involvement and action* [40, 60].

The last criterion in this theme has 4 sub-criteria and was supported by 14 articles (38%). *It addresses providers' (professionals') perception and support of the intervention* in terms of *need, utility, priority/importance and effectiveness* [8, 9, 40, 46, 52, 69], *acceptance/acceptability* (e.g. in terms of values, safety, use of technology) [7, 9, 25, 38, 40, 52, 55, 60], *financial, scientific and/or professional interest* [8, 9, 60], and *motivation and engagement* (e.g. cooperation for intervention adherence and quality of care) [8, 9, 17, 18, 25, 40, 47, 52, 69].

4. Criteria of transfer

The criteria of transfer represent criteria for the realization of the transfer of health interventions, which have an influence on transferability. Five descriptive themes were constructed. They focus on characteristics of communication and knowledge transfer, on adoption and implementation, and on evaluation and sustainability.

4.1 Characteristics of communication in the target context in comparison to the primary context

The descriptive theme of communication was supported by 9 articles (24%) and includes 2 criteria. The first criterion has 4 sub-criteria and was supported by 7 articles (19%). It addresses *the overall communication by leaders for the coordination of an intervention* in terms of *goals, a clear structure and expectations* (e.g. of cost bearers) [40, 52, 55, 60], the *management of data flow* (e.g. for routinely, systematic collection of data) [40, 47, 49, 52], *(program) meetings* (e.g. to detect facilitators and barriers for transferability) [40, 47, 49, 60], and *providing results to stakeholders* [16, 40, 47, 49, 52, 60]. For example, the possibility for professionals to see positive results may promote their confidence and self-efficacy for implementation and sustainability [52] (see Additional file 6, table S5).

The *quality of communication in multidisciplinary work and in teams* is the second criterion, which was supported by 6 articles (16%). It addresses 4 sub-criteria. The *relation dynamics of stakeholders involved in the process* mean the degree of cooperation and interaction of stakeholders from different disciplines and in hierarchical structures [18, 40, 47, 52, 60]. The quality of communication further includes *defined and clear roles*, such as responsibilities and distribution of tasks [40, 47, 60, 75], as well as *skills for working together* [18, 40, 60], and *information exchange* during the process, for example, getting correct information [18, 40, 60, 75].

4.2 Characteristics of knowledge transfer in the target context in comparison to the primary context

Seventeen articles (76%) supported the theme of knowledge transfer, which exclusively addresses the criterion *existence of a knowledge transfer/knowledge translation process regarding the intervention* in terms of the sub-criteria *support from (trained) specialists* [9, 40, 46, 52, 53], *training of providers/professionals* [8-10, 38, 40, 46, 47, 52, 53, 68, 70], and imparting *knowledge for maintaining the (essential) core elements of the intervention (fidelity) while enabling adaptation to context (flexibility)* [17, 40, 46, 47, 53]. Further, *links for knowledge exchange between researchers and stakeholders of the target context* are a sub-criterion [9, 10, 26, 37, 38, 46, 50, 52, 56, 60].

Knowledge exchange between professionals and researchers/intervention experts [26, 38, 46, 50, 52, 53, 56, 60] may foster mutual learning and transferability [26, 40, 46, 50, 56, 60] by theorizing the intervention, describing key functions/core elements of the intervention, the implementation and the context [40, 46, 56]. It may also facilitate the feasibility of the intervention and implementation [40, 52, 53] by improving knowledge of implementation barriers and needs [46, 53] (see also Additional file 6, table S5).

4.3 Characteristics of adoption and implementation in the target context in comparison to the primary context

The descriptive theme of adoption and implementation was supported by 19 articles (51%) and includes 6 criteria. *Strategies to reach, mobilize and engage the target population depending on characteristics of the recipients* build the first criterion, which was supported by 9 articles (24%) [8-10, 38, 46, 47, 49, 52, 55].

The second criterion addresses *strategies to reach and involve different stakeholders from the beginning,* for example, professionals, decision-makers, policy-makers and community-members. It was supported by 11 articles (30%) [8-10, 18, 40, 46, 47, 50, 52, 55, 60].

The *identification and addressing of implementation barriers and facilitators,* supported by 8 articles (22%), is the third criterion [18, 39, 40, 45-47, 52, 53]. For example, a local needs assessment may facilitate the detection of non-transferable intervention elements and the need for adaptation of the intervention [46]. Addressing needs and barriers of professionals before implementing an intervention and adapting it to improve usability may enhance effectiveness of the intervention [52].

Strategies of service delivery, such as dealing with waiting time, build the fourth criterion, which was supported by 5 articles (14%) [37, 40, 47, 52, 55].

Successful pilot-testing of the intervention is the fifth criterion for transferability, which was also supported by 5 articles (14%) [37, 47, 52, 53, 55].

The sixth criterion (also supported by 14%) is the *possibility of adaptations throughout the intervention's process*, i.e. of the implementation process and/or the intervention form by keeping essential (core) elements [9, 39, 40, 52, 56].

4.4 Characteristics of the evaluation in the target context in comparison to the primary context

The theme characteristics of the evaluation was supported by 25 articles (68%). It addresses 4 criteria. The first criterion, supported by 7 articles (19%), is the choice of *the evaluation- or study design* in the target context compared to the primary context, which may have an influence on transferability, for example efficacy study with controlled conditions versus quasi-experimental or observational or case study [8, 16, 26, 37, 45, 47, 53].

The *kind of assessment of processes and outcomes for measuring intervention success,* supported by 21 articles (57%), is the second criterion [8, 16, 18, 37, 40, 47, 49, 53]. Examples include measuring population and patient/recipient-level outcomes and consideration of effect modification regarding health outcomes to detect (statistical) interactions between intervention and contextual factors [8, 16, 17, 25, 37, 38, 45, 47, 53, 63], measuring participation rate or reach [9, 26, 45, 47, 52, 63] or public health impact in terms of reach, outcomes/effectiveness, adoption, implementation and maintainance of the intervention (RE-AIM) [26, 45, 52, 63]. Further examples are interpretative/qualitative and/or quantitative evaluation of the intervention implementation process [8, 18, 26, 37, 39, 40, 47, 49, 52, 53] and evaluation of intervention fidelity, essential intervention (core) elements and/or theory [8, 16, 17, 39, 40, 45, 47, 52-54] and adaptation [26, 40, 47, 74].
The identification and scientific confirmation of key factors/core elements in successful intervention delivery are a facilitator and may enhance transferability of interventions, e.g. across communities [47, 54] (see Additional file 6, table S5).

Considering that, the *similarity of determination/measurement of effects of the primary and replicated intervention* is the third criterion, supported by 4 articles (11%) [8, 53, 68, 70].

The fourth criterion was supported by 11 articles (30%). It includes 3 sub-criteria and addresses the *continuity and quality of evaluation throughout the transfer process,* first in terms of *kind and validity of information of the target context,* for example, of baseline data by using the best available evidence for transferability assessment [10, 38, 39]. However, a barrier is that identifying all relevant factors for determining transferability may be impossible. Some factors will emerge over time and throughout the transfer process [38]. Further sub-criteria are *the validity and reliability of measures* during transfer [37, 40, 49] and the *continuity of monitoring and measuring success throughout the process* [9, 37, 38, 47, 49, 52, 53, 56].

4.5 Characteristics of sustainability in the target context in comparison to the primary context

The descriptive theme of sustainability of the intervention was supported by 10 articles (27%). As a single criterion with 4 sub-criteria it focuses on *sustainability of the intervention outcomes* [7, 16, 39, 53], as well as the *change of current practice/stability and sustainability of the implementation* [7, 37, 39, 52, 53]. Further, the *sustainability of key factors in intervention success* [17, 37, 47] is a sub-criterion. The last sub-criterion, which influences transferability of health interventions, is the *stability of financing* [46, 53, 56]. Unstable resources over time (e.g. funding, staff turnover) are a barrier which may lead to uncertainty around continuation of the intervention and may influence results and sustainability [46, 56].
