# Supplementary material for: Criteria for evaluating transferability of health interventions: a systematic review and thematic synthesis
Source: Implement Sci. 2018 Jun 26;13:88. doi: 10.1186/s13012-018-0751-8 (PMC6019740; doi:10.1186/s13012-018-0751-8)
Supplement: Supplementary file 5 — Detailed table of criteria with examples. Table S4. Comprehensive table with descriptive themes and criteria. (PDF 282 kb) [file 13012_2018_751_MOESM5_ESM.pdf]

Additional file 5: Detailed table of criteria with examples

Table S4 Comprehensive table with descriptive themes and criteria

**Higher-order theme:**<br>1. Criteria of the population in the primary and target context	
**Descriptive theme:**<br>1.1 The population characteristics in the primary and target context in terms of…	
**Criteria:**	**Authors:**
…the epidemiologic characteristics<br>e.g. health status/morbidity regarding the health problem, comorbidity, medical history, public health burden, baseline prevalence or incidence and the distribution across geographical areas and/or population groups/diverse societal groups, baseline risks (e.g. in terms of individual treatment the importance of the number needed to treat, assumed health benefit for patient and number needed to harm/side effects), risk status (also in control group of primary context), genetic/biological issues, complication rates, mortality.	Buffet et al., 2007; Burchett et al., 2011; Cambon et al., 2012; Cambon et al., 2013; Chase et al., 2009; Cuijpers et al., 2005; Feldstein & Glasgow, 2008; Glasgow et al., 1999; Guegan et al., 2011; Kelly et al., 2000; Kilbourne et al., 2007; Perleth, 2009; Schreyögg, 2004; Wang et al., 2005; Watts et al., 2011; Weinmann et al., 2012; Wegscheider, 2009; Whitley et al., 2011.
…sociodemographic characteristics<br>e.g. sex, age, socioeconomic characteristics such as income, work and education, sociodemographic diversity.	Buffet et al., 2007; Burchett et al., 2011; Cambon et al., 2012; Cambon et al., 2013; Chase et al., 2009; Cuijpers et al., 2005; Feldstein & Glasgow, 2008; Guegan et al., 2011; Kelly et al., 2000; Kilbourne et al., 2007; Perleth, 2009; Rychetnik et al., 2002; Saurman et al., 2014; Schoenwald & Hoagwood, 2001; Schreyögg, 2004; Villeval et al., 2016; Wang et al., 2005; Watts et al., 2011.
…the cultural/social (including individual) characteristics<br>e.g. people's history, ethnicity, migration, ethnocultural diversity, religion, lifestyle, configurations of identity, worldview and values.	Buffet et al., 2007; Cambon et al., 2012; Cambon et al., 2013; Cuijpers et al., 2005; Feldstein & Glasgow, 2008; Kelly et al., 2000; Kilbourne et al., 2007, Perleth, 2009; Rychetnik et al., 2002; Saurman et al., 2014; Schoenwald & Hoagwood, 2001; Schreyögg, 2004; Wang et al., 2005; Watts et al., 2011; Whitley et al., 2011.

…cognitive characteristics<br>e.g. cognition depending on age, knowledge, language, educational achievement.	Cambon et al., 2012; Cambon et al., 2013; Feldstein & Glasgow, 2008; Perleth, 2009; Wang et al., 2005.
…socio-educational characteristics<br>i.e. health education and literacy, being informed, having awareness and understanding of the intervention and conditions for access.	Cambon et al., 2012; Cambon et al., 2013; Feldstein & Glasgow, 2008; Perleth, 2009; Rychetnik et al., 2002; Saurman et al., 2014; Wang et al., 2005.
**Descriptive theme:**<br>1.2 The population's perceptions of health and health services in the primary and target context in terms of…	
**Criteria:**	**Authors:**
…the health needs (regarding the health problem)<br>e.g. risk perception, fatalism, help seeking, health care use, and response to treatment.	Buffet et al., 2007; Burchett et al., 2011; Cambon et al., 2012; Cambon et al., 2013; Feldstein & Glasgow, 2008; Kelly et al., 2000; Kilbourne et al., 2007; Saurman et al., 2014; Whitley et al., 2011.
…the cooperation between providers and recipients<br>i.e. climate of trust (prior knowledge of each other, past experiences, relationships) and involvement, e.g. through patient-centeredness for information giving and decision-making, as partners.	Cambon et al., 2013; Carter et al., 2009; Feldstein & Glasgow, 2008; Kelly et al., 2000; Perleth, 2009; Rychetnik et al., 2002; Van Royen et al., 2014; Wegscheider, 2009.
**Descriptive theme:**<br>1.3 The population's attitude towards the intervention in the primary and target context in terms of…	
**Criteria:**	**Authors:**
…the population demand for the intervention<br>i.e. extent to which the intervention is asked for or used by the population.	Cambon et al., 2013; Cuijpers et al., 2005; Rychetnik et al., 2002; Saurman et al., 2014; Weinmann et al., 2012.
…the acceptability of the intervention<br>e.g. social, cultural, ethical and philosophical acceptability or values and conviction, such as the believe in the utility of the intervention (e.g. positively: the intervention is a priority, recognized as effective and as having merit/benefit, referrals are accepted; or negatively: fear or bad expectations regarding the outcomes of a health condition or the diagnostic or treatment, a cognitive dissonance of the beneficiary in relation to the intervention).	Burchett et al., 2011; Cambon et al., 2012; Cambon et al., 2013; Carter et al., 2009; Cuijpers et al., 2005; Dixon-Woods et al., 2011; Perleth, 2009; Saurman et al., 2014; Wang et al., 2005 ; Feldstein & Glasgow, 2008.

…the motivation<br>e.g. interests, financial incentive, willingness for participation, compliance, treatment continuity.	Cambon et al., 2012; Cambon et al., 2013; Kelly et al., 2000; Perleth, 2009; Weinmann et al., 2012.
**Higher-order theme:**<br>2. Criteria of the intervention in the primary and target context	
**Descriptive theme:**<br>2.1 Characteristics of the evidence base for comparison of primary and target context in terms of…	
**Criterion:**	**Authors:**
***…utility/usefulness of primary evidence* with regard to the Sub-Criteria:**	
• *level of transfer*<br>e.g. transfer from efficacy studies/ experimental to real life; transfer from effectiveness studies which are closer to real life/ context to context (e.g. local to national, country to country); extend of control of context and standardization of the intervention; comparability/similarity of primary and target context e.g. in terms of a comparator/control group in RCTs in the primary setting, when treatment as usual in a control group is different from country to country (e.g. programs effective in a single location may not provide evidence in widespread dissemination).	Burchett et al., 2011; Cambon et al. 2012; Glasgow et al., 2003; Kelly et al., 2000; Muhlhausen, 2012; Rychetnick et al., 2012; Schoenwald & Hoagwood, 2001; Spencer et al., 2013; Villeval et al., 2016; Watts et al., 2011; Weinmann et al., 2012; Whitley et al., 2011.
• *clearness and relevance of the research question/problem for decision-making*<br>i.e. relevance for the target context.	Burchett et al., 2011; Dixon-Woods et al., 2011; Heller et al., 2008.
• *detailed description and relevance of the population/sample for decision-making*<br>e.g. size, inclusion/exclusion criteria, consideration of subgroups.	Glasgow et al., 2003; Heller et al., 2008; Pearson et al., 2011; Perleth, 2009; Rychetnik et al., 2002; Wegscheider, 2009.
• *relevance of the outcome measurement for the target population and environment*<br>e.g. indicators for the health problem of the target population, which can be reliably and validly operationalized for the target setting, (e.g. for stakeholders).	Burchett et al., 2011; Heller et al., 2008; Rychetnik et al., 2002; Tham et al., 2011.

• *up-to-dateness of the intervention and relevance of the results for decision-making*<br>i.e. relevance of the intervention to influence the problem in terms of magnitude of effects, sustainability/long term effects and up-to-dateness/date of research (e.g. in terms of the population and the life world of people and medical development).	Burchett et al., 2011; Cambon et al., 2012; Dixon-Woods et al., 2011; Heller et al., 2008; Kilbourne et al., 2007; Perleth, 2009; Rychetnik et al., 2002; Spencer et al., 2013; Wang et al., 2005; Watts et al., 2011; Wegscheider, 2009; Weinmann et al., 2012.
• *(anticipated) applicability of the intervention to the target population/groups and setting*<br>i.e. feasibility, e.g. absence of a necessary cofactor/essential element in the intervention's causal chain/for intervention delivery.	Burchett et al., 2011; Cambon et al. 2012; Dixon-Woods et al., 2011; Granstrom Ekeland & Grottland, 2015; Rychetnik et al., 2002; Wang et al., 2005; Watts et al., 2011.
• *sufficient description of environmental conditions*<br>e.g. political factors, resources, costs, structure, setting,<br>*processes*<br>e.g. objectives, the implementation and evaluation process and quality/monitoring,<br>*results*<br>e.g. anticipated/intended as well as unanticipated/ unintended effects and harms, stakeholders' responses, influences on sustainability and/or dissemination, efficiency/cost-effectiveness,<br>*and the intervention for in depth understanding and application*<br>e.g. components, adaptations, time, continuance, costs, details on how and why an intervention works effectively, adequate explanation of control group and control intervention for comparison, clarity of the intervention model.	Ashton et al., 2015; Burchett et al., 2011; Cambon et al., 2013; Carter et al., 2009; Chase et al., 2009; Dixon-Woods et al., 2011; Glasgow et al., 2003; Heller et al., 2008; Pearson et al., 2011; Perleth, 2009; Rychetnick et al., 2012; Rychetnik et al., 2002; Saurman et al., 2014; Schoenwald & Hoagwood, 2001; Villeval et al., 2016; Wang et al., 2005; Watts et al., 2011; Weinmann et al., 2012.
• *availability of documents and tools*<br>e.g. protocol or manual for assessment and intervention.	Cambon et al., 2012; Cambon et al., 2013; Carter et al., 2009; Kelly et al., 2000; Weinmann et al., 2012.

Criterion:	Authors:
***…quality of primary evidence* with regard to the Sub-Criteria:**	
• *number of studies on the intervention and consistency of the results* e.g. in systematic reviews and combining results in meta-analyses; congruence with other findings, with knowledge, with own experiences by readers or expert consensus; connection or congruence of findings with theory; effectiveness in different settings, variation of effects across population subgroups or by different implementation approaches.	Burchett et al., 2011; Cuijpers et al., 2005; Heller et al. 2008; Kidholm et al., 2012; Muhlhausen, 2012; Pearson et al., 2011; Rychetnik et al., 2002; Spencer et al., 2013; Watts et al., 2011.
• *study design/study type and appropriateness for the research question* e.g. randomized controlled trial, with strong internal validity and weak external validity; observational study, with strong external validity and weak internal validity; inclusion of qualitative approaches.	Burchett et al., 2011; Cambon et al. 2012; Cuijpers et al., 2005; Heller et al. 2008; Kilbourne et al., 2007; Pearson et al., 2011; Rychetnik et al., 2002; Schoenwald & Hoagwood, 2001; Watts et al., 2011; Wegscheider, 2009; Whitley et al., 2011.
• *appropriateness of sampling according to the study design* e.g. in terms of method, size, power, representativeness, diversity, inclusion and exclusion criteria, run-in-phase, randomization/non-randomization, response rate.	Burchett et al., 2011; Cambon et al., 2012; Glasgow et al., 1999; Heller et al. 2008; Muhlhausen, 2012; Pearson et al., 2011; Perleth, 2009; Rychetnik et al., 2002; Wegscheider, 2009.
• *ethical considerations* e.g. in terms of appropriateness of randomization and denying the intervention to a control group.	Cambon et al., 2012; Heller et al. 2008; Muhlhausen, 2012; Pearson et al., 2011.
• *appropriateness and rigor of measurement/data collection, assessed in accordance with the study design* e.g. of intervention features, of processes and specific outcomes (e.g. risk factors and exposures); methods and methodological quality (e.g. blinding in RCT, follow-up assessment), validity and reliability; adequacy of the outcome measures relative to program goals.	Burchett et al., 2011; Heller et al. 2008; Kidholm et al., 2012; Kilbourne et al., 2007; Muhlhausen, 2012; Pearson et al., 2011; Rychetnik et al., 2002.

• *appropriateness and rigor of evaluation/data analysis, assessed in accordance with the study design*<br>e.g. quantitative statistical tests/analyses and their precision; investigation of statistical interactions/effect modification or mediating influences on outcome; reporting of and dealing with dropouts and withdrawals; qualitative analysis of narratives and building of a general cross- case theory using sequences.	Cambon et al. 2012; Heller et al. 2008; Kilbourne et al., 2007; Muhlhausen, 2012; Pearson et al., 2011; Perleth, 2009; Rychetnik et al., 2002; Schoenwald & Hoagwood, 2001; Watts et al., 2011.
• *bias and/or confounding under consideration of the study design*<br>e.g. selection bias; performance bias; attrition bias; detection bias; addressing of as well as adequate dealing with confounders.	Cuijpers et al., 2005; Heller et al. 2008; Muhlhausen, 2012; Rychetnik et al., 2002; Wegscheider, 2009; Whitley et al., 2011.
• *appropriateness of interpretation of the results*<br>e.g. of statistical tests/quantitative analyses and presentation of the results (e.g. inclusion of absolute and relative risk, population impact, costs and cost-effectiveness, whether statistical interactions have been sought, understood and explained), of qualitative analyses and interpretations (e.g. through triangulation); credibility of findings and overall quality, strength, weakness and limitation of the study and appropriateness of the conclusion (e.g. conclusions on internal validity/causal relationship between the intervention and the outcome).	Burchett et al., 2011; Cambon et al. 2012; Glasgow et al., 2003; Heller et al. 2008; Kidholm et al., 2012; Muhlhausen, 2012; Perleth, 2009; Rychetnik et al., 2002; Wang et al., 2005; Wegscheider, 2009.
• *generalizability/external validity*<br>i.e. generalization to wider populations and settings, e.g. scope and boundaries for generalization, representativeness of population and setting, suggestions of settings for further research, possible threats to generalizability according to the study design (e.g. statistical calculations, limiting effects of sample selection and participation, the setting, history and constructs).	Burchett et al., 2011; Cambon et al., 2012; Glasgow et al., 2003; Granstrom Ekeland & Grottland, 2015; Heller et al. 2008; Kelly et al., 2000; Muhlhausen, 2012; Pearson et al., 2011; Perleth, 2009; Rychetnik et al., 2002; Wang et al., 2005; Watts et al., 2011; Wegscheider, 2009; Weinmann et al., 2012.
• *level of evidence and/or grade of recommendation for adoption*<br>e.g. in a guideline.	Cuijpers et al., 2005; Heller et al. 2008; Muhlhausen, 2012; Rychetnik et al., 2002; Wegscheider, 2009.

**Descriptive theme:**<br>2.2 Characteristics of the intervention content in the primary and target context in terms of…	
**Criterion:**	**Authors:**
…the conception of the intervention in the primary and target context **with regard to the**<br>**Sub-Criteria:**	
• *the complexity/character of the intervention*<br>e.g. structural, social, political character and context-dependence, such as distinction between components of interventions that are highly context dependent (e.g. a public education campaign for immunization) and those that may be less so (e.g. efficacy of the vaccine itself among healthy infants); the extent to which change of current practice is needed (e.g. less complex interventions may need less training, support and monitoring for intervention fidelity).	Ashton et al., 2015; Burchett et al., 2011; Cambon et al. 2012; Cambon et al., 2013; Glasgow et al., 2003; Feldstein & Glasgow, 2008; Kilbourne et al., 2007; Muhlhausen, 2012; Rychetnik et al., 2012; Rychetnik et al., 2002; Schoenwald & Hoagwood, 2001; Trompette et al., 2014; Villeval et al., 2016; Wang et al., 2005; Watts et al., 2011.
• *theoretical foundations or model and/or principles/methods and components*<br>e.g. focus and specification of the treatment such as educational activities, communication, specific training, changes to the environment, medication.	Cambon et al., 2012; Cambon et al., 2013; Carter et al., 2009; Dixon-Woods et al., 2011; Kilbourne et al., 2007; Pearson et al., 2011; Rychetnik et al., 2002; Schoenwald & Hoagwood, 2001; Watts et al., 2011.
• *the action plan for the transfer process*<br>e.g. planning of implementation training, support and sustainability from the start, recipients' involvement in intervention planning and participation, communication mechanisms, development/design of intervention strategies, who delivers and how, group size, language used according to population needs, whether manuals are used and how comprehensive and prescriptive they are.	Burchett et al., 2011; Cambon et al., 2012; Cambon et al., 2013; Carter et al., 2009; Feldstein & Glasgow, 2008; Rychetnik et al., 2002; Schoenwald & Hoagwood, 2001; Wang et al., 2005.
• *tools and materials*<br>for the application of the intervention.	Cambon et al., 2013; Carter et al., 2009; Dixon-Woods et al., 2011.

• *scale/reach and duration of the intervention*<br>e.g. reach to population subgroups, dose or duration of the intervention/study in comparison to usual care setting, length of sessions and sequence of sessions (e.g. in dependence on financing of the intervention).	Buffet et al., 2007; Cambon et al., 2012; Cambon et al., 2013; Feldstein & Glasgow, 2008; Glasgow et al., 2003; Schoenwald & Hoagwood, 2001.
• *costs of the intervention*<br>e.g. possibilities and costs per patient/recipient, expected coverage of the target population, affordability in terms of financial and incidental costs, methods of calculating costs of treatment or the intervention (e.g. including training for professionals, cost-effectiveness).	Buffet et al., 2007; Burchett et al., 2011; Cambon et al., 2012; Cuijpers et al., 2005; Feldstein & Glasgow, 2008; Glasgow et al., 2003; Heller et al., 2008; Kidholm et al., 2012; Perleth, 2009; Saurman et al., 2014; Schoenwald & Hoagwood, 2001.
**Criterion:**	**Authors:**
***…the possibility of adaptations by keeping the primary intervention's fundamental nature and intervention fidelity*, further specified by the following**<br>**Sub-Criteria:**	Burchett et al., 2011; Cambon et al., 2012; Cambon et al. 2013; Carter et al., 2009; Chase et al., 2009; Dixon-Woods et al., 2011; Feldstein & Glasgow, 2008; Glasgow et al., 2003; Guegan et al., 2011; Kelly et al., 2000; Kilbourne et al., 2007; Muhlhausen, 2012; Perleth, 2009; Rychetnick et al., 2012; Saurman et al., 2014; Schoenwald & Hoagwood, 2001; Spencer et al., 2013; Villeval et al., 2016; Weinmann et al., 2012.
• *identification of transferable core elements/key functions*<br>i.e. essential processes to reach the objective; theory or elements/features in the intent and design of an intervention thought to be responsible for effects (e.g. through core elements defined by theory, experience in implementing the intervention, or a formal component analysis of its procedures).	Carter et al, 2009; Feldstein & Glasgow, 2008; Kelly et al., 2000; Pawson, 2003; Saurman et al., 2014; Kilbourne et al., 2007; Villeval et al., 2016; Weinmann et al., 2012.
• *identification of elements which are not transferable or need modification*<br>e.g. in considering a complex balance between the benefits of encouraging intervention fidelity and the need for adaptation.	Kelly et al., 2000; Villeval et al., 2016.

• *adaptation/modification of the specific form of the intervention* e.g. potential of the intervention for adaptation to the population or setting; need for and form of adaptation in different contexts/settings, such as flexibility of specific activities, own modification of treatment protocols or tools and management by retaining main principles, adaptation to culture, language, needs, preferences or risk issues of the population, adaptation in terms of intervention delivery and content (e.g. using the intervention for specific groups, using examples, situations, and exercises to meet population or local needs, adaptation of length of sessions); extend of adaptation/modification over time or after evaluation).	Burchett et al., 2011; Cambon et al. 2012; Carter et al., 2009; Chase et al., 2009; Dixon-Woods et al., 2011; Guegan et al., 2011; Kelly et al., 2000; Kilbourne et al., 2007; Saurman et al., 2014; Schoenwald & Hoagwood, 2001; Spencer et al., 2013; Villeval et al., 2016.
**Higher-order theme:**<br>3. Criteria of the environment in the primary and target context	
**Descriptive theme:**<br>3.1 Characteristics of policy and legislation in the primary and target context in terms of…	
**Criteria:**	**Authors:**
…national policy and political programs<br>e.g. government's health policy and health initiatives (e.g. clearly articulated strategy for improving service integration); availability and coordination of social welfare benefits.	Ashton, 2015; Burchett et al., 2011; Cambon et al., 2012; Schoenwald & Hoagwood, 2001; Schreyögg, 2004; Van Royen et al., 2014; Villeval et al., 2016.
…political climate and will<br>e.g. political priority, acceptance of the intervention, support of the intervention, mandates.	Burchett et al., 2011; Cuijpers et al., 2005; Rychetnik et al., 2002; Schoenwald & Hoagwood, 2001; Spencer et al., 2013; Trompette et al., 2014; Wang et al., 2005.
…local policy<br>e.g. health programs, housing, pensions and transport policies.	Ashton, 2015; Burchett et al., 2011; Cambon et al., 2012; Rychetnik et al., 2002; Villeval et al., 2016; Watts et al., 2011.
…legislation relevant to transferability of the intervention<br>e.g. general legislation or regulations such as privacy law, laws on trade practices or human rights legislation, laws and regulations for professional competencies and scopes of practice or standards of service and safety.	Ashton, 2015; Kidholm et al., 2012.

**Descriptive theme:**<br>3.2 Characteristics of coordination players in the primary and target context in terms of…	
**Criteria:**	**Authors:**
…types of partners, networks and their (formal or informal) involvement<br>e.g. decision-makers, funding organizations, lobbyists, consumer associations, communities, and professional networks relevant for the coordination of the intervention (e.g. networks of professionals and of health centers; information exchange networks to learn about effective interventions);<br>e.g. evaluability assessment - a priori agreement about the outcomes from important stakeholders' perspectives (e.g. in terms of types of evidence for decision-making and the evaluation questions, including representatives from all involved departments and staff roles as advisors in the form of a decision-making committee);<br>e.g. district-wide cooperation for the development of appropriate mechanisms for governance, funding and information sharing;<br>e.g. a reference group or coordinating group with representatives from the local community, regional health authorities, and general practice support agencies for creating an infrastructure, conduction of research and dissemination of findings (for encouraging spread, sharing best practice, observing results, adjusting processes and protocols and facilitating service);<br>e.g. collaboration between service providers, researchers and funding agencies for intervention fidelity and adaptation.	Ashton, 2015; Cambon et al., 2013; Carter et al., 2009; Dixon-Woods et al., 2011; Feldstein & Glasgow, 2008; Glasgow et al., 2003; Kelly et al., 2000; Muhlhausen, 2012; Rychetnik et al., 2002; Schoenwald & Hoagwood, 2001; Tham et al., 2011; Van Royen et al., 2014; Villeval et al., 2016.
…different personal and professional interests of stakeholders<br>e.g. protagonists and antagonists regarding the health problem and the need for the intervention, differences in identities, roles, and interests, differences in knowledge and expertise, competing recommendations of different groups for decisions, degree of collaboration or competition amongst funders and/or providers, conflicting or opposing interests and contested plans for action.	Ashton, 2015; Cambon et al., 2012; Dixon-Woods et al., 2011; Muhlhausen, 2012; Perleth, 2009; Rychetnik et al., 2002; Trompette et al., 2014; Villeval et al., 2016.

**Descriptive theme:**<br>3.3 Characteristics of the health care system and service provision in the primary and target context in terms of…	
**Criterion:**	**Authors:**
***…structure of the health care system and inherent services* with regard to the**<br>**Sub-criteria:**	
• *organization*<br>e.g. relative stability of the wider health system; broad institutional arrangements that give shape to a health system, such as different health care/treatment sectors and interfaces (e.g. hospital treatment, number of hospital beds and community-based treatment approaches), the existence of organized general practice, roles and responsibilities of key agencies and/or payers, competition, structure of supply, incentives, referral systems, monitoring and accountability arrangements, efficiency and quality control or quality improvement mechanisms and surveillance.	Ashton, 2015; Chase et al., 2009; Feldstein & Glasgow, 2008; Guegan et al., 2011, Kidholm et al., 2012; Perleth, 2009; Schoenwald & Hoagwood, 2001; Schreyögg, 2004; Van Royen et al., 2014; Weinmann et al., 2012.
• *financing system*<br>i.e. allocation and distribution of aspects of health care due to the understanding of justice, ethical and moral norms in a society, such as the scope of the demand principle for financial protection of disease risks or granting of a minimum access to health care, funding procedures and forms of redistribution (such as in a welfare state), and form and scope of the financing of services (e.g. by funding agencies, single funding stream, pooling of budgets, methods of provider remuneration/salary).	Ashton, 2015; Cambon et al., 2012; Cuijpers et al., 2005; Rychetnik et al., 2012; Schoenwald & Hoagwood, 2001; Schreyögg, 2004; Whitley et al., 2011.
• *alternative interventions available*<br>e.g. under consideration of the need for intervention transfer.	Cambon et al., 2012; Cuijpers et al., 2005; Perleth, 2009; Weinmann et al., 2012.

Criterion:	Authors:
**…*conditions of health service provision* with regard to the Sub-criteria:**	
• *usual care conditions and treatment as usual* i.e. general context-specific care conditions regarding the health problem, effectiveness and appropriateness of usual treatment regarding the health problem under consideration of the conditions in target context compared to primary context (e.g. ideal study conditions versus usual care conditions/real-world conditions such as usual frequency of treatment of the problem; e.g. variety of therapies and methods available and needed according to different expertise of providers regarding the health problem in the target context).	Burchett et al., 2011; Cambon et al., 2012; Carter et al., 2009; Cuijpers et al., 2005; Kelly et al., 2000; Kilbourne et al., 2007; Muhlhausen, 2012; Perleth, 2009; Saurman et al., 2014; Schoenwald & Hoagwood, 2001; Weinmann et al., 2012; Whitley et al., 2011.
• *professional expertise regarding the health problem and the new intervention* e.g. type of practitioner such as physician, psychologist, social worker or other professional training skills and knowledge (including work experience).	Cambon et al., 2012; Cambon et al., 2013; Carter et al., 2009; Chase et al., 2009; Cuijpers et al., 2005; Feldstein & Glasgow, 2008; Glasgow et al., 2003; Guegan et al., 2011; Muhlhausen, 2012; Perleth, 2009; Rychetnik et al., 2012; Schoenwald & Hoagwood, 2001; Wang et al., 2005; Watts et al., 2011.
• *financial resources and conditions of intervention funding* e.g. in terms of economic climate, such as increasing expenditure on health and social care or period of fiscal constraint, specific distribution of resources and financial conditions for reimbursement of the intervention, for training, for evaluation, methods of payment for treatment (e.g. grant funding in studies and fee-for-service in care settings).	Ashton, 2015; Burchett et al., 2011; Cambon et al., 2013; Chase et al., 2009; Feldstein & Glasgow, 2008; Guegan et al., 2011, Kelly et al., 2000; Kidholm et al., 2012; Perleth, 2009; Schoenwald & Hoagwood, 2001; Schreyögg, 2004; Spencer et al., 2013; Villeval et al., 2016.
• *resources for intervention delivery (availability and need)* such as organization (e.g. for training, for meeting volume and need, consideration of conditions for availability and efficiency), staff, service infrastructure (e.g. equipment, media, technology, existence and interoperability of clinical or administrative systems such as electronic medical records or other systems for data gathering and decision support), space, material and information available, time (e.g. also for evaluation).	Burchett et al., 2011; Cambon et al., 2012; Cambon et al., 2013; Carter et al., 2009; Chase et al., 2009; Cuijpers et al., 2005; Dixon-Woods et al., 2011; Feldstein & Glasgow, 2008; Glasgow et al., 2003; Guegan et al., 2011; Kidholm et al., 2012; Kilbourne et al., 2007; Muhlhausen, 2012; Perleth, 2009; Rychetnik et al., 2002; Saurman et al., 2014; Wang et al., 2005; Watts et al., 2011; Whitley et al., 2011.

• *accessibility of the intervention*<br>Financial accessibility (i.e. costs of the intervention for the patient/ receiver), sociocultural accessibility (e.g. complexity of procedure for receiver, service hours), geographic accessibility (e.g. locations, proximity to the consumer in time and distance).	Cambon et al., 2012; Cambon et al., 2013; Carter et al., 2009; Cuijpers et al., 2005; Feldstein & Glasgow, 2008; Glasgow et al., 2003; Perleth, 2009; Rychetnik et al., 2002; Saurman et al., 2014; Schoenwald & Hoagwood, 2001; Wang et al., 2005; Whitley et al., 2011.
**Descriptive theme:**<br>3.4 Characteristics of the local and organizational setting in the primary and target context in terms of…	
**Criteria:**	**Authors:**
…physical and structural environmental conditions<br>e.g. geographic climate and factors such as size of setting, urban/rural variation, community resources to assist health service conditions (such as available infrastructure from other sources than the targeted health service).	Ashton et al., 2015; Carter et al., 2009; Feldstein & Glasgow, 2008; Schreyögg, 2004; Spencer et al., 2013, Watts et al., 2011.
…current existence of synergistic or antagonistic interventions<br>e.g. opposing objectives, messages, interventions or conditions pursuing the same objective.	Burchett et al., 2011; Cambon et al., 2012; Cambon et al., 2013; Weinmann et al., 2012.
…social/cultural local and/or organizational climate<br>e.g. in terms of social norms and morale, climate of risk taking, climate of social support (e.g. through families, friends, coworkers or other recipients with similar conditions), demands (e.g. family or work demands, competing demands), or in terms of (prior) synergistic experiences or antagonistic experiences such as passive event that generated mistrust, passive but potentializing event or protagonistic or antagonistic experiences of addressing the health problem.	Ashton, 2015; Cambon et al., 2012; Cambon et al., 2013; Dixon-Woods et al., 2011; Feldstein & Glasgow, 2008; Perleth, 2009; Schoenwald & Hoagwood, 2001;<br>Spencer et al., 2013; Wang et al., 2005; Watts et al., 2011.

…general organizational structure and practice<br>e.g. in terms of organizational competence (e.g. of a study center), size, organizational financial and structural health, organizational hierarchy, capacity for change such as existing infrastructure/support structure to take on implementation and sustainability (e.g. coordination across departments for seamless transition between service elements), workforce and workflows, anticipated job longevity or turnover (e.g. time-limited position in a study versus position in a practice setting), staff history, salary structure, time frames, a dedicated team for implementation, multidisciplinary teamwork and information sharing, possibility to try the intervention (trialability), to evaluate the intervention/see results (observability) and to terminate it in case of ineffectiveness (reversibility).	Ashton, 2015; Cambon et al., 2013; Feldstein & Glasgow, 2008; Glasgow et al., 2003; Muhlhausen, 2012; Perleth, 2009; Schoenwald & Hoagwood, 2001; Wang et al., 2005; Watts et al., 2011.
…awareness of the intervention and readiness in terms of pre-existing and durable organizational (including political) will for transfer<br>e.g. in terms of organizational values, alignment of the intervention with an organization's mission and goals (e.g. implementation becomes a goal), perceived advantage of adoption, timing of introduction of the intervention, readiness of senior leaders and mid-level-managers and frontline staff.	Ashton, 2015; Cambon et al., 2013; Dixon-Woods et al., 2011; Feldstein & Glasgow, 2008; Kelly et al., 2000; Trompette et al., 2014.
…decision-makers'/leaders' positive perception of the intervention and its importance/priority (e.g. need), their skills, status, and latitude for action<br>e.g. advocacy with necessary authority, being trusted and respected.	Ashton et al., 2015; Burchett et al., 2011; Cambon et al., 2013; Cuijpers et al., 2005; Dixon-Woods et al., 2011; Feldstein & Glasgow, 2008; Perleth, 2009; Rychetnik et al., 2002; Schoenwald & Hoagwood, 2001; Trompette et al., 2014; Weinmann et al., 2012.
**Criterion:**	**Authors:**
**…*support of decision-makers/leaders and (institutional and/or centralized) management* particularly in terms of the Sub-criteria:**	Cambon et al., 2013; Carter et al., 2009; Cuijpers et al., 2005; Dixon-Woods et al., 2011; Feldstein & Glasgow, 2008; Schoenwald & Hoagwood, 2001; Trompette et al., 2014.

• *adaptation of the intervention to the target group*	Cambon et al., 2013; Feldstein & Glasgow, 2008; Trom-pette et al., 2014.
• *implementation of the intervention*	Cambon et al., 2013; Carter et al., 2009; Dixon-Woods et al., 2011; Feldstein & Glasgow, 2008; Trompette et al., 2014.
• *providing expertise, supervision, assistance and help* e.g. through mentorship, for validity of measurement.	Dixon-Woods et al., 2011; Trompette et al., 2014; Schoen-wald & Hoagwood, 2001.
• *sustaining professionals' motivation for involvement and action*	Dixon-Woods et al., 2011; Trompette et al., 2014.
Criterion:	**Authors:**
**…*providers' (professionals') perception and support of the intervention* with regard to the Sub-criteria:**	
• *need utility, priority/importance and effectiveness* e.g. expectation of sustainability.	Cambon et al., 2012; Cambon et al., 2013; Cuijpers et al., 2005; Dixon-Woods et al., 2011; Feldstein & Glasgow, 2008; Kelly et al., 2000.
• *acceptance/acceptability* e.g. in terms of social, cultural, ethical, philosophical values, norms or conviction, safety, use of technology.	Burchett et al., 2011; Cambon et al., 2013, Dixon-Woods et al., 2011; Feldstein & Glasgow, 2008; Perleth, 2009; Saurman et al., 2014; Schoenwald & Hoagwood, 2001; Trompette et al., 2014.
• *financial, scientific and/or professional interest*	Cambon et al., 2012; Cambon et al., 2013; Trompette et al., 2014.
• *motivation and engagement* e.g. cooperation for intervention adherence and quality of care.	Ashton, 2015; Cambon et al., 2012; Cambon et al., 2013; Carter et al., 2009; Cuijpers et al., 2005; Dixon-Woods et al., 2011; Feldstein & Glasgow, 2008; Perleth, 2009; Wein-mann et al., 2012.

**Higher-order theme:**<br>4. Criteria of transfer from the primary to the target context	
**Descriptive theme:**<br>4.1 Characteristics of communication in the target context in comparison to the primary context in terms of…	
**Criterion:**	**Authors:**
***…overall communication by leaders for the coordination of an intervention* with regard to the**<br>**Sub-Criteria:**	
• *goals, a clear structure and expectations*<br>e.g. of cost bearors; e.g. for participation of organizations.	Dixon-Woods et al., 2011; Feldstein & Glasgow, 2008; Saurman et al., 2014; Trompette et al., 2014.
• *management of data flow*<br>e.g. for routinely, systematic collection of data and data sharing.	Carter et al., 2009; Dixon-Woods et al., 2011; Feldstein & Glasgow, 2008; Tham et al., 2011.
• *(program) meetings*<br>e.g. implementation meeting, interim meetings, post-program meeting for outcome presentation, to detect facilitators and barriers or openly discuss any problems and ways to overcome them.	Carter et al., 2009; Dixon-Woods et al., 2011; Tham et al., 2011; Trompette et al., 2014.
• *providing results to stakeholders*<br>i.e. feedback to all relevant stakeholders, including professionals and recipients (e.g. reporting results in a timely manner with plain language summaries, oral presentations or key community events; e.g. to readjust and strengthen professionals' motivation to participate, to motivate sustained efforts and provide a sense of progress).	Carter et al., 2009; Dixon-Woods et al., 2011; Feldstein & Glasgow, 2008; Muhlhausen, 2012; Tham et al., 2011; Trompette et al., 2014.
**Criterion:**	**Authors:**
***…quality of communication in multidisciplinary work and in teams* with regard to the**<br>**Sub-criteria:**	

• *relation dynamics of stakeholders involved*<br>e.g. the degree of cooperation and interaction of stakeholders from different disciplines and in hierarchical structures, such as managers and field participants, local coordinators, physicians and volunteers in the community or doctors and nurses (e.g. making experience of running the program together, sense of community).	Ashton, 2015; Carter et al., 2009; Dixon-Woods et al., 2011; Feldstein & Glasgow, 2008; Trompette et al., 2014.
• *defined and clear roles*<br>e.g. responsibilities for coordination, financial management and communication, distribution of tasks for intervention delivery and control of intervention fidelity by health professionals.	Carter et al., 2009; Dixon-Woods et al., 2011; Kidholm et al., 2012; Trompette et al., 2014.
• *skills for working together*<br>e.g. for collaboration, commitment, negotiating tension and conflict.	Ashton, 2015; Dixon-Woods et al., 2011; Trompette et al., 2014.
• *information exchange*<br>e.g. for building rapport or consensus, getting correct information, getting to know what everyone does (e.g. for improvement of the intervention in regular meetings).	Ashton, 2015; Dixon-Woods et al., 2011; Kidholm et al., 2012; Trompette et al., 2014.
**Descriptive theme:**<br>4.2 Characteristics of knowledge transfer in the target context in comparison to the primary context in terms of…	
**Criteria:**	**Authors:**
***…the existence of a knowledge transfer/ 'knowledge translation' process regarding the intervention* particularly with regard to the following**<br>**Sub-criteria:**	
• *support from (trained) specialists*<br>e.g. scientific and methodological support for intervention transfer.	Cambon et al., 2013; Dixon-Woods et al., 2011; Feldstein & Glasgow, 2008; Kelly et al., 2000; Kilbourne et al., 2007.

• *training of providers/ professionals*<br>e.g. in terms of implementation, scientific, practical-experiential know-how, education and coaching on the intervention, knowledge on safety issues, maintaining competencies (e.g. under consideration of their views, experiences, targets, attitudes, skills and self-efficacy), implementation of a routine or ritual with no required mobilization.	Cambon et al., 2012; Cambon et al., 2013; Carter et al., 2009; Chase et al., 2009; Dixon-Woods et al., 2011; Feldstein & Glasgow, 2008; Guegan et al., 2011; Kelly et al., 2000; Kilbourne et al., 2007; Schoenwald & Hoagwood, 2001; Wang et al., 2005.
• *knowledge for maintaining the (essential) core elements of the intervention (fidelity) while enabling adaptation to context (flexibility)*<br>e.g. knowledge and compliance of all stakeholders for institutionalizing the good practice in organizations under consideration of effectiveness (e.g. by a manual for intervention fidelity).	Carter et al., 2009; Dixon-Woods et al., 2011; Kelly et al., 2000; Kilbourne et al., 2007; Weinmann et al., 2012.
• *links for knowledge exchange between researchers and stakeholders of the target context*<br>e.g. established links with the promotor, providers and/or researchers of the primary intervention and with evaluators, practitioners, recipients and/or policy-makers in the target context, (e.g. to take lessons from the primary intervention into account, for mutual learning).	Cambon et al., 2013; Feldstein & Glasgow, 2008; Kelly et al., 2000; Rychetnick et al., 2012; Rychetnik et al., 2002; Schoenwald & Hoagwood, 2001; Trompette et al., 2014; Van Royen et al., 2014; Villeval et al., 2016; Wang et al., 2005.
**Descriptive theme:**<br>4.3 Characteristics of adoption and implementation in the target context in comparison to the primary context in terms of…	
**Criteria:**	**Authors:**
…strategies to reach, mobilize and engage the target population depending on characteristics of the recipients<br>such as raising awareness, (e.g. with community forums and articles in the local newspaper), recruitment methods, incentives for participation (e.g. compensation), referrals, atmosphere, providing confidentiality, volunteerism, autonomy of participants, addressing of health literacy, input, patient-centered strategies such as collaboration in setting goals and action plans, involvement and engagement of the community population, community education or training, site visits.	Cambon et al., 2012; Cambon et al., 2013; Carter et al., 2009; Feldstein & Glasgow, 2008; Kelly et al., 2000; Saurman et al., 2014; Schoenwald & Hoagwood, 2001; Tham et al., 2011; Wang et al., 2005.

…strategies to reach and involve different stakeholders from the beginning<br>e.g. professionals, decision-makers, policy-makers, community-members, e.g. through an invitation, raising awareness, advocating for a supportive political and social environment, in developing and piloting and reviewing instruments, in dealing with workforce turnover, using top-down, bottom-up or combined approach, trying to include all stakeholder groups, strategies to overcome resistance by storytelling and providing hard data, creating a collective purpose as a cultural frame, creating meaning or incentives for staff (e.g. alignment of staff incentives with organizational goals, giving staff members a feeling of ownership of a new program and the ability to adapt the model to meet their needs).	Ashton, 2015; Cambon et al., 2012; Cambon et al., 2013; Carter et al., 2009; Dixon-Woods et al., 2011; Feldstein & Glasgow, 2008; Kelly et al., 2000; Saurman et al., 2014; Trompette et al., 2014; Van Royen et al., 2014; Wang et al., 2005.
…identification and addressing of implementation barriers<br>e.g. through theoretical and experience-based strategies or by a local needs assessment for detecting aspects of the intervention which could not be implemented and necessary adaptation of the intervention (e.g. in terms of patient burden such as costs and complexity for responding and barriers among non-responders or barriers among frontline staff)<br>***and facilitators***<br>e.g. conditions for adoption and implementation (e.g. by a local needs assessment).	Ashton, 2015; Carter et al., 2009; Dixon-Woods et al., 2011; Feldstein & Glasgow, 2008; Glasgow et al., 2003; Kelly et al., 2000; Kilbourne et al., 2007; Watts et al., 2011.
…strategies of service delivery/intervention delivery<br>e.g. organizational and structural considerations/way of service organization such as devolution of responsibility to communities, dealing with waiting time, distribution of resources, efficiency, systematic collection of data, quality control in service delivery.	Carter et al., 2009; Dixon-Woods et al., 2011; Feldstein & Glasgow, 2008; Rychetnik et al., 2002; Saurman et al., 2014.
…successful pilot-testing of the intervention<br>e.g. for trying the intervention and reversing it if needed (e.g. in terms of feasibility, usability of tools for professionals and/or recipients or of a critical path that recipients can use to follow through with advice).	Carter et al., 2009; Feldstein & Glasgow, 2008; Kilbourne et al., 2007; Rychetnik et al., 2002; Saurman et al., 2014.

…possibility of adaptations throughout the intervention's process, i.e. of the implementation process and/or intervention form by keeping essential (core) elements<br>e.g. by mechanisms for rapid feedback and adaptation (e.g. regarding diagnosis, project approach, management and intervention activities, such as in terms of emerging new features).	Cambon et al., 2013; Dixon-Woods et al., 2011; Feldstein & Glasgow, 2008; Villeval et al., 2016; Watts et al., 2011.
**Descriptive theme:**<br>4.4 Characteristics of the evaluation in the target context in comparison to the primary context in terms of…	
**Criteria:**	**Authors:**
…evaluation/study design<br>e.g. efficacy versus effectiveness study, replication or dissemination study/translation research in primary and target context; study design such as RCT for probability assessment, (e.g. cluster RCT) or quasi-experimental study (with a control group for plausibility assessment), or an observational study/ case study research for adequacy assessment (i.e., did the expected changes occur?).	Cambon et al., 2012; Carter et al., 2009; Glasgow et al., 2003; Kilbourne et al., 2007; Muhlhausen, 2012; Rychetnick et al., 2012; Rychetnick et al., 2002.
…kind of assessment of processes and outcomes for measuring intervention success<br><br>**for example:**	Ashton, 2015; Cambon et al., 2012; Carter et al., 2009; Dixon-Woods et al., 2011; Kilbourne et al., 2007; Muhlhausen, 2012; Rychetnick et al., 2012; Tham et al., 2011.
population and patient/recipient-level outcomes:<br>e.g. health outcomes, outcomes relevant for stakeholders and recipients, intended/unintended effects, consideration of effect modification regarding health outcomes, e.g. to detect (statistical) interactions between intervention and contextual factors, consideration of statistical mediation (e.g. contextual mediating influences on outcome).	Cambon et al., 2012; Carter et al., 2009; Glasgow et al., 2003; Glasgow et al., 1999; Kilbourne et al., 2007; Muhlhausen, 2012; Perleth, 2009; Rychetnik et al., 2002; Schoenwald & Hoagwood, 2001; Weinmann et al., 2012.
participation rate or reach:<br>e.g. to population subgroups, participation of settings/organizations.	Cambon et al., 2013; Carter et al., 2009; Feldstein & Glasgow, 2008; Glasgow et al., 2003; Glasgow et al., 1999; Rychetnick et al., 2012.

public health impact:<br>in terms of reach (participation rate and the representativeness of participants), outcomes/effectiveness (impact of an intervention on specified outcome criteria, negative outcomes and intended results), adoption (percentage and representativeness of organizations or settings that conduct the intervention), implementation (intervention integrity, or the quality and consistency of delivery) and maintenance of the intervention (individual level long term results; setting level institutionalizing of the intervention) (RE-AIM).	Feldstein & Glasgow, 2008; Glasgow et al., 2003; Glasgow et al., 1999; Rychetnick et al., 2012.
interpretative/qualitative evaluation of the intervention implementation process:<br>e.g. through observation, ethnographic approach, description of assumed interaction between population, environment and intervention to help to explain outcomes, to explore community impact and sustainability of services<br><br>and/or quantitative process evaluation:<br>e.g. to assess acceptability of the intervention (such as the extend of adoption), monitor trends over the period of the study or to obtain information about health service utilization, satisfaction and need.	Ashton, 2015; Cambon et al., 2012; Carter et al. 2009; Dixon-Woods et al., 2011; Feldstein & Glasgow, 2008; Kilbourne et al., 2007; Rychetnick et al., 2012; Rychetnik et al., 2002; Tham et al., 2011; Watts et al., 2011.
evaluation of intervention fidelity, essential intervention elements and/or theory:<br>e.g. assessment of performance indicators for intervention fidelity and/or fidelity of implementation, testing or updating of program theory through statistical testing of a-priori formulated causal pathways based on information of primary and target context, through theory-oriented evaluation such as theory-based evaluation or theory-driven evaluation, ex-post theory for explaining how and why an intervention works, and adaptation (e.g. emerging successful forms of intervention activities, adaptation for wide scale delivery, expansion across borders, scalability).	Cambon et al., 2012; Carter et al. 2009; Dixon-Woods et al., 2011; Feldstein & Glasgow, 2008; Glasgow et al., 2003; Granstrom Ekeland & Grottland, 2015; Kilbourne et al., 2007; Muhlhausen, 2012; Pawson, 2003; Watts et al., 2011; Weinmann et al., 2012; Rychetnick et al., 2012.

health economic evaluation:<br>e.g. return on investment/cost-benefit/cost-effectiveness, financial and incidental costs.	Buffet et al., 2007; Kilbourne et al., 2007; Saurman et al., 2014; Weinmann et al., 2012.
**Criterion:**	**Authors:**
…similarity of determination of effects of the primary and replicated intervention<br>e.g. consideration of the difference between efficacy and effective-ness studies for contextual evaluation of effects; similarity of anal-yses.	Cambon et al., 2012; Chase et al., 2009; Guegan et al., 2011; Kilbourne et al., 2007.
**Criterion:**	**Authors:**
***…continuity and quality of evaluation throughout the transfer process* with regard to the**<br>**Sub-criteria:**	
• *kind and validity of information of the target context*<br>e.g. of baseline data by using the best available evidence for transferability assessment.	Schoenwald & Hoagwood, 2001; Wang et al., 2005; Watts et al., 2011.
• *validity and reliability of measures*<br>e.g. indicators for the health problem and goals, which can be reliably and validly operationalized for the target setting; sensi-tivity of measures to provide feedback on process and outcomes.	Dixon-Woods et al., 2011; Rychetnik et al., 2002; Tham et al., 2011.
• *continuity of monitoring and measuring success throughout the process*<br>e.g. for data flow, for periodic performance review and adjust-ments, for intervention adherence/fidelity and quality.	Cambon et al., 2013; Carter et al., 2009; Feldstein & Glas-gow, 2008; Kilbourne et al., 2007; Rychetnik et al., 2002; Schoenwald & Hoagwood, 2001; Tham et al., 2011; Ville-val et al., 2016.

Descriptive theme:<br>4.5 Characteristics of sustainability in the target context in comparison to the primary context in terms of…	
**Criterion:**	**Author:**
***…sustainability* with regard to the**<br>**Sub-criteria:**	
• *intervention outcomes*<br>e.g. intermediate and primary outcomes.	Burchett et al., 2011; Kilbourne et al., 2007; Muhlhausen, 2012; Watts et al., 2011.
• *change of current practice/stability and sustainability of implementation*	Burchett et al., 2011; Feldstein & Glasgow, 2008; Kilbourne et al., 2007; Rychetnik et al., 2002; Watts et al., 2011.
• *key factors in intervention success*<br>e.g. for dissemination, sustainability of factors in different settings.	Carter et al., 2009; Rychetnik et al., 2002; Weinmann et al., 2012.
• *stability of financing*	Kelly et al., 2000; Kilbourne et al., 2007; Villeval et al., 2016.
**Legend:** Descriptive themes and criteria underlie the higher-order themes population, intervention, environment and transfer, which are numbered from 1-4. The descriptive themes are numbered after each higher-order theme to facilitate the attribution to the higher-order theme. All criteria of transferability of health interventions relate to specific descriptive themes. Sub-criteria characterize a criterion in the form of specific aspects relevant to transferability. Criteria are written in bold letters. Both criteria and sub-criteria are highlighted in italics. Examples are written in normal letters.	

References of included articles

Ashton, T. (2015). Implementing integrated models of care: the importance of the macro-level context. *Int J Integr Care, 15*, e019.

Buffet, C., Ciliska, D., & Thomas, H. (2007). *Can I Use This Evidence in my Program Decision? Assessing Applicability and Transferability of Evidence*. Hamilton, ON L8S 1G5: National Collaborating Centre for Methods and Tools.

Burchett, H., Umoquit, M., & Dobrow, M. (2011). How do we know when research from one setting can be useful in another? A review of external validity, applicability and transferability frameworks. *J Health Serv Res Policy, 16*(4), 238-244.

Cambon, L., Minary, L., Ridde, V., & Alla, F. (2012). Transferability of interventions in health education: a review. *BMC Public Health, 12*, 497.

Cambon, L., Minary, L., Ridde, V., & Alla, F. (2013). A tool to analyze the transferability of health promotion interventions. *BMC Public Health, 13*, 1184.

Carter, M., Karwalajtys, T., Chambers, L., Kaczorowski, J., Dolovich, L., Gierman, T., . . . Laryea, S. (2009). Implementing a standardized community-based cardiovascular risk assessment program in 20 Ontario communities. *Health Promot Int, 24*(4), 325-333.

Chase, D., Rosten, C., Turner, S., Hicks, N., & Milne, R. (2009). Development of a toolkit and glossary to aid in the adaptation of health technology assessment (HTA) reports for use in different contexts. *Health Technol Assess, 13*(37), 1-142.
Cuijpers, P., Graaf, I., & Bohlmeijer, E. (2005). Adapting and disseminating effective public health interventions in another country: towards a systematic approach. *Eur J Public Health, 15*(2), 166–169.
Dixon-Woods, M., Bosk, C. L., Aveling, E. L., Goeschel, C. A., & Pronovost, P. J. (2011). Explaining Michigan: Developing an ex post theory of a quality improvement program. *Milbank Q, 89*(2), 167–205.
Feldstein, A. C., & Glasgow, R. E. (2008). A Practical, Robust Implementation and Sustainability Model (PRISM) for Integrating Research Findings into Practice. *Jt Comm J Qual Patient Saf, 34*(4), 228–243.
Glasgow, R. E., Lichtenstein, E., & Marcus, A. C. (2003). Why don't we see more translation of health promotion research to practice? Rethinking the efficacy-to-effectiveness transition. *Am J Public Health, 93*(8), 1261–1267.
Glasgow, R., Vogt, T., & Boles, S. (1999). Evaluating the public health impact of health promotion interventions: the RE-AIM framework. *Am J Public Health, 89*(9), 1322–1327.
Granstrøm Ekeland, A. G., & Grottland, A. (2015). Assessment of MAST in European patient-centered telemedicine pilots *Int J Technol Assess Health Care, 31*(5), 304-311.
Guegan, E., Milne, R., Pordage, A., Chase, D., Hicks, N., Bunce, H., . . . Payne, L. (2011). EUnetHTA HTA Adaptation toolkit Workpackage 5. Retrieved from http://www.eunethta.eu/outputs/eunethta-hta-adaptation-toolkit
Heller, R. F., Verma, A., Gemmell, I., Harrison, R., Hart, J., & Edwards, R. (2008). Critical appraisal for public health: a new checklist. *Public Health, 122*(1), 92-98.
Kelly, J. A., Heckman, T. G., Stevenson, L. Y., Williams, P. N., Ertl, T., Hays, R. B., . . . Neumann, M. S. (2000). Transfer of research-based HIV prevention interventions to community service providers: fidelity and adaptation. *AIDS Educ Prev, 12*(5 Suppl), 87–98.
Kidholm, K., Ekeland, A. G., Jensen, L. K., Rasmussen, J., Pedersen, C. D., Bowes, A., . . . Bech, M. (2012). A model for assessment of telemedicine applications: mast. *Int J Technol Assess Health Care, 28*(1), 44–51.
Kilbourne, A. M., Neumann, M. S., Pincus, H. A., Bauer, M. S., & Stall, R. (2007). Implementing evidence-based interventions in health care: application of the replicating effective programs framework. *Implement Sci, 2*, 42.
Muhlhausen, D. B. (2012). Evaluating Federal Social Programs: finding out what works and what does not. *Res Soc Work Pract, 22*(1), 100-107.
Pawson, R. (2003). Nothing as practical as a good theory. *Evaluation (Lond), 9*(4), 471–490.
Pearson, M., Parkin, S., & Coomber, R. (2011). Generalizing applied qualitative research on harm reduction: the example of a public injecting typology. *Contemp Drug Probl, 38*(1), 61–91.
Perleth, M. (2009). Assessment of the generalisability of clinical trial results in the Federal Joint Committee. [German]. *Z Evid Fortbild Qual Gesundhwes, 103*(6), 412-414.
Rychetnik, L., Bauman, A., Laws, R., King, L., Rissel, C., Nutbeam, D., . . . Caterson, I. (2012). Translating research for evidence-based public health: key concepts and future directions. *J Epidemiol Community Health, 66*(12), 1187–1192.
Rychetnik, L., Frommer, M., Hawe, P., & Shiell, A. (2002). Criteria for evaluating evidence on public health interventions. *J Epidemiol Community Health, 56*(2), 119-127.

Saurman, E., Johnston, J., Hindman, J., Kirby, S., & Lyle, D. (2014). A transferable telepsychiatry model for improving access to emergency mental health care. *J Telemed Telecare, 20*(7), 391–399.
Schoenwald, S. K., & Hoagwood, K. (2001). Effectiveness, transportability, and dissemination of interventions: what matters when? *Psychiatr Serv, 52*(9), 1190–1197.
Schreyogg, J. (2004). Justice in health care systems from an economic perspective. *Gesundheitswesen, 66*(1), 7-14.
Spencer, L. M., Schooley, M. W., Anderson, L. A., Kochtitzky, C. S., DeGroff, A. S., Devlin, H. M., & Mercer, S. L. (2013). Seeking best practices: a conceptual framework for planning and improving evidence-based practices. *Prev Chronic Dis, 10*, E207.
Tham, R., Humphreys, J. S., Kinsman, L., Buykx, P., Asaid, A., & Tuohey, K. (2011). Study protocol: evaluating the impact of a rural Australian primary health care service on rural health. *BMC Health Serv Res, 11*, 52.
Trompette, J., Kivits, J., Minary, L., Cambon, L., & Alla, F. (2014). Stakeholders' perceptions of transferability criteria for health promotion interventions: a case study. *BMC Public Health, 14*, 1134.
Van Royen, P., Rees, C. E., & Groenewegen, P. (2014). Patient-centred interprofessional collaboration in primary care: challenges for clinical, educational and health services research. An EGPRN keynote paper. *Eur J Gen Pract, 20*(4), 327-332.
Villeval, M., Bidault, E., Shoveller, J., Alias, F., Basson, J.-C., Frasse, C., . . . Lang, T. (2016). Enabling the transferability of complex interventions: exploring the combination of an intervention's key functions and implementation. *Int J Public Health*.
Wang, S., Moss, J. R., & Hiller, J. E. (2005). Applicability and transferability of interventions in evidence-based public health. *Health Promot Int, 21*(1), 76–83.
Watts, P., Phillips, G., Petticrew, M., Harden, A., & Renton, A. (2011). The influence of environmental factors on the generalisability of public health research evidence: physical activity as a worked example. *Int J Behav Nutr Phys Act, 8* (128).
Wegscheider, K. (2009). Transferability of study results to health care practice: contribution of different qualitative and quantitative research approaches [German]. *Z Evid Fortbild Qual Gesundhwes, 103*(6), 381-387.
Weinmann, S., Gühne, U., Kösters, M., Gaebel, W., & Becker, T. (2012). Team-based community psychiatry: importance of context factors and transferability of evidence from studies [German]. *Nervenarzt, 83*(7), 825–831.
Whitley, R., Rousseau, C., Carpenter-Song, E., & Kirmayer, L. J. (2011). Evidence-based medicine: Opportunities and challenges in a diverse society. *Can J Psychiatry, 56*(9), 514–522.
