# Supplementary material for: Criteria for evaluating transferability of health interventions: a systematic review and thematic synthesis
Source: Implement Sci. 2018 Jun 26;13:88. doi: 10.1186/s13012-018-0751-8 (PMC6019740; doi:10.1186/s13012-018-0751-8)
Supplement: Supplementary file 6 — Detailed table of barriers and facilitators. Table S5. Comprehensive information on barriers and facilitators. (PDF 166 kb) [file 13012_2018_751_MOESM6_ESM.pdf]

Additional file 6: Detailed table of barriers and facilitators

Table S5 Comprehensive information on barriers and facilitators

Barriers with authors:	Facilitators with authors:
**Higher-order theme:**<br>1. Criteria of the population in the primary and target context	
**Descriptive theme:**<br>1.1 The population characteristics in the primary and target context	
**Barriers with authors:**	**Facilitators with authors:**
**Differences of characteristics of the population in academic settings (primary context) and community-based organizations** (e.g. patients in the target context with low income or co-morbidities and minorities) may make it difficult to ensure that the intervention fits the local setting (Kilbourne et al., 2007; Van Royen et al., 2014; Whitley et al., 2011).	**Individual treatment decisions between provider and patient/recipient** can and should not be replaced by decisions on collective benefits when an intervention is transferred (Perleth, 2009; Wegscheider, 2009). However, on the basis of studies of good quality collective benefits can be assumed when individual benefits are evident (Wegscheider, 2009).
**Higher-order theme:**<br>2. Criteria of the intervention in the primary and target context	
**Descriptive theme:**<br>2.1 Characteristics of the evidence base for comparison of primary and target context	
**Barriers with authors:**	**Facilitators with authors:**
**Lack of time, resources or academic knowledge of decision-makers for searching and appraising evidence** may lead to reduced assessments of potential studies/interventions for use in order to understand their transferability (Burchett et al., 2011; Pearson et al., 2011).	**A specification of the theoretical basis of the intervention in studies** may make underlying assumptions more explicit and facilitate determination of appropriateness of outcome measures and methods for analysis (Rychetnik et al., 2002).

<table>
<tr>
<td>A lack of evidence for addressing a health problem/intervention may hinder or reduce transferability assessment (e.g. on the individual level, when the physician and patient have to decide on a treatment) and/or require the generation of evidence (e.g. efficacy or effectiveness research before scaling up an intervention) (Wang et al., 2005; Wegscheider, 2009).</td>
<td rowspan="4">The usefulness of evidence for practice in determining transferability may be enhanced through:

- guidelines for transparent reporting of interventions (Rychetnik et al., 2012).

- linked sources to process and contextual information in published papers when information is too lengthy for publication (Wang et al., 2005).

- reviews which include research-tested and practice-based studies with a range of study designs, assess internal as well as external validity and attempt to explain why interventions sometimes work and sometimes do not in different contexts in order to refine elements of intervention success or theory and to take into account issues of relevance and transferability to improve recommendations for practice (e.g. by realist synthesis, e.g. integration of knowledge from RCTs and meta-analyses for risk reduction, cohort studies for baseline risk, surveys for quality of life and qualitative approaches for facilitating life with a disease). Different evaluation approaches provide information needed for decision-making and enhance transferability (Rychetnik et al., 2002; Spencer et al., 2013; Watts et al., 2011; Wegscheider, 2009; Weinmann et al., 2012).

- more reporting of external validity and generalizability in studies to improve the quality of the evidence base and its usefulness to decision-makers (Burchett et al., 2011; Cambon et al., 2012; Kelly et al., 2000).

- more observational and quasi-experimental studies for evaluating public health interventions, which highly interact with context and population (“real world data”), although they have potential for bias (Cambon et al., 2012; Rychetnik et al., 2002; Van Royen et al., 2014; Wegscheider, 2009).</td>
</tr>
<tr>
<td>Small-scale studies with selected target groups and limited range of settings (Muhlhausen, 2012; Rychetnik et al., 2012) may make it difficult to anticipate suitability, practicability and costs for population wide dissemination (Rychetnik et al., 2012) and are not sufficient to inform policy-makers, as it is unsure if the intervention will achieve similar success on a large scale (Muhlhausen, 2012).</td>
</tr>
<tr>
<td>In general, conclusions from reviewing evidence for transferability may be limited due to
- poor description of an intervention (Rychetnik et al., 2012)
- a lack of adequate process and contextual information (Rychetnik et al., 2002; Wang et al., 2005)
- limited data
- unknown suitability of an intervention for different settings or different populations, e.g. for disadvantaged groups (Rychetnik et al., 2012; Rychetnik et al., 2002).</td>
</tr>
<tr>
<td>Statistics may not allow conclusions about transferability of an intervention for (sub-) groups or specific persons due to sample characteristics, reduced power for sub-group-analyses, the probability of results, too much concentration on p-values and means (e.g., in RCTs and meta-analyses) and reduced consideration of variances to understand different reactions of persons regarding an intervention (Wegscheider, 2009).</td>
</tr>
</table>

**A lack of a detailed description of what an intervention comprises (key elements and adaptations), how its activities are linked to its outcomes, and how context and intervention interact (theory why the intervention works effectively)** may lead to a reduced understanding of the intervention for transferability, to a limited ability for improvement of the intervention and the outcomes and to reduced possibilities for the comparison of primary and target intervention after transfer (Dixon-Wood et al., 2011).	**- more research on different stages and levels** (intervention testing/evaluation of efficacy and/or effectiveness, replication research in new/further settings and dissemination research at a larger scale) (Glasgow et al., 2003; Rychetnik et al., 2012; Schoenwald & Hoagwood, 2001), depending on the character and objective of the intervention (Cambon et al., 2012; Rychetnik et al., 2002), for example conducting RCT of a community intervention after investigation of feasibility of the program with cheaper designs (Rychetnik et al., 2002), large-scale, multisite high quality studies to determine if a program that works in one location or with one population works in other contexts before labeling it as "evidence-based" (e.g. to inform policy-makers) (Muhlhausen, 2012) or international comparative research focusing on the organization of care on different levels for learning from different health care systems (Van Royen et al., 2014).
**A lack of empirical investigations on core elements of an intervention** may require (more) conceptual considerations of the intervention in decision-making (Kelly et al., 2000).	
**Randomized controlled trials/efficacy studies in a classical design**<br>may make it difficult to anticipate transferability of complex (Public Health) interventions due to controlled conditions (strong internal validity but weak external validity), such as requirements for population characteristics (e.g. exclusion of people with comorbidities and polypharmacy or not representing ethnocultural diversity), difficulties to accommodate complexity and flexibility, more clinical training and support, higher intervention intensity, and limited descriptions of the intervention and of its adaptability to real contexts, although they are the highest-rated evaluation method and provide best evidence for determination of causality (Cambon et al., 2012; Glasgow et al., 2003; Glasgow et al., 1999; Kelly et al., 2000; Rychetnik et al., 2012; Rychetnik et al., 2002; Schoenwald & Hoagwood, 2001; Spencer et al., 2013; Van Royen et al., 2014; Villeval et al., 2016; Whitley et al., 2011).	**- more translational research comprising replication or dissemination research in new settings**, e. g. randomized controlled trails (RCTs)/ cluster-RCTs for high-level evidence in different/new settings (communities, regions) (Carter et al., 2009; Muhlhausen, 2012; Rychetnik et al., 2002; Weinmann et al., 2012) and case study research (Rychetnik et al., 2012) to determine what essential (core) elements of an effective intervention should be reproduced and what aspects/forms may be modified for transferability on small and large scale and to overcome the gap between research and practice, e.g. with research- based interventions which are conducted under circumstances similar to those that confront service providers in the field for less need to adapt interventions for field use (Carter et al., 2009; Kelly et al., 2000; Rychetnik et al., 2012; Weinmann et al., 2012).<br><br>**- evaluating approaches to replication and dissemination research** to determine if different models result in different outcomes, acceptability and cost-effectiveness (Rychetnik et al., 2012).

**Effect modification (of intervention components and/or contextual factors)** may affect the transferability of the intervention (Perleth, 2009; Rychetnik et al., 2002), make an assessment of transferability more difficult and complicate attempts to pool the results of different studies (Rychetnik et al., 2002). Moderating or mediating factors should be sought, understood and explained in studies (Glasgow et al., 2003; Rychetnik et al., 2002; Schoenwald & Hoagwood, 2001).	**- inclusion of qualitative approaches (e.g. observational and/or ethnographic) in (process) evaluation** which help to adapt the intervention to population needs, explain indicators, determinants of health and intervention outcomes (what and how it works), and build a sound basis for informing policy and practice in terms of transferability by exploring possible interactions among population, environment and intervention, which could be tested in further research (e.g. RCT) (Cambon et al., 2012; Carter et al., 2009; Dixon-Wood et al., 2011; Kelly et al., 2000; Pearson et al., 2011; Tham et al., 2011; Rychetnik et al., 2012; Rychetnik et al., 2002; Van Royen et al., 2014; Watts et al., 2011).<br><br>**- consideration of conditions relevant to practice in the development and conduction of studies**, e.g. regarding a treatment in efficacy studies (Perleth, 2009; Rychetnik et al., 2002).
**Without consideration of contextual factors and intervention components (core elements) thought to be responsible for intervention effects in systematic reviews of complex interventions**, **conclusions and practical recommendations for transfer might be biased** due to incomparability of the studies (e.g. different control conditions and intervention models) and insufficient description of relevant information for transferability (Weinmann et al., 2012).	
**Research on an intervention may not be sufficient to answer questions on potential effectiveness and transferability of interventions which need substantial adaptation in the target context** and require closer collaboration between researchers and organizations (Kelly et al., 2000).	
**Initial effectiveness studies may overlook or minimize relevant factors for transferability**, although effectiveness studies provide data from real world settings (Schoenwald & Hoagwood, 2001).	**Case studies may be helpful to explore similarities and differences between primary and target contexts**, particularly when they are followed by larger quasi-experimental or experimental studies, e.g. in including a large enough sample of organizations to detect contextual influences and issues of intervention fidelity (Schoenwald & Hoagwood, 2001).

**Descriptive theme:**<br>2.2 Characteristics of the intervention content in the primary and target context	
**Barriers with authors:**	**Facilitators with authors:**
**Reduced fidelity to the intervention and/or implementation** (Kilbourne et al., 2007; Muhlhausen, 2012; Weinmann et al., 2012)<br>**as well as a lack of guidance in customizing the intervention to the population** may act as barriers to effectiveness of the intervention (Kilbourne et al., 2007).	**Discussion on and identification of an intervention's key functions/core elements and specific context-relevant forms of the intervention for flexibility, innovation and adaptation,** may enable formulation of potentially transferable elements, key success-factors as well as elements, which should be modified in order to facilitate implementation in a context, to maintain the integrity of the intervention, and to enhance evaluation of intervention effects (Carter et al., 2009; Kelly et al., 2000; Villeval et al., 2016; Weinmann et al., 2012).
**Low program/intervention flexibility** may lead to a professional view of the intervention being unacceptable or not suited to local needs and constraints (Trompette et al., 2014).	**The identification of core elements which are central to effectiveness of an intervention may be facilitated by**<br>- definition of core elements, e.g. in terms of specific activities in sessions with individual clients or community intervention techniques for the overall program<br>- evaluation of theoretical elements of an intervention<br>- experience with the intervention, e.g. participants' and professionals' reactions and feedback about the intervention activities that they found especially useful<br>- testing different effects of the intervention with a variation of intervention components/elements to identify key components (formal component analysis), e.g. duration of the intervention (Kelly et al., 2000).
	**Decision on specific forms/adaptations of the intervention by local actors** may positively influence interaction between the intervention and its context (Villeval et al., 2016).

**Higher-order theme:**<br>3. Criteria of the environment in the primary and target context	
**Descriptive theme:**<br>3.1 Characteristics of policy and legislation in the primary and target context	
**Barriers with authors:**	**Facilitators with authors:**
	**Clear transferability criteria can facilitate political decisions regarding the transferability of an intervention** (e.g. on a national level) (Perleth, 2009).
**Descriptive theme:**<br>3.2 Characteristics of coordination players in the primary and target context	
**Barriers with authors:**	**Facilitators with authors:**
**Power inequalities between or within participating groups** may lead to marginalization of voices or interests (Dixon-Woods et al., 2011).	**Reflexive attitude of leaders towards practice** may help tailoring the intervention to communities, to build an actor network and shared mindset with different stakeholders, translate priorities and create innovation in order to foster success (Villeval et al., 2016).
**Protagonistic and antagonistic views in regarding the health issue as a social problem** may lead to resistance by antagonists (Dixon-Wood et al., 2011).	
**Descriptive theme:**<br>3.3 Characteristics of the health care system and service provision in the primary and target context	
**Barriers with authors:**	**Facilitators with authors:**
**Differences in conditions of service provision (e.g. treatment as usual, varying infrastructure) in the primary context and target context (e.g. in transferring an intervention between countries or from academic context to community-based organizations)** (Kelly et al., 2000; Kidholm et al., 2012; Kilbourne et al., 2007; Saurman et al., 2014; Weinmann et al., 2012; Whitley et al, 2010)<br>- may lead to reduced comparability for determination of effects for transferability (Weinmann et al., 2012; Whitley et al, 2010), and to a lack of resources for the intervention in the target context (e.g. budget, staff, electronic medical records, space) (Kelly et al., 2000; Kilbourne et al., 2007)	**The availability of alternative interventions in the health care system** may reduce the need for transfer of the intervention despite effectiveness of primary evidence (Weinmann et al., 2012).

- and may create substantial differences in the possibilities and costs per patient (Kidholm et al., 2012) or require specific local solutions (Saurman et al., 2014). Treatment as usual should therefore be regarded as an own treatment, which should be defined and described in detail for assessment of transferability (Weinmann et al., 2012).	
**Descriptive theme:**<br>3.4 Characteristics of the local and organizational setting in the primary and target context	
**Barriers with authors:**	**Facilitators with authors:**
**Ignoring/Not meeting stakeholders'/professionals' cultural, social, or professional expectations** may lead to lower involvement in the intervention (Trompette et al., 2014).	**Staffing levels which allow involvement of existing professionals in piloting and intervention delivery** may facilitate transfer (Feldstein & Glasgow, 2008).
**Team instability** (e.g. turnover rate) (Kelly et al., 2000; Trompette et al., 2014)<br>may lead to<br>- brake-down of team dynamic<br>- need to develop relationships of trust<br>- need to develop new team processes<br>- hindrance to the implementation<br>(Trompette et al., 2014).	**Acceptance of an intervention by professionals** (Dixon-Woods et al., 2011; Trompette et al., 2014) may be enhanced when they<br><br>- work on the planned action beforehand (Trompette et al., 2014)<br><br>- are involved and come up with suggestions (i.e. their views are taken into account, e.g. with community-based participatory approaches) (Dixon-Wood et al., 2011; Trompette et al., 2014)<br><br>- take initiative (e.g. communication and interaction) (Dixon-Wood et al., 2011; Trompette et al., 2014)<br><br>- have an interest in the subject or health problem (e.g., they see it as relevant or as priority) (Dixon-Wood et al., 2011; Trompette et al., 2014)<br><br>- are supported in transfer (e.g. knowledge transfer, for learning from each other in teams) (Dixon-Wood et al., 2011; Trompette et al., 2014).
**Synergistic or antagonistic interventions (e.g. interventions or conditions pursuing the same objective)** may reduce desired effects of the new intervention and thus reduce transferability (Weinmann et al., 2012).	

Higher-order theme: 4. Criteria of transfer from the primary to the target context	
**Descriptive theme:** 4.1 Characteristics of communication in the target context in comparison to the primary context	
**Barriers with authors:**	**Facilitators with authors:**
**Barriers in making contact/communication with professionals** may hinder sufficient participation and involvement (Carter et al., 2009).	**Data transfer and data quality in community-based interventions** may be enhanced by a data transfer system which allows community-level responsibility, management by local coordinators, centralized services for data management and data queries for quality control (Carter et al., 2009).
	**Communication support by leaders (e.g. facilitating and moderating discussion of success and problems in meetings)** may help teams to make generalizable points and encourage to learn from another (Dixon-Wood et al., 2011).
	**The possibility for professionals to see positive results** may promote confidence and self-efficacy of professionals for implementation and sustainability (Feldstein & Glasgow, 2008).
	**Providing feedback to recipients** may enhance their continuity of participation and health behavior (Feldstein & Glasgow, 2008).
**Descriptive theme:** 4.2 Characteristics of knowledge transfer in the target context in comparison to the primary context	
**Barriers with authors:**	**Facilitators with authors:**
	**Support by intervention specialists in planning and implementation** may facilitate adaptation of the intervention to meet local and organizational needs and intervention success (Kelly et al. 2000).

	**Development and provision of suitable training, technical assistance as well as an intervention package (in collaboration of intervention experts and stakeholders) or implementation guide** e. g. containing<br>- specific objectives (over time)<br>- user friendly manual<br>- documents/materials<br>- key strategies or menue options for implementing intervention core elements and adaptation options<br>may facilitate intervention fidelity and flexibility and serve as a means to guide processes and monitor progress (Carter et al., 2009; Kelly et al., 2000; Kilbourne et al., 2007).
	**Knowledge exchange between professionals and researchers/intervention experts** (Feldstein & Glasgow, 2008; Kelly et al. 2000; Kilbourne et al., 2007; Trompette et al., 2014; Rychetnik et al., 2012; Schoenwald & Hoagwood, 2001; Van Royen et al., 2014; Villeval et al., 2016) may foster<br><br>- mutual learning and transferability (Dixon-Wood et al., 2011; Kelly et al. 2000; Rychetnik et al., 2012; Trompette et al, 2014; Van Royen et al., 2014; Villeval et al., 2016)<br><br>- theorizing the intervention, describing key functions of the intervention, the implementation and the context (Dixon-Wood et al., 2011; Kelly et al. 2000; Villeval et al., 2016)<br><br>- feasibility of the intervention and implementation (Dixon-Wood et al., 2011; Feldstein & Glasgow, 2008; Kilbourne et al., 2007)<br><br>- knowledge of implementation barriers and needs (e.g. assistance) (Kelly et al. 2000; Kilbourne et al., 2007)<br><br>- benchmarking of usual care (Kilbourne et al., 2007)<br><br>- buy-in when benefits of the intervention are made clear (e.g. cost-savings, training opportunities) (Kilbourne et al., 2007).

**Descriptive theme:**<br>4.3 Characteristics of adoption and implementation in the target context in comparison to the primary context	
**Barriers with authors:**	**Facilitators with authors:**
**Imposing the intervention on professionals without involvement in planning** (Dixon-Wood et al., 2011; Trompette et al., 2014) may lead to<br>- feeling of being obliged to participate (Trompette et al., 2014)<br>- violation of norms (e.g. of collegiality) (Dixon-Wood et al., 2011)<br>- feeling of additional burden and negative experiences (Trompette et al., 2014)<br>- reluctance to take part/resistance (Dixon-Wood et al., 2011; Trompette et al., 2014)<br>- detriment to success<br>(Dixon-Wood et al., 2011; Trompette et al., 2014).	**A local needs assessment** may facilitate the detection of non-transferable intervention elements and the need for adaptation of the intervention, e.g. in terms of resources or organizational priorities and needs (Kelly et al., 2000).
**Implementation barriers for professionals**<br>may be a lack of awareness or familiarity, lack of self-efficacy or outcome expectancy or inertia of previous practice (Feldstein & Glasgow, 2008).	**Addressing needs and barriers of professionals before implementing an intervention** and adapting it to improve usability may enhance effectiveness of the intervention (Feldstein & Glasgow, 2008).
	**Addressing participation barriers in the population,** such as seamless transitions between services and access issues, may reduce difficulties in following through with advice (Feldstein & Glasgow, 2008).
	**Personalized invitation strategies and appropriate materials and resources for the population** may be helpful to increase participation of the target population (e.g. invitation letters, information packages) (Carter et al., 2009).
	**Involving community members (e.g. volunteers) as ambassadors for the intervention** may be helpful to foster participation of the population and implementation in a target community (Carter et al., 2009).

	**Strategies involving support of (local) opinion leaders in a field** may mobilize professionals and increase their participation, e.g. by personalized letters or personal contact with colleagues (Carter et al., 2009).
**Descriptive theme:**<br>4.4 Characteristics of the evaluation in the target context in comparison to the primary context	
**Barriers with authors:**	**Facilitators with authors:**
**Identifying all relevant factors for determining transferability may be impossible.** Some factors will emerge over time and throughout the transfer process, e.g. moderators (Schoenwald & Hoagwood, 2001).	**Validity of information gathering in the target context for comparison of primary evidence and target context** can be enhanced by<br><br>- using/gathering best available evidence of the target context (e.g. available literature such as epidemiologic and demographic studies such as census data, using or conducting qualitative research/ Delphi-Study with stakeholders) (Wang et al., 2005; Watts et al., 2011)<br><br>- explicitly stating reasons behind judging for similarity of contexts (Wang et al., 2005).
**The operationalization of indicators for (process and outcome) measurements** may be difficult in terms of their applicability for routinely and unobtrusively collecting data in the target context and being beneficial to health outcomes and services (Tham et al., 2011).	**A structured documentation and ongoing evaluation of details of implementation processes (e.g. by templates and interviews)** may help to elicit facilitators, common success and barriers/ challenges in different contexts (e.g. to better understand how and why the intervention worked, how it can be improved) to understand issues of transferability (Carter et al., 2009; Tham et al., 2011).
**Evaluation of only the intended outcomes of an intervention** may not detect other positive or negative consequences and unintended program effects (Rychetnik et al., 2002).	**Identification and scientific confirmation of key factors/ (core) elements in successful intervention delivery** may enhance transferability of interventions, e.g. across communities (Carter et al., 2009; Pawson, 2003).

	**Updating a program/ intervention theory with leaders and scientists after transfer under consideration of experiences for explaining how an intervention worked** may be useful for formalizing hypotheses that can be further tested, for continuous feedback, for discussion circles, for ongoing learning, for improvement of the intervention and for decision-makers who seek to transfer the intervention (Dixon-Wood et al., 2011).
	**Addressing population impact in terms of reach, effectiveness, adoption, implementation and maintenance in population-wide studies** may enhance long term success (Feldstein & Glasgow, 2008).
**Descriptive theme:** 4.5 Characteristics of sustainability in the target context in comparison to the primary context	
**Unstable resources over time** (e.g. funding, staff turnover) may lead to uncertainty around continuation of the intervention and may influence results and sustainability (Kelly et al., 2000; Villeval et al., 2016).	**Consideration of sustainability of the intervention during implementation** may facilitate long-term maintenance (Feldstein & Glasgow, 2008).
**Legend:** The barriers and facilitators underlie the higher-order themes population, intervention, environment and transfer, which are numbered from 1-4. The descriptive themes are numbered after each higher-order theme to facilitate the attribution to the higher-order theme. Main points of barriers and facilitators are written in bold letters, consequences or further explanations in normal letters.	

References of included articles

Ashton, T. (2015). Implementing integrated models of care: the importance of the macro-level context. *Int J Integr Care, 15*, e019.

Buffet, C., Ciliska, D., & Thomas, H. (2007). *Can I Use This Evidence in my Program Decision? Assessing Applicability and Transferability of Evidence*. Hamilton, ON L8S 1G5: National Collaborating Centre for Methods and Tools.

Burchett, H., Umoquit, M., & Dobrow, M. (2011). How do we know when research from one setting can be useful in another? A review of external validity, applicability and transferability frameworks. *J Health Serv Res Policy, 16*(4), 238-244.

Cambon, L., Minary, L., Ridde, V., & Alla, F. (2012). Transferability of interventions in health education: a review. *BMC Public Health, 12*, 497.

Cambon, L., Minary, L., Ridde, V., & Alla, F. (2013). A tool to analyze the transferability of health promotion interventions. *BMC Public Health, 13*, 1184.

Carter, M., Karwalajtys, T., Chambers, L., Kaczorowski, J., Dolovich, L., Gierman, T., . . . Laryea, S. (2009). Implementing a standardized community-based cardiovascular risk assessment program in 20 Ontario communities. *Health Promot Int, 24*(4), 325-333.

Chase, D., Rosten, C., Turner, S., Hicks, N., & Milne, R. (2009). Development of a toolkit and glossary to aid in the adaptation of health technology assessment (HTA) reports for use in different contexts. *Health Technol Assess, 13*(37), 1-142.

Cuijpers, P., Graaf, I., & Bohlmeijer, E. (2005). Adapting and disseminating effective public health interventions in another country: towards a systematic approach. *Eur J Public Health, 15*(2), 166–169.

Dixon-Woods, M., Bosk, C. L., Aveling, E. L., Goeschel, C. A., & Pronovost, P. J. (2011). Explaining Michigan: Developing an ex post theory of a quality improvement program. *Milbank Q, 89*(2), 167–205.

Feldstein, A. C., & Glasgow, R. E. (2008). A Practical, Robust Implementation and Sustainability Model (PRISM) for Integrating Research Findings into Practice. *Jt Comm J Qual Patient Saf, 34*(4), 228–243.

Glasgow, R. E., Lichtenstein, E., & Marcus, A. C. (2003). Why don't we see more translation of health promotion research to practice? Rethinking the efficacy-to-effectiveness transition. *Am J Public Health, 93*(8), 1261–1267.

Glasgow, R., Vogt, T., & Boles, S. (1999). Evaluating the public health impact of health promotion interventions: the RE-AIM framework. *Am J Public Health, 89*(9), 1322–1327.

Granstrøm Ekeland, A. G., & Grottland, A. (2015). Assessment of MAST in European patient-centered telemedicine pilots *Int J Technol Assess Health Care, 31*(5), 304-311.

Guegan, E., Milne, R., Pordage, A., Chase, D., Hicks, N., Bunce, H., . . . Payne, L. (2011). EUnetHTA HTA Adaptation toolkit Workpackage 5. Retrieved from http://www.eunethta.eu/outputs/eunethta-hta-adaptation-toolkit

Heller, R. F., Verma, A., Gemmell, I., Harrison, R., Hart, J., & Edwards, R. (2008). Critical appraisal for public health: a new checklist. *Public Health, 122*(1), 92-98.

Kelly, J. A., Heckman, T. G., Stevenson, L. Y., Williams, P. N., Ertl, T., Hays, R. B., . . . Neumann, M. S. (2000). Transfer of research-based HIV prevention interventions to community service providers: fidelity and adaptation. *AIDS Educ Prev, 12*(5 Suppl), 87–98.

Kidholm, K., Ekeland, A. G., Jensen, L. K., Rasmussen, J., Pedersen, C. D., Bowes, A., . . . Bech, M. (2012). A model for assessment of telemedicine applications: mast. *Int J Technol Assess Health Care, 28*(1), 44–51.

Kilbourne, A. M., Neumann, M. S., Pincus, H. A., Bauer, M. S., & Stall, R. (2007). Implementing evidence-based interventions in health care: application of the replicating effective programs framework. *Implement Sci, 2*, 42.

Muhlhausen, D. B. (2012). Evaluating Federal Social Programs: finding out what works and what does not. *Res Soc Work Pract, 22*(1), 100-107.

Pawson, R. (2003). Nothing as practical as a good theory. *Evaluation (Lond), 9*(4), 471–490.

Pearson, M., Parkin, S., & Coomber, R. (2011). Generalizing applied qualitative research on harm reduction: the example of a public injecting typology. *Contemp Drug Probl, 38*(1), 61–91.

Perleth, M. (2009). Assessment of the generalisability of clinical trial results in the Federal Joint Committee. [German]. *Z Evid Fortbild Qual Gesundhwes, 103*(6), 412-414.

Rychetnik, L., Bauman, A., Laws, R., King, L., Rissel, C., Nutbeam, D., . . . Caterson, I. (2012). Translating research for evidence-based public health: key concepts and future directions. *J Epidemiol Community Health, 66*(12), 1187–1192.

Rychetnik, L., Frommer, M., Hawe, P., & Shiell, A. (2002). Criteria for evaluating evidence on public health interventions. *J Epidemiol Community Health, 56*(2), 119-127.
Saurman, E., Johnston, J., Hindman, J., Kirby, S., & Lyle, D. (2014). A transferable telepsychiatry model for improving access to emergency mental health care. *J Telemed Telecare, 20*(7), 391–399.
Schoenwald, S. K., & Hoagwood, K. (2001). Effectiveness, transportability, and dissemination of interventions: what matters when? *Psychiatr Serv, 52*(9), 1190–1197.
Schreyogg, J. (2004). Justice in health care systems from an economic perspective. *Gesundheitswesen, 66*(1), 7-14.
Spencer, L. M., Schooley, M. W., Anderson, L. A., Kochtitzky, C. S., DeGroff, A. S., Devlin, H. M., & Mercer, S. L. (2013). Seeking best practices: a conceptual framework for planning and improving evidence-based practices. *Prev Chronic Dis, 10*, E207.
Tham, R., Humphreys, J. S., Kinsman, L., Buykx, P., Asaid, A., & Tuohey, K. (2011). Study protocol: evaluating the impact of a rural Australian primary health care service on rural health. *BMC Health Serv Res, 11*, 52.
Trompette, J., Kivits, J., Minary, L., Cambon, L., & Alla, F. (2014). Stakeholders' perceptions of transferability criteria for health promotion interventions: a case study. *BMC Public Health, 14*, 1134.
Van Royen, P., Rees, C. E., & Groenewegen, P. (2014). Patient-centred interprofessional collaboration in primary care: challenges for clinical, educational and health services research. An EGPRN keynote paper. *Eur J Gen Pract, 20*(4), 327-332.
Villeval, M., Bidault, E., Shoveller, J., Alias, F., Basson, J.-C., Frasse, C., . . . Lang, T. (2016). Enabling the transferability of complex interventions: exploring the combination of an intervention's key functions and implementation. *Int J Public Health*.
Wang, S., Moss, J. R., & Hiller, J. E. (2005). Applicability and transferability of interventions in evidence-based public health. *Health Promot Int, 21*(1), 76–83.
Watts, P., Phillips, G., Petticrew, M., Harden, A., & Renton, A. (2011). The influence of environmental factors on the generalisability of public health research evidence: physical activity as a worked example. *Int J Behav Nutr Phys Act, 8* (128).
Wegscheider, K. (2009). Transferability of study results to health care practice: contribution of different qualitative and quantitative research approaches [German]. *Z Evid Fortbild Qual Gesundhwes, 103*(6), 381-387.
Weinmann, S., Gühne, U., Kösters, M., Gaebel, W., & Becker, T. (2012). Team-based community psychiatry: importance of context factors and transferability of evidence from studies [German]. *Nervenarzt, 83*(7), 825–831.
Whitley, R., Rousseau, C., Carpenter-Song, E., & Kirmayer, L. J. (2011). Evidence-based medicine: Opportunities and challenges in a diverse society. *Can J Psychiatry, 56*(9), 514–522.
